# Identification and characterisation of G-quadruplex DNA-forming sequences in the *Pseudomonas aeruginosa* genome†

**DOI:** 10.1039/d2cb00205a

**Published:** 2022-11-15

**Authors:** Lindsay Evans, Anita Kotar, Martina Valentini, Alain Filloux, Shirin Jamshidi, Janez Plavec, Khondaker Miraz Rahman, Ramon Vilar

**Affiliations:** a Department of Chemistry, Imperial College London, White City Campus London W12 OBZ UK r.vilar@imperial.ac.uk; b Slovenian NMR Center, National Institute of Chemistry, Hajdrihova 19 1000 Ljubljana Slovenia; c Department of Life Sciences, MRC Centre for Molecular Microbiology and Infection, Imperial College London, South Kensington Campus London SW7 2AZ UK; d Institute of Pharmaceutical Science, King's College London, Franklin-Wilkins Building 150 Stamford Street London SE1 9NH UK k.miraz.rahman@kcl.ac.uk

## Abstract

A number of Gram-negative bacteria such as *Pseudomonas aeruginosa* are becoming resistant to front-line antibiotics. Consequently, there is a pressing need to find alternative bio-molecular targets for the development of new drugs. Since non-canonical DNA structures such as guanine-quadruplexes (G4s) have been implicated in regulating transcription, we were interested in determining whether there are putative quadruplex-forming sequences (PQS) in the genome of *Pseudomonas aeruginosa*. Using bioinformatic tools, we screened 36 genes potentially relevant to drug resistance for the presence of PQS and 10 of these were selected for biophysical characterisation (*i.e.* circular dichroism and thermal difference UV/Vis spectroscopy). These studies showed that three of these G-rich sequences (linked to *murE*, *ftsB* and *mexC* genes) form stable guanine-quadruplexes which were studied by NMR spectroscopy; detailed analysis of one of the sequences (*mexC*) confirmed that it adopts a two-quartet antiparallel quadruplex structure in the presence of K^+^ ions. We also show by FRET melting assays that small molecules can stabilise these three new G4 DNA structures under physiological conditions. These initial results could be of future interest in the development of new antibiotics with alternative bio-molecular targets which in turn would help tackle antimicrobial resistance.

## Abstract 2

We report identification and characterisation of G-quadruplex DNA forming sequences in the *Pseudomonas aeruginosa* genome in genes associated with antimicrobial resistance.

## Graphical abstract

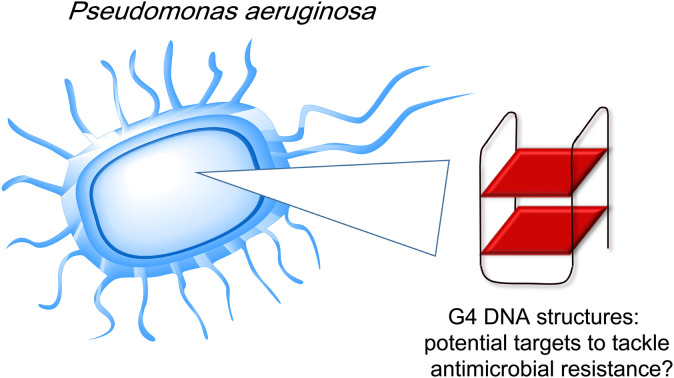

## Introduction

Recent research has shown that G-quadruplex DNA (G4 DNA) structures form in bacteria and could potentially be new targets for antibiotics.^1–7^ These tetra-stranded structures can readily form under physiological conditions from guanine-rich sequences due to guanines’ ability to display Hoogsteen hydrogen bonding interactions. Over the past few years, mounting experimental evidence has gathered indicating that G4 DNA structures form *in vivo*^8–13^ and play essential biological roles including regulation of gene expression, replication and telomere maintenance.^14–16^ Due to their proposed biological relevance, G-quadruplexes are attractive targets for the development of drugs.^9,17,18^ Therefore, there has been increasing interest in the development of small molecules able to bind to G4s with high affinity and selectivity. Most studies on G4s (and molecules developed to target them) thus far, have focused on eukaryotic cells and have been mainly aimed at developing new anti-cancer therapies.^9,17,18^

Bioinformatic studies have shown that G4 DNA structures are also prevalent in bacteria, particularly in gene promoters. In 2006, it was reported that the *Escherichia coli* genome contains *ca.* 3000 putative quadruplex-forming sequences (PQS).^19^ Subsequently a database was published outlining putative G4-forming sequences in 146 different microbial genomes.^20^ Some of these sequences in *Escherichia coli*,^21^*Deinococcus radiodurans*^22,23^ and *Mycobacterium tuberculosis*,^24^ have been found associated to promoter regions and hence implicated in regulation of gene expression. More recently, a G4 sequencing method was applied to identify *in vitro* 131 different G-quadruplexes in *Escherichia coli* and 1990 in *Rhodobacter*.^25^ Another recent study analysed PQSs in the genome of seven Gram-negative and five Gram-positive bacteria, and it showed that *Pseudomonas aeruginosa* has a particularly dense PQS prevalence.^26^ In this same study, the authors investigated the biological activity of a library of G4 DNA binders against both Gram-positive and Gram-negative bacteria. For their lead compound, they reported distinct mechanisms of action against Gram-positive and Gram-negative bacteria which they hypothesise could be attributed to the differences in prevalence of putative G4 sequences in each of them.

There is significant interest in finding alternative bio-molecular targets for the development of antibiotics, with particular emphasis in combating multidrug resistance. With this in mind, we carried out bioinformatics studies to identify guanine-rich sequences in the *Pseudomonas aeruginosa* genome associated to genes relevant to antimicrobial resistance (see below). *P. aeruginosa* is a Gram-negative bacterial pathogen associated with severe acute and chronic human diseases and is the leading cause of death in cystic fibrosis patients. This bacterium also affects immune-compromised patients in hospitals and infections of the blood, following surgery or pneumonia, resulting in high morbidity and mortality. The emergence of a number of Gram-negative pathogens that are resistant to the front-line antibiotic therapies is a global concern and the pipeline of antibiotics is essentially empty. In this respect, *P. aeruginosa* has been ranked by WHO in the top three of organisms that are critical and needing immediate attention. Therefore, it is urgent to identify and validate alterative biomolecular targets to develop new antibacterial agents capable of either killing these multi-drug resistant (MRD) bacteria or make them susceptible again against current antibiotics.

Herein we report studies on a series of G-rich sequences in *P. aeruginosa* genome with the potential to form G4 structures in regulatory/coding regions of genes relevant to antimicrobial resistance. Following a bioinformatics analysis, ten sequences were selected, and studied by circular dichroism (CD) and thermal difference UV/Vis spectroscopy (TDS) to determine their ability to fold into G-quadruplexes under physiological conditions. This allowed us to identify three sequences that readily fold into stable G4s under physiological conditions. These new G4s were further studied by NMR spectroscopy. The interactions of some previously reported binders with the new G4 structures were studied by FRET melting assays which confirm they can be stabilised by small molecules.

## Results and discussion

### Bioinformatic analysis for G4 in the *P. aeruginosa* genome.

To explore the role of G-quadruplexes on gene expression in natural genetic contexts, we investigated their occurrence in a total of 36 genes that have been proposed to play critical roles in *P. aeruginosa* antibiotic resistance and lifestyle regulation (Table S1, ESI†). The name and function of the genes studied are given according to the National Centre for Biotechnology Information's (NCBI) BLAST database. The nucleotide sequences of the genes were obtained from UniProt, EMBL-EBI or NCBI in FASTA format. As the position of a G4 sequence within the promoter, 5′-UTR, and 3′-UTR regions has been shown to influence gene expression,^21^ we included in our analysis the upstream regulatory regions (500 nucleotides upstream from the start codon) and 3′-UTR regions (250 nucleotides downstream from the stop codon) (SI) from our selected genes using the KEGG database.^27^

These sequences in FASTA format were then analysed using the QGRS mapping web server^28^ (https://bioinformatics.ramapo.edu/QGRS/analyze.php) with a minimum G-group size 2 to identify putative G4-forming sequences. The top ten sequences showing a minimum G-score of 19 (Table 1) were selected for biophysical and spectroscopic studies to establish whether G4 structures could be formed. The majority of genes selected for the biophysical studies (*e.g., murE*,^29^*amprR*,^30^*czcA*,^31^*mexC*,^32^*nfxB*,^33^*oprF*^34^ and *pvrR*^35^) have been associated with antibiotic resistance in *P. aeruginosa* and other ESKAPE pathogens (*i.e. Enterococcus faecium*, *Staphylococcus aureus*, *Klebsiella pneumoniae*, *Acinetobacter baumannii* and *Enterobacter species*, which with *P. aeruginosa* are the leading cause of nosocomial infections throughout the world).

**Table 1:** Sequences with the highest potential (G-score) to form G-quadruplex identified by the QGRS mapper web server^28^

Gene	G-Score	Position	Sequence	Function of corresponding proteins in *P. aureginosa*
*murE*	21	5′–UTR	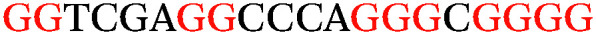	Amidase ligase involved in cell wall biosynthesis
*ftsB*	20	Putative promoter	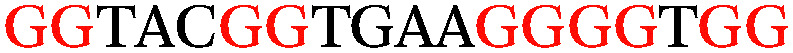	Cell division protein
*ampR*	19	Putative promoter	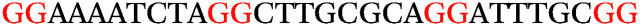	Regulator of β-lactamase/virulence regulator
*cbrB*	20	Coding sequence	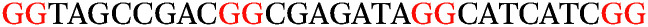	Instrumental in the maintenance of the carbon-nitrogen balance
*czcA*	20	Putative promoter	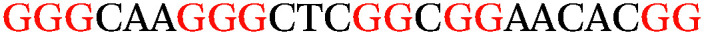	Heavy metal efflux pump
*mexC*	20	Coding sequence	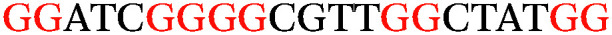	Part of the MexC-MexD-OprJ multidrug efflux pump system
*nfxB*	20	Coding sequence	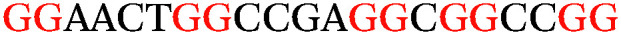	Involved in conferring resistance to quinolones
*oprF*	21	Coding sequence	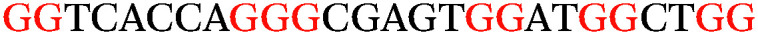	General porin that allows the nonspecific diffusion of ionic particles and polar nutrients
*pilE*	21	Coding sequence	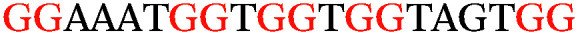	Subunit in a larger protein complex that mediates twitching motility, biofilm maturation, surface adhesion and virulence
*pvrR*	20	3′–UTR	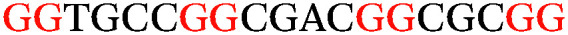	Regulatory protein that controls conversion between antibiotic-resistant and antibiotic-susceptible forms

### Biophysical characterisation of G-rich sequences identified by bioinformatics analysis.

**Fig. 1:**
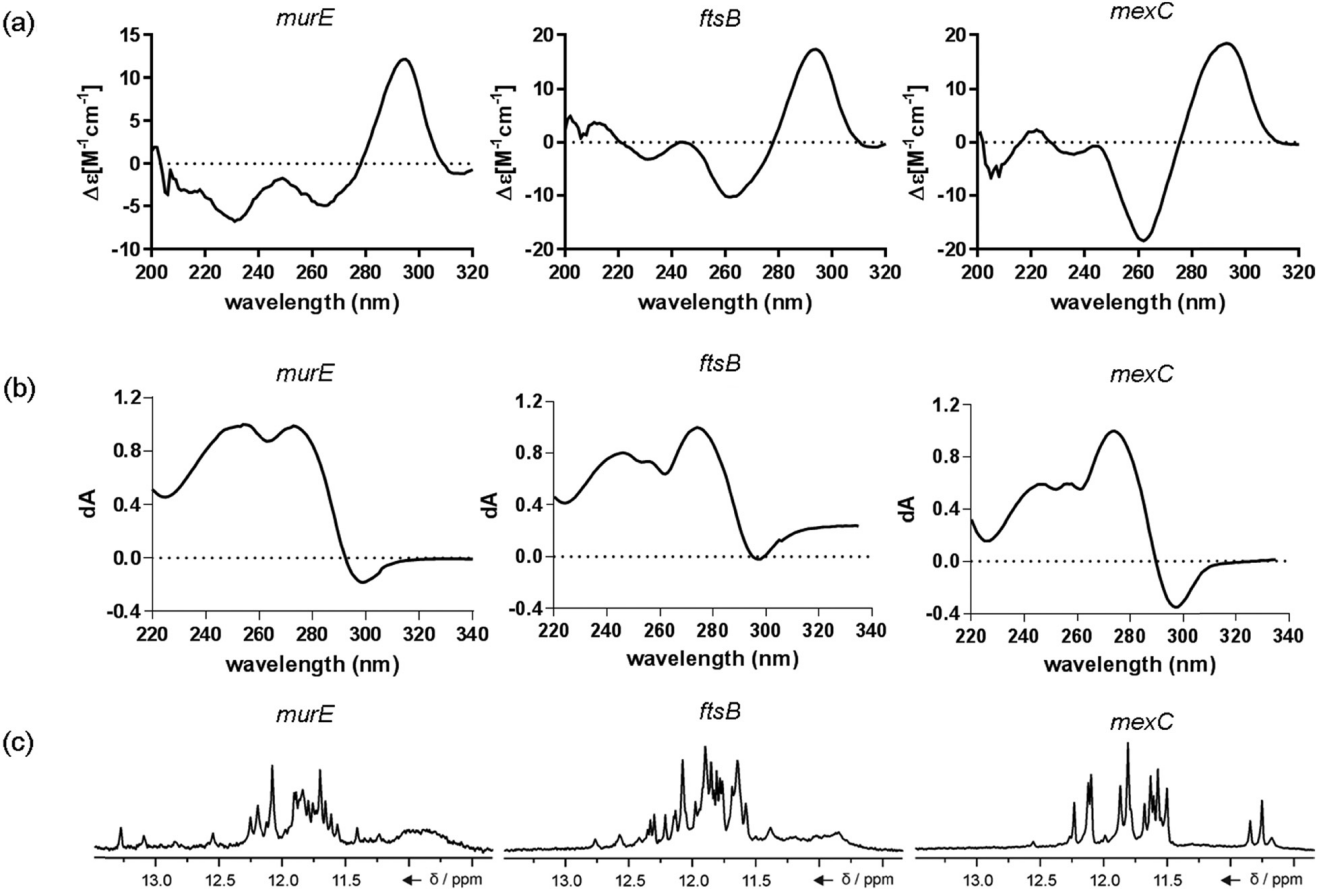
Spectroscopic characterisation of *murE*, *ftsB* and *mexC* G4-forming sequences. (a) CD spectra showing characteristic bands at 295 and 260 nm associated to G4 formation; (b) thermal difference spectra highlighting the expected pattern for G4 DNA formation. (c) Imino region of ^1^H NMR spectra of *murE*, *ftsB* and *mexC* oligonucleotides. NMR spectra were recorded at 0.4 mM concentration of DNA per strand, 100 mM KCl, pH 7.4, 25 °C, on a 600 MHz NMR spectrometer.

The ten putative quadruplex-forming sequences (PQS) identified in the *P. aeruginosa* genome (see Table 1 for the sequences as well as their position – *i.e.* promoter, coding sequence or UTRs), were studied by circular dichroism (CD) spectroscopy and thermal difference UV/Vis spectroscopy (TDS). When combined, the results from these two techniques give a very good indication of whether a given sequence folds into a G4 structure or not. Three of the sequences – named *murE*, *ftsB* and *mexC* according to the corresponding gene (see Table 1) – were shown to form G4 structures. For these sequences, the CD spectra recorded at room temperature showed the characteristic positive ellipticity band at *ca.* 295 nm and a negative one at *ca.* 260 nm associated to an antiparallel G4 DNA conformation (see Fig. 1a).^36,37^ The formation of the three G4 DNA structures was confirmed by the characteristic pattern in the TDS: positive bands at *ca.* 243 and 273 nm, and a negative one at *ca.* 295 nm (see Fig. 1b).^38^ In contrast, the combined analysis of the CD and TDS data (see Fig. S1, ESI†) for the other seven sequences under study, suggest they do not form stable G4 DNA structures. It should be noted that four of the sequences (*czcA*, *nfxB*, *oprF* and *pilE*) that did not show clear formation of G4 structures by CD and TDS, contain five rather than four G-tracks. This could potentially lead to more than one G4 structure forming depending which G-tracks are involved; this in turn could lead to ambiguous spectroscopic data as has been previously highlighted.^38^ While it would be possible to study these sequences using a truncated version of the PQS (*i.e.* with four G-tracks), we decided to focus our further studies on the sequences associated to genes *murE*, *ftsB* and *mexC* which clearly form stable G4s.

In full agreement, the ^1^H NMR spectra of *murE*, *ftsB* and *mexC* oligonucleotides revealed signals in the region between *δ* 11.0 and 12.5 ppm, which are characteristic for imino protons of guanine residues involved in G-quartets (Fig. 1c). Additionally, the signals between *δ* 12.5 and 13.3 ppm in the ^1^H NMR spectra indicate formation of Watson–Crick GC base pairs. In the case of *mexC* oligonucleotide sequence, three signals between *δ* 10.5 and 10.7 ppm were detected suggesting the presence of hydrogen bonded imino protons in non-Watson–Crick geometries. Monitoring the conformational rearrangement of the three oligonucleotides, in the presence of 0 to 100 mM concentrations of KCl, revealed that *murE* and *ftsB* sequences adopted defined pre-folded structures in the absence of K^+^ ions (Fig. S2, ESI†). After addition of KCl the pattern of imino signals changed in the ^1^H NMR spectra of *murE* and *ftsB* oligonucleotides showing only the presence of G4 structures, which remained constant at KCl concentrations ranging from 10 to 100 mM KCl. In the case of *mexC*, the intensity of imino signals increased as KCl was titrated into a solution of *mexC* with no other changes observed in the imino region that would be connected to structural changes.

### *mexC* oligonucleotide adopts a two-quartet G4

**Fig. 2:**
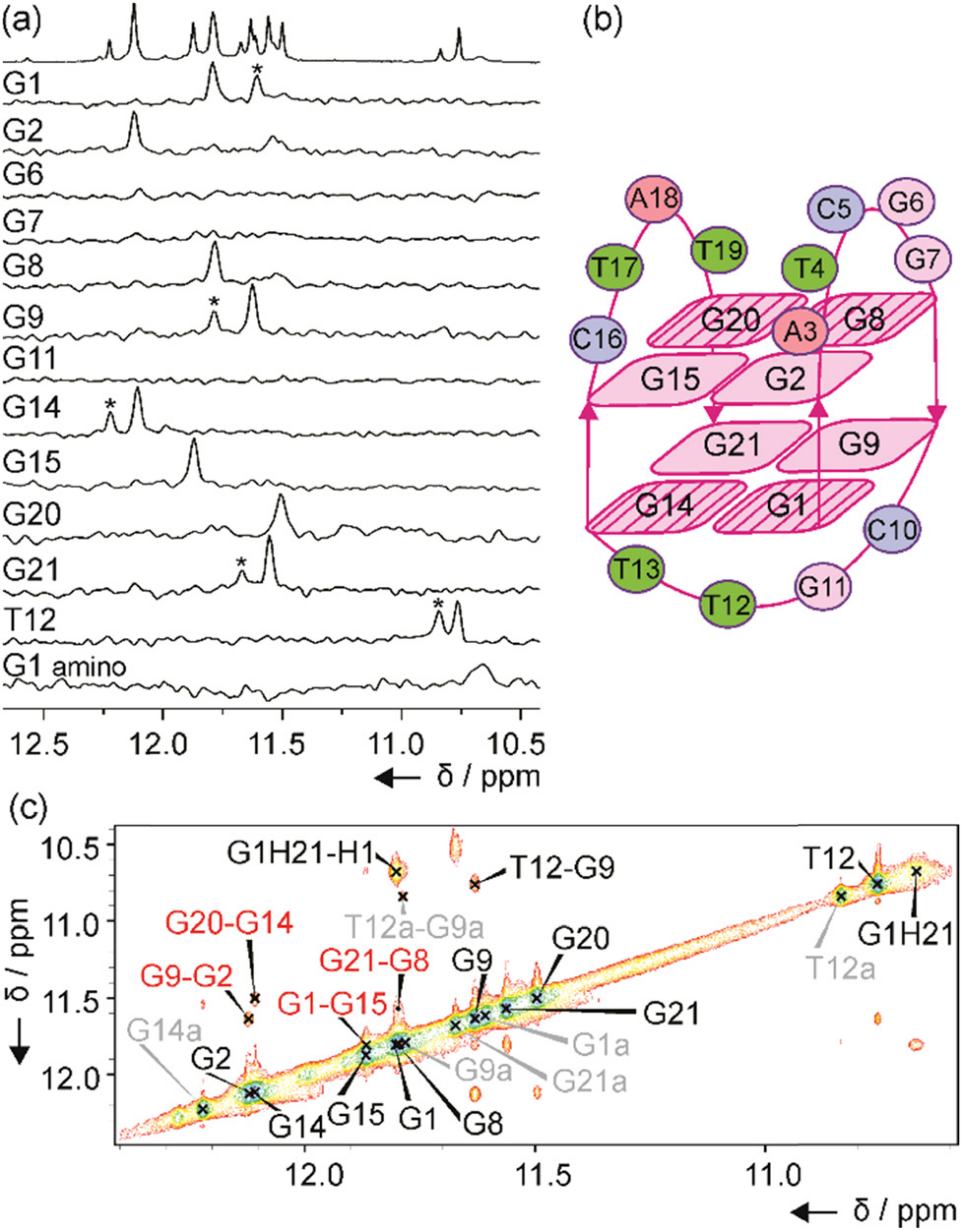
(a) Imino region of the ^1^H and 1D ^15^N-edited HSQC spectra of *mexC*. The HSQC spectra were acquired on partially residue-specifically ^15^N- and ^13^C-labeled oligonucleotides. Assignment of imino and amino resonances is indicated on the left side of each 1D HSQC spectrum. Asterisks mark the imino signals of the minor species. (b) Topology of G4 adopted by *mexC*. *Syn* guanines in G-quartets are striped. (c) Imino–imino region of the NOESY spectrum (*τ*_m_ 200 ms) of *mexC*. Inter-quartet H1–H1 NOE cross-peaks are marked with red. Signals assigned to the minor species are marked with grey and letter “a”. NMR spectra were recorded at 0.4 mM concentration of *mexC* per strand, 100 mM KCl, pH 7.4, 25 °C, on 600 and 800 MHz spectrometers.

The number of signals in the ^1^H NMR spectra of *murE*, *ftsB* and *mexC* oligonucleotides in the region between *δ* 11.0 and 12.5 ppm suggests the formation of more than one structure in solution. Well-resolved NMR spectra of *mexC* sequence allowed us to study its structural features, which was not possible in the case of *murE* and *ftsB* sequences due to the severe overlapping of the signals. Residue-specific, partial ^13^C,^15^N-labeling of guanine (10%) and thymine (8%) residues in the *mexC* oligonucleotide enabled the unambiguous assignment of H1 and H3 proton resonances of guanines and thymines, respectively. We identified eight guanines involved in G-quartets, while no signals were observed in imino region of the 1D ^15^N-edited HSQC spectra for G6, G7 and G11 residues suggesting that they are located in loops (Fig. 2a). It is interesting, that for G1, G9, G14, G21 and T12 residues additional imino signals with lower intensity were observed that belong to the minor structure of *mexC* (∼30%). Herein, we are focused on the structure of the major G4. The characteristic H1–H8 connectivities observed in the NOESY spectra allowed us to establish a topology for *mexC* G4, which involves two G-quartets with the following hydrogen-bond directionalities: G1 → G14 → G21 → G9 and G2 → G8 → G20 → G15 (Fig. 2b). The guanine residues of G1–G14–G21–G9 quartet exhibit a clockwise donor–acceptor hydrogen-bonding directionality, while those of G2–G8–G20–G15 quartet display an anti-clockwise directionality (Fig. S3, ESI†).

Orientation of G-quartets that are stacked one above the other is additionally supported by inter-quartet H1–H1 NOE cross-peaks of G1–G15, G2–G9, G8–G21 and G14–G20 residues (Fig. 2c). Four distinct and strong cross-peaks observed in the H8–H1′ region of the NOESY spectrum indicate that G1, G8, G14 and G20 residues predominantly adopt a s*yn* conformation along glycosidic torsion angles (Fig. S4a, ESI†). The characteristic rectangular pattern of NOE cross-peaks, H1′_(*n*)_–H8_(*n*+1)_–H1′_(*n*+1)_–H8_(*n*)_, for G1–G2, G8–G9, G14–G15 and G20–G21 sequential residues demonstrates that these connectivities are characteristic of 5′-*syn-anti*-3′ steps in an antiparallel topology (Fig. S4b, ESI†). This is in good agreement with the CD spectrum of *mexC* (Fig. 1a). Three loops, two edgewise and one diagonal, are arranged in an anticlockwise manner.^39^ The two edgewise loops containing five (A3–G7) and four (C16–T19) residues exhibited dynamic behavior. The observation of NOE cross-peak between A3 H2 and G2 H1 as well as A3 H2 and G15 H1 indicates stacking of A3 over the G2–G8–G20–G15 quartet (Fig. S3a, ESI†). In contrast, the diagonal loop consisting of C10–G11–T12–T13 is well-defined. Detailed information about this loop conformation can be obtained by focusing on the signals at *δ* 10.68 and 10.76 ppm assigned to G1 amino proton from G1–G14–G21–G9 quartet and T12 H3 from the diagonal loop, respectively (Fig. 2a). Detection of these signals at 25 °C is surprising due to the exchangeable nature of the amino and imino protons and indicates the tight packaging of residues in the diagonal loop under the G1–G14–G21–G9 quartet. The ^1^H NMR spectra of *mexC* analogs, where C10 and/or G11 were replaced with thymine residues, revealed that C10 is crucial in preventing fast exchange of T12 H3 with water protons (Fig. S5, ESI†). Furthermore, NOE cross-peaks observed between T12 H3 and C10 sugar protons indicating possible interactions between these two residues (Fig. S4c, ESI†). Peculiar conformation of C10 in the diagonal loop is reflected also in the upfield (*δ* 0.40 ppm) chemical shift of C10 H2′ compared to the averaged H2′ chemical shift range (Fig. S4c, ESI†).

### Small molecules stabilise *murE*, *ftsB* and *mexC* G4 DNA structures

**Fig. 3:**
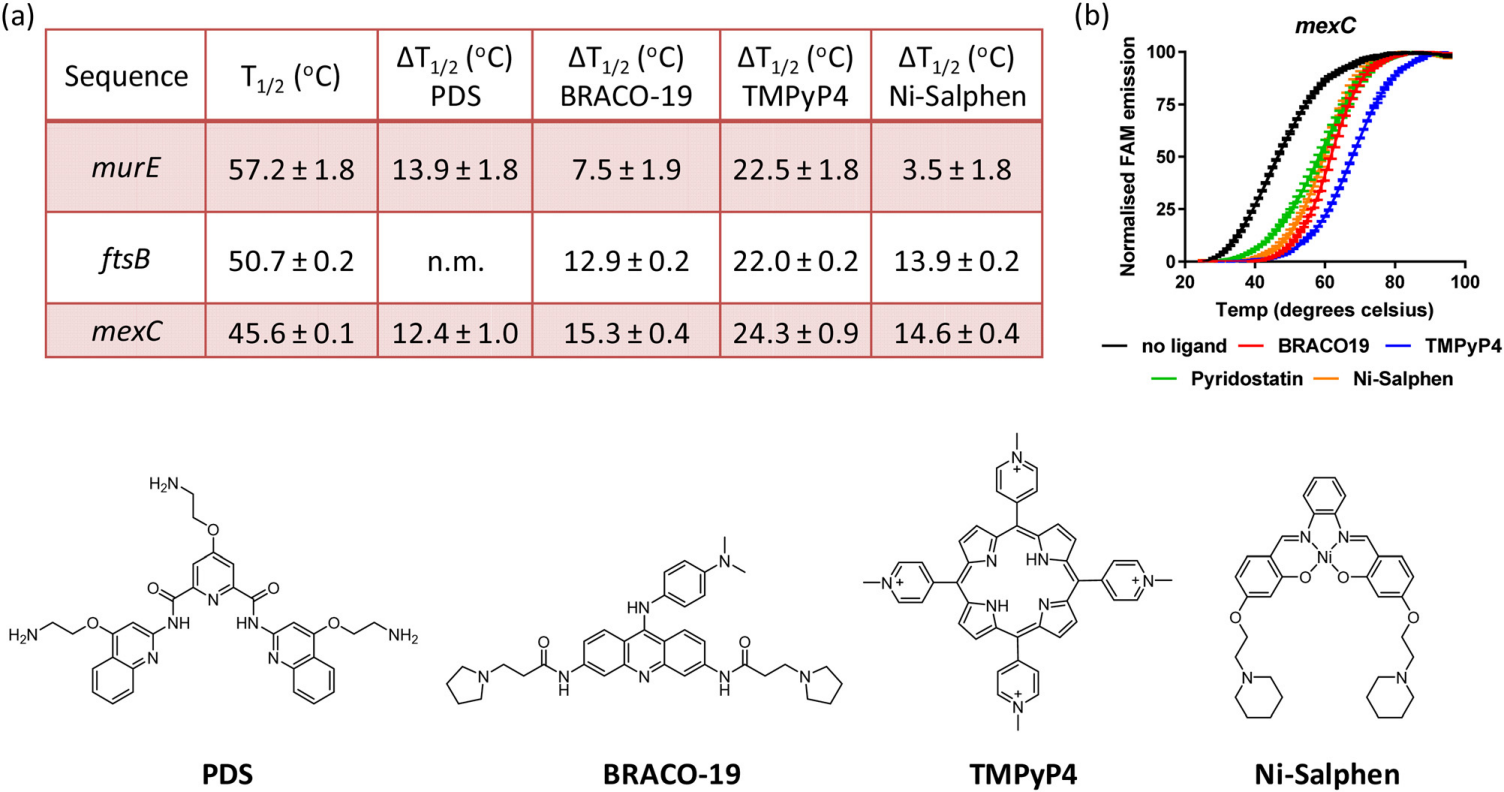
(a) FRET melting temperatures for FAM-TAMRA labelled *murE*, *ftsB* and *mexC* G4-DNA structures in the absence and presence of four different G-quadruplex binders; (b) example of FRET melting curves for *mexC* in the presence of the different compounds. Concentration of oligonucleotide = 0.4 μM; concentration of compound = 2 μM (n.m. = not measured).

Having established that sequences associated to the *murE, ftsB* and *mexC* genes fold into stable G4 structures, it was of interest to determine whether previously reported G4 DNA binders would stabilise these structures. Therefore, binding of BRACO19, PDS, TMPyP4 and Ni-salphen (see Fig. 3 for molecular structures) towards the three G4 structures was studied *via* FRET melting assays. The data shows that these four molecules stabilise the G4 structures with particularly high Δ*T*_m_ in the case of TMPyP4 (see Fig. 3 and Fig. S6, ESI†). Interestingly, Ni-salphen shows some selectivity towards the G4-forming sequences associated to *ftsB* and *mexC* over *murE*. In the case of the interaction of PDS with *ftsB*, the G4-DNA melting process did not follow the same pattern than for all other compounds and sequences (see Fig. S6, ESI†). Instead, a two-step melting process was observed, indicating a more complex interplay between the binding of PDS to different sites and/or topologies of *ftsB*-G4.

## Conclusion

There is significant interest in finding new targets for the development of antibacterial drugs, with particular emphasis in combating multidrug resistance. Previous studies on bacterial G4s are relatively scarce as compared to those on eukaryotes. With this in mind, we carried out bioinformatics studies to identify PQSs in the *P. aeruginosa* genome associated to genes relevant to antimicrobial resistance. CD spectroscopy, thermal difference UV/Vis spectroscopy and NMR spectroscopy showed that three of the ten sequences investigated *– i.e. murE*, *ftsB* and *mexC –* clearly fold into stable G-quadruplexes. It is worth highlighting that these sequences form two-tetrad G-quadruplexes, unlike most G4s reported to date. Detailed structural NMR studies for *mexC* confirmed the folding of this sequence into an antiparallel G4 structure with a diagonal loop. FRET melting assays for the three sequences showed that *murE* is the most thermally stable followed by *ftsB* and then *mexC*. Furthermore, with four known G4 binders, we show that the three structures can be further stabilized upon interaction with small molecules. Future studies should establish if formation of these G4s play regulatory roles in the expression of genes involved in crucial events such as drug efflux, cell division or cell wall biogenesis. If so, they could be new targets for antibacterial agents.

## Experimental details

### Circular dichroism (CD) measurements

DNA oligonucleotides (100 μM in milliQ water) were diluted to 5 μM in buffer containing 10 mM Tris-HCl pH 7.4 and 100 mM KCl to give a 700 μL final volume. The sample was annealed by heating to 95 °C for 5 minutes and then cooled to room temperature overnight. CD spectra were recorded on a JASCO-714 spectropolarimeter equipped with a MPTC-490S/15 multicell temperature unit using a 1 cm optical path and a reaction volume of 700 μL. Scans were performed at 20 °C over a wavelength range of 220–400 nm (3 accumulations) with a scanning speed of 100 nm min^−1^, 4 s response time, 1 nm data pitch and 1 nm bandwidth. The buffer spectrum was subtracted and plotted using GraphPad Prism 7.

### Thermal difference spectra

DNA oligonucleotides (100 μM in milliQ water) were diluted to 5 μM in buffer containing 10 mM LiCacodylate pH 7.4 and 100 mM KCl to give a 700 μL final volume. The sample was annealed by heating to 95 °C for 5 minutes and then cooled to room temperature overnight. Thermal denaturation was performed with a Cary 100 Bio UV-Visible Spectrophotometer and 1 cm path-length quartz cuvette. The UV spectra were measured over a 220–335 nm spectral range at a 1 nm data interval. Spectra were recorded at 20 °C, the sample was then heated to 90 °C and measurements repeated. The thermal difference spectra were obtained by subtracting the UV spectra at 20 °C from the corresponding UV spectra at 90 °C. Data was normalised and plotted *vs.* wavelength using GraphPad Prism 7.

### NMR experiments

NMR samples of *murE*, *ftsB* and *mexC* oligonucleotides were prepared by dissolving desalted oligonucleotides in 300 μL of 90%/10% mixture of H_2_O/^2^H_2_O, 10 mM lithium cacodylate buffer (pH 7.4) and in the presence of KCl with varying concentrations from 0 to 100 mM. The isotopically unlabeled and residue-specific partially ^15^N, ^13^C-labeled (10% guanine and 8% thymine residues) oligonucleotides were synthesized on K&A Laborgeraete GbR DNA/RNA Synthesizer H-8 using the standard phosphoramidite chemistry. Deprotection was done with the use of aqueous ammonia at 55 °C for 12 h. Samples were purified and desalted with the use of Amicon Ultra-15 Centrifugal Filter Units to give NMR samples with DNA concentration ∼0.4 mM per strand. NMR data were collected on Agilent (Varian) NMR System 600 and 800 MHz spectrometers equipped with ^1^H(^13^C/^15^N) ^13^C enhanced Cold Probe and HCN Cold Probe Gen, respectively. All NMR spectra were recorded at 25 °C. ^1^H and NOESY spectra were recorded with the use of the DPFGSE solvent suppression method. NOESY spectra were acquired at mixing times in the rage between 100 and 350 ms. ^1^H-^13^C HSQC and ^15^N-edited HSQC spectra were recorded on residue-specifically ^15^N, ^13^C-labelled oligonucleotides. NMR spectra were referenced to TMSpa (*δ* 0 ppm). NMR spectra were processed and analysed by using VNMRJ (Varian Inc.), the Sparky (UCSF) software and MestReNova (Mestrelab Research S.L.).

### FRET melting

FAM/TAMRA DNA oligonucleotides (100 μM in milliQ water) were diluted to 0.2 μM in buffer containing 10 mM Li cacodylate pH 7.4 and 100 mM KCl. The oligonucleotide solutions were annealed by heating them to 95 °C for 5 minutes and then cooled to room temperature over 3 hours. To each well of a clear 96-well plate was added, 40 μL of annealed DNA. The FAM fluorescence was monitored using an Agilent Stratagene Mx3005p thermocycler Agilent and recorded every 1 °C from 25 °C to 95 °C with a 1 °C min^−1^ melting slope. Data was collected *n* = 3 and analysed using GraphPad Prism 7.

### FRET Melting – Ligand stabilisation

FAM/TAMRA DNA oligonucleotides (100 μM in milliQ water) were diluted to 0.4 μM in buffer containing 10 mM Li cacodylate pH 7.4 and 100 mM KCl. The oligonucleotide samples were annealed by heating to 95 °C for 5 minutes and then cooled to room temperature over 3 hours. Solutions of the compounds (2 μM) were prepared in buffer containing 10 mM Li cacodylate pH 7.4 and 100 mM KCl. To each well of a clear 96-well plate was added: 20 μL of annealed oligonucleotide and 20 μL of compound solution. The mixture was incubated at room temperature for 15 minutes and then melted using an Agilent Stratagene Mx3005p thermocycler Agilent. FAM fluorescence was recorded every 1 °C from 25 °C to 95 °C with a 1 °C min^−1^ melting slope. Data was collected *n* = 3 and analysed using GraphPad Prism 7.

## Conflicts of interest

There are no conflicts to declare.

## Supplementary Material

CB-004-D2CB00205A-s001
