# Supplementary material for: Identification and characterisation of G-quadruplex DNA-forming sequences in the Pseudomonas aeruginosa genome
Source: RSC Chem Biol. 2022 Nov 15;4(1):94–100. doi: 10.1039/d2cb00205a (PMC9811510; doi:10.1039/d2cb00205a)
Supplement: CB-004-D2CB00205A-s001 [file CB-004-D2CB00205A-s001.pdf]

Electronic Supplementary Material (ESI) for RSC Chemical Biology.
This journal is © The Royal Society of Chemistry 2022

Identification and characterisation of G-quadruplex DNA-forming sequences in the *Pseudomonas aeruginosa* genome

Lindsay Evans, Anita Kotar, Martina Valentini, Alain Filoux, Shirin Jamshidi, Janez Plavec,

Khondaker Miraz Rahman*, Ramon Vilar*

Table S1: Genes that were studied to identify putative G-quadruplex forming sequences.

**gene name**	**Function**
ftsB	Cell division protein
mexR	multidrug resistance operon repressor MexR
mexA	Resistance-Nodulation-Cell Division (RND) multidrug efflux (*mexA*BoprM operon)
mexB	Resistance-Nodulation-Cell Division (RND) multidrug efflux (*mexA*BoprM operon)
oprM	Resistance-Nodulation-Cell Division (RND) multidrug efflux (*mexA*BoprM operon)
gacS	sensor/response regulator hybrid
oprD	basic amino acid, basic peptide and imipenem outer membrane porin OprD precursor
oprF	Major porin and structural outer membrane porin
mexT	transcriptional regulator
mexE	Resistance-Nodulation-Cell Division (RND) multidrug efflux (*mexE*FoprN operon)
mexF	Resistance-Nodulation-Cell Division (RND) multidrug efflux (*mexE*FoprN operon)
oprN	Resistance-Nodulation-Cell Division (RND) multidrug efflux (*mexE*FoprN operon)
czcC	Resistance-Nodulation-Cell Division (RND) multidrug efflux (*czcC*BA operon)
czcB	Resistance-Nodulation-Cell Division (RND) multidrug efflux (*czcC*BA operon)
czcA	Resistance-Nodulation-Cell Division (RND) multidrug efflux (*czcC*BA operon)
czcR	two-component system
czcS	two-component system
gacA	response regulator
ftsK	cell division protein
sagS	surface attachment and growth sensor hybrid
ladS	lost adherence sensor
ampR	transcriptional regulator
ampC	beta-lactamase precursor
mexC	Resistance-Nodulation-Cell Division (RND) multidrug efflux (*mexC*DoprJ operon)
mexD	Resistance-Nodulation-Cell Division (RND) multidrug efflux (*mexC*DoprJ operon)
oprJ	Resistance-Nodulation-Cell Division (RND) multidrug efflux (*mexC*DoprJ operon)
nfxB	transcriptional regulator
morA	mucin-adaptive protein, motility regulator

cbrA	two-component *cbrA*B system
cbrB	two-component *cbrA*B system
retS	regulator of exopolysaccharide and type III Secretion
parC	topoisomerase IV subunit A
Pile	type IV pili biogenesis
recQ	DNA helicase
pvrR	Two component *pvrR*S system
pvrS	Two component *pvrR*S system
ndvB	Biofilm resistance
bjfA	Biofilm regulator

Nucleotide sequence of the genes studied to identify putative G-quadruplex forming sequences. Start and stop codons are highlighted in red. Where found, G4 forming regions are highlighted in yellow. The nucleotide sequence of the upstream regulatory region (500 nucleotides upstream the start codon) and the sequence of the downstream region (250 nucleotides after the stop codon) were extracted and added using the KEGG database (https://www.genome.jp/kegg/).

> mexR(P52003)
TGAGGATGATGCCGTTCACCTGGGGACGCACCTCGGCGATGCGGAACGCATTGGTCCGGC
CCGGCAGCTCGGTATTCAGGGTCACCGTCTGCGCTTCCAGGGTCACGATCCCGACCTCCG
GCGTTTGCGCCGGCGGCGGCGCCTCGCTTTTTCCGCACCCGGAAAGGGCCGAAATCGCGA
CCAGCAGGGCCGGAACCAGTACACGCATGGCTGGCGTTCGTTGCATAGCGTTGTCCTCAT
GAGCGAAAGCGGCCGATGCAGCGTGAAGGCGCTGCACCGGCTCGAAGAAGGCAGGCAAAA
TACTTACATTCATAGGTGTTTGTAAACGTCCGAAAGCCTCGCGTGAAAACACCTGAAACG
TTTTAGCTCGATGGCCGGTTATCCACCCGGCGCCATGGCCCATATTCAGAACCTGAAACA
AGGTTGATAAGGTCAACTAAAATAAGCAAAATAGTTGACTGGATCAACCACATTTACATT
AGGTTTACTCGGCCAAACCA**ATG**AACTACCCCGTGAATCCCGACCTGATGCCCGCGCTGA
TGGCGGTCTTCCAGCATGTGCGGACGCGCATCCAGAGCGAGCTCGATTGCCAGCGACTCG
ACCTGACCCCGCCCGACGTCCATGTATTGAAGCTTATCGACGAACAACGCGGGCTGAACC
TGCAGGACCTGGGACGCCAGATGTGCCGCGACAAGGCACTGATCACCCGGAAGATCCGCG
AGCTGGAGGGAAGAAACCTGGTCCGCCGCGAGCGCAACCCCAGCGACCAGCGCAGCTTCC
AGCTCTTCCTCACCGACGAGGGGCTGGCCATCCACCAGCATGCGGAGGCCATCATGTCAC
GCGTGCATGACGAGTTGTTTGCCCCGCTCACCCCGGTGGAACAGGCCACCCTGGTGCATC
TCCTCGACCAGTGCCTGGCCGCGCAACCGCTTGAGGATATT**TAA**GAACATTCTTTTCGAA
GCACAATGAAAAACGCCGGCGATCGCCGGCGTTTTTCGTTTCAGGTATCCGCTTACTGGC
GAATGCCTTCGACGGAGAGCAGCAGCTCGACTTCCTGGGATGCCGGGCCGAGGTCCATCT
TGATGCCGAAGTCTTTCAGCTTCAGGGTGGCGCTGCCTTCGAAGCCGGCACGGTAGCCGC
CCCACGGGTCGTCGCCCTGGCCGATCAGCTTGGCCTTGATGGTGACCGGCTTGG

> mexA(P52477)
CCAGCTCGCGGATCTTCCGGGTGATCAGTGCCTTGTCGCGGCACATCTGGCGTCCCAGGT
CCTGCAGGTTCAGCCCGCGTTGTTCGTCGATAAGCTTCAATACATGGACGTCGGGCGGGG
TCAGGTCGAGTCGCTGGCAATCGAGCTCGCTCTGGATGCGCGTCCGCACATGCTGGAAGA
CCGCCATCAGCGCGGGCATCAGGTCGGGATTCACGGGGTAGTTCATTGGTTTGGCCGAGT
AAACCTAATGTAAATGTGGTTGATCCAGTCAACTATTTTGCTTATTTTAGTTGACCTTAT
CAACCTTGTTTCAGGTTCTGAATATGGGCCATGGCGCCGGGTGGATAACCGGCCATCGAG
CTAAAACGTTTCAGGTGTTTTCACGCGAGGCTTTCGGACGTTTACAAACACCTATGAATG
TAAGTATTTTGCCTGCCTTCTTCGAGCCGGTGCAGCGCCTTCACGCTGCATCGGCCGCTT
TCGCTCATGAGGACAACGCT**ATG**CAACGAACGCCAGCCATGCGTGTACTGGTTCCGGCCC
TGCTGGTCGCGATTTCGGCCCTTTCCGGGTGCGGAAAAAGCGAGGCGCCGCCGCCGGCGC
AAACGCCGGAGGTCGGGATCGTGACCCTGGAAGCGCAGACGGTGACCCTGAATACCGAGC
TGCCGGGCCGGACCAATGCGTTCCGCATCGCCGAGGTGCGTCCCCAGGTGAACGGCATCA
TCCTCAAGCGCCTGTTCAAGGAAGGCAGCGACGTCAAGGCCGGGCAGCAGCTCTACCAGA
TCGACCCCGCCACCTACGAGGCCGACTACCAGAGCGCCCAGGCCAACCTGGCTTCGACCC
AGGAACAGGCCCAGCGCTACAAGCTGCTGGTCGCCGACCAGGCCGTGAGCAAGCAGCAGT
ACGCCGACGCCAATGCCGCCTACCTGCAGTCCAAGGCGGCGGTGGAGCAGGCGCGGATCA
ACCTGCGCTACACCAAGGTGCTGTCGCCGATCTCCGGCCGCATCGGCCGTTCCGCGGTGA
CCGAAGGCGCCCTGGTGACCAACGGCCAGGCCAACGCGATGGCCACCGTGCAACAGCTCG
ACCCGATCTACGTCGACGTCACCCAGCCGTCCACCGCCCTGCTGCGCCTGCGCCGCGAAC
TGGCCAGCGGCCAGTTGGAGCGCGCCGGCGACAACGCGGCGAAGGTCTCCCTGAAGCTGG
AGGACGGTAGCCAATACCCGCTGGAAGGTCGCCTCGAATTCTCCGAGGTTTCCGTCGACG
AAGGCACCGGCTCGGTCACCATCCGCGCCGTGTTCCCCAACCCGAACAACGAGCTGCTGC
CCGGCATGTTCGTTCACGCGCAGTTGCAGGAAGGCGTCAAGCAGAAGGCCATCCTCGCTC
CGCAGCAAGGCGTGACCCGCGACCTCAAGGGCCAGGCTACCGCGCTGGTGGTGAACGCGC
AGAACAAGGTCGAGCTGCGGGTGATCAAGGCCGACCGGGTGATCGGCGACAAGTGGCTGG
TTACCGAAGGCCTGAACGCCGGCGACAAGATCATTACCGAAGGCCTGCAGTTCGTGCAGC
CGGGTGTCGAGGTGAAGACCGTGCCGGCGAAGAATGTCGCGTCCGCGCAGAAGGCCGACG
CCGCTCCGGCGAAAACCGACAGCAAGGGC**TGA**TCAAGGGGATTCGTAATGTCGAAGTTTT
TCATTGATAGGCCCATTTTCGCGTGGGTGATCGCCTTGGTGATCATGCTCGCGGGCGGCC

TGTCGATCCTCAGTCTGCCGGTCAACCAGTACCCGGCCATCGCCCCGCCGGCCATCGCCG
TGCAGGTGAGCTACCCGGGCGCCTCGGCCGAGACGGTGCAGGACACCGTGGTCCAGGTGA
TCGAGCAGCAGATGAACGGGATCGACAATCTGCGCTACATCT

> mexB(P52002)
GCGACAACGCGGCGAAGGTCTCCCTGAAGCTGGAGGACGGTAGCCAATACCCGCTGGAAG
GTCGCCTCGAATTCTCCGAGGTTTCCGTCGACGAAGGCACCGGCTCGGTCACCATCCGCG
CCGTGTTCCCCAACCCGAACAACGAGCTGCTGCCCGGCATGTTCGTTCACGCGCAGTTGC
AGGAAGGCGTCAAGCAGAAGGCCATCCTCGCTCCGCAGCAAGGCGTGACCCGCGACCTCA
AGGGCCAGGCTACCGCGCTGGTGGTGAACGCGCAGAACAAGGTCGAGCTGCGGGTGATCA
AGGCCGACCGGGTGATCGGCGACAAGTGGCTGGTTACCGAAGGCCTGAACGCCGGCGACA
AGATCATTACCGAAGGCCTGCAGTTCGTGCAGCCGGGTGTCGAGGTGAAGACCGTGCCGG
CGAAGAATGTCGCGTCCGCGCAGAAGGCCGACGCCGCTCCGGCGAAAACCGACAGCAAGG
GCTGATCAAGGGGATTCGTA**ATG**TCGAAGTTTTTCATTGATAGGCCCATTTTCGCGTGGG
TGATCGCCTTGGTGATCATGCTCGCGGGCGGCCTGTCGATCCTCAGTCTGCCGGTCAACC
AGTACCCGGCCATCGCCCCGCCGGCCATCGCCGTGCAGGTGAGCTACCCGGGCGCCTCGG
CCGAGACGGTGCAGGACACCGTGGTCCAGGTGATCGAGCAGCAGATGAACGGGATCGACA
ATCTGCGCTACATCTCCTCGGAGAGTAACTCCGACGGCAGCATGACCATCACCGTGACCT
TCGAACAGGGCACCGACCCCGACATCGCCCAGGTCCAGGTGCAGAACAAGCTGCAACTGG
CCACCCCGCTACTGCCGCAGGAAGTGCAGCGCCAGGGGATCCGGGTGACCAAGGCGGTGA
AGAACTTCCTCATGGTGGTCGGTGTGGTTTCCACCGACGGCAGCATGACCAAGGAAGACC
TGTCGAACTACATCGTTTCCAACATCCAGGACCCACTCTCGCGGACCAAGGGCGTCGGTG
ACTTCCAGGTGTTCGGCTCGCAGTACTCGATGCGCATCTGGCTCGACCCGGCCAAGCTGA
ACAGCTACCAGCTGACCCCCGGCGACGTGAGCAGCGCGATCCAGGCGCAGAACGTGCAGA
TTTCCTCCGGCCAGCTCGGCGGCTTGCCGGCGGTCAAGGGCCAGCAGCTCAACGCCACCA
TCATCGGCAAGACCCGCCTGCAGACCGCGGAGCAATTCGAGAACATCCTGCTCAAGGTCA
ATCCCGACGGTTCCCAGGTGCGCCTGAAGGACGTCGCCGATGTAGGCCTGGGCGGCCAGG
ACTACAGCATCAACGCGCAGTTCAACGGCAGCCCGGCGTCCGGTATCGCGATCAAGCTGG
CCACCGGCGCCAACGCGCTGGATACCGCCAAGGCGATCCGCCAGACCATCGCCAACCTGG
AACCGTTCATGCCGCAGGGCATGAAGGTGGTCTACCCGTACGACACCACCCCGGTGGTCT
CGGCCTCGATCCATGAGGTAGTGAAGACCCTCGGCGAGGCGATCCTCCTCGTGTTCCTGG
TGATGTACCTGTTCCTGCAGAACTTCCGCGCCACGCTGATCCCGACCATCGCCGTACCGG
TGGTGCTGCTGGGGACCTTCGGCGTGCTCGCCGCGTTCGGCTTCTCGATCAACACCCTGA
CCATGTTCGGCATGGTGCTGGCCATCGGCTTGCTGGTGGACGACGCCATCGTGGTGGTGG
AGAACGTCGAGCGGGTGATGGCCGAGGAAGGCCTGTCGCCAAGGGAGGCGGCGCGCAAGT
CCATGGGCCAGATCCAGGGCGCGCTGGTCGGTATCGCCATGGTGCTCTCGGCGGTATTCC
TGCCGATGGCGTTCTTCGGCGGCTCCACCGGGGTGATCTACCGGCAGTTCTCCATCACCA
TCGTGTCGGCCATGGCCCTCTCGGTGATCGTGGCGCTGATCCTCACCCCGGCGCTCTGCG
CGACCATGCTCAAGCCGATCGAGAAAGGCGACCATGGCGAGCACAAGGGCGGCTTCTTCG
GCTGGTTCAACCGGATGTTCCTTTCCACCACCCACGGCTACGAGCGGGGCGTGGCGTCGA
TCCTCAAGCATCGCGCGCCGTACCTGCTGATCTACGTGGTGATCGTGGCCGGGATGATCT
GGATGTTCACCCGCATTCCCACCGCGTTCCTCCCCGACGAGGACCAGGGCGTACTGTTCG
CCCAGGTACAGACCCCGCCGGGCTCCAGTGCCGAGCGTACCCAGGTGGTGGTGGACTCGA
TGCGCGAATACCTGCTGGAGAAGGAAAGCTCTTCGGTCAGCTCGGTGTTCACCGTGACCG
GCTTCAACTTCGCCGGCCGCGGCCAGAGTTCGGGCATGGCGTTCATCATGCTCAAGCCCT
GGGAAGAGCGTCCCGGTGGCGAGAACAGCGTGTTCGAACTGGCCAAGCGCGCGCAGATGC
ACTTCTTCAGCTTCAAGGACGCGATGGTGTTCGCCTTCGCGCCGCCGTCGGTACTGGAAC
TGGGTAACGCCACCGGCTTCGACCTGTTCCTCCAGGACCAGGCGGGTGTCGGCCACGAAG
TCCTGCTCCAGGCGCGCAACAAGTTCCTCATGCTCGCCGCGCAGAACCCGGCGCTGCAAC
GCGTGCGCCCCAACGGCATGAGCGACGAACCGCAGTACAAGCTGGAGATCGACGACGAGA
AGGCCAGCGCCCTCGGCGTGTCCCTTGCCGACATCAACAGCACCGTGTCCATCGCCTGGG
GTTCCAGCTACGTCAACGATTTCATCGACCGTGGCCGGGTCAAGCGGGTCTACCTGCAGG
GCAGGCCGGACGCGCGGATGAACCCGGACGACCTGAGCAAGTGGTACGTGCGCAACGACA
AGGGCGAGATGGTGCCGTTCAACGCCTTCGCCACCGGCAAGTGGGAATACGGTTCGCCGA
AGCTGGAGCGCTACAATGGCGTGCCGGCGATGGAGATCCTCGGCGAGCCGGCGCCCGGCC
TGAGTTCCGGTGACGCCATGGCGGCGGTCGAGGAGATCGTCAAGCAATTGCCGAAAGGCG
TTGGCTACTCCTGGACCGGCCTGTCCTACGAGGAGCGCTTGTCCGGCTCGCAGGCGCCGG
CGCTGTATGCGCTGTCGCTGCTGGTGGTGTTCCTCTGCCTGGCGGCCCTGTACGAAAGCT
GGTCGATTCCGTTCTCGGTGATGCTGGTGGTGCCGTTGGGCGTGATCGGTGCGCTGCTGG
CGACGTCCATGCGCGGCCTGTCCAACGACGTGTTCTTCCAGGTGGGCCTGTTGACGACCA
TCGGCCTGTCGGCGAAGAACGCCATTCTCATCGTGGAGTTCGCCAAGGAGCTGCACGAGC

AGGGCAAGGGCATCGTCGAGGCGGCCATCGAAGCCTGCCGCATGCGTCTGCGGCCGATCG
TGATGACCTCCCTGGCGTTCATCCTCGGCGTGGTCCCGCTGGCGATCTCCACCGGCGCCG
GCTCGGGCAGCCAGCATGCGATCGGTACCGGCGTGATCGGCGGCATGGTCACTGCGACCG
TCCTGGCGATCTTCTGGGTACCGCTGTTCTACGTGGCGGTCAGCACGCTGTTCAAGGACG
AGGCGTCCAAGCAGCAGGCGTCCGTCGAAAAGGGGCAA**TGA**TATGAAACGGTCCTTCCTT
TCCCTGGCGGTAGCCGCTGTCGTTCTGTCCGGCTGCTCGCTGATCCCCGACTACCAGCGC
CCCGAGGCGCCGGTAGCCGCGGCCTACCCGCAAGGGCAGGCCTACGGGCAGAACACCGGC
GCGGCGGCCGTTCCGGCCGCCGACATCGGCTGGCGCGAGTTCTTCCGCGACCCGCAGTTG
CAGCAACTGATCGGCGTGGCGCTGGAAAACAACCGCGACCTGCGGGTCGCC

> oprM (Q51487)
GGTGGTGTTCCTCTGCCTGGCGGCCCTGTACGAAAGCTGGTCGATTCCGTTCTCGGTGAT
GCTGGTGGTGCCGTTGGGCGTGATCGGTGCGCTGCTGGCGACGTCCATGCGCGGCCTGTC
CAACGACGTGTTCTTCCAGGTGGGCCTGTTGACGACCATCGGCCTGTCGGCGAAGAACGC
CATTCTCATCGTGGAGTTCGCCAAGGAGCTGCACGAGCAGGGCAAGGGCATCGTCGAGGC
GGCCATCGAAGCCTGCCGCATGCGTCTGCGGCCGATCGTGATGACCTCCCTGGCGTTCAT
CCTCGGCGTGGTCCCGCTGGCGATCTCCACCGGCGCCGGCTCGGGCAGCCAGCATGCGAT
CGGTACCGGCGTGATCGGCGGCATGGTCACTGCGACCGTCCTGGCGATCTTCTGGGTACC
GCTGTTCTACGTGGCGGTCAGCACGCTGTTCAAGGACGAGGCGTCCAAGCAGCAGGCGTC
CGTCGAAAAGGGGCAATGAT**ATG**AAACGGTCCTTCCTTTCCCTGGCGGTAGCCGCTGTCG
TTCTGTCCGGCTGCTCGCTGATCCCCGACTACCAGCGCCCCGAGGCGCCGGTAGCCGCGG
CCTACCCGCAAGGGCAGGCCTACGGGCAGAACACCGGCGCGGCGGCCGTTCCGGCCGCCG
ACATCGGCTGGCGCGAGTTCTTCCGCGACCCGCAGTTGCAGCAACTGATCGGCGTGGCGC
TGGAAAACAACCGCGACCTGCGGGTCGCCGCGCTGAACGTCGAGGCCTTCCGGGCGCAGT
ACCGCATCCAGCGGGCCGACCTGTTCCCGCGGATCGGCGTGGACGGTAGCGGCACCCGCC
AGCGTTTGCCGGGCGACCTGTCGACCACCGGCAGTCCGGCGATTTCCAGCCAGTACGGGG
TGACCCTGGGCACTACCGCCTGGGAACTCGATCTCTTCGGCCGCCTGCGCAGCCTGCGCG
ACCAGGCCCTGGAGCAGTACCTGGCGACCGAACAGGCGCAGCGCAGCGCGCAGACCACCC
TGGTGGCCAGCGTGGCGACCGCCTACCTGACGCTGAAGGCCGACCAGGCGCAGTTGCAGC
TGACCAAGGACACCCTGGGCACCTACCAGAAGAGTTTCGACCTGACCCAGCGCAGCTACG
ACGTCGGCGTCGCCTCCGCGCTCGACCTGCGCCAGGCGCAGACCGCCGTGGAAGGCGCCC
GCGCGACCCTGGCGCAGTACACCCGCCTGGTAGCCCAGGACCAGAATGCGCTGGTCCTGC
TGCTGGGCTCCGGGATCCCGGCGAACCTGCCGCAAGGCCTGGGCCTGGACCAGACCCTGC
TGACCGAAGTGCCGGCGGGTCTGCCGTCGGACCTGCTGCAACGGCGCCCGGACATCCTCG
AGGCCGAGCACCAGCTCATGGCTGCCAACGCCAGCATCGGCGCCGCGCGCGCGGCGTTCT
TCCCGAGCATCAGCCTGACCGCCAACGCCGGCACCATGAGCCGCCAACTGTCCGGCCTGT
TCGACGCCGGTTCGGGTTCCTGGTTGTTCCAGCCGTCGATCAACCTGCCGATCTTCACCG
CCGGCAGCCTGCGTGCCAGCCTGGACTACGCGAAGATCCAGAAGGACATCAACGTCGCGC
AGTACGAGAAGGCGATCCAGACGGCGTTCCAGGAAGTCGCCGACGGCCTGGCCGCGCGCG
GTACCTTCACCGAGCAGTTGCAGGCGCAGCGCGATCTGGTCAAGGCCAGCGACGAGTACT
ACCAGCTCGCCGACAAGCGCTATCGCACGGGGGTGGACAACTACCTGACCCTGCTCGACG
CGCAACGCTCGCTGTTCACCGCGCAGCAGCAACTGATCACCGACCGCCTCAATCAGCTGA
CCAGCGAGGTCAACCTGTACAAGGCCCTCGGCGGCGGCTGGAACCAGCAGACCGTGACCC
AGCAGCAGACCGCGAAGAAGGAAGATCCCCAGGCT**TGA**TCGCCTTCCGCGCCATGCAAGA
ACGCCGACCCTAGGGTCGGCGTTTTTTTATCCTCGCGGAAGTCGGCGGGTCCGCGTCACG
GACCCGCTCGGCCTTAGCGGTTGCGGGTCAGCAGTGCGGGGCGCTCGCCCTTGCGCCGCG
GTTCCAGCTCGTCGAGCTGCTCGGCGGTGGGGAAGCGATCCATGCGCACCAGGTCGCGCT
TGTTGATCACCACCGGCTGGCGGCTCGGCTGCTTGCCGGTGCCATACT

> gacS (G3XD98)
ATAATCGTCGGCGCCCTGGCTGAACCCGGCGATGCGGTCCTGCTCGGCGCCCAGGGCTGA
CATCAGGATCACCGGCAGCGCCTGCTCCTGGCGGAGTTGGCGAAGAATCTCGAGGCCATC
GCGCCCGGGCAGCATGATGTCCATCAGGATCAGGTCATAGCCGCCGCTGCGCGCAGCCGC
CAACCCCTCTTCCCCGTCTCGGCACCAGGTCACCTCGAAACCGCTGCCATTGAGGTGCTG
GAACAGATGTGCGCCCAGTGTCGGATCGTCCTCGATGGCCAGCAGTCGGGGGTCGGGCGT
CAGGGATGGGAACATGGCAGCCAAATAAGATTGATTAGCAATCGTGCCAGTATTCACGAT
CTTTCCGCTTCGTCGCAAGCCGAATCCGCCGGGCTGCGCACATCGGTGCAGATTCCCCTT
CCGCGACCCGCTAAACTGCGCGGGGTAAACGTATGGCCGGGGAGCGTCGGCCATCCCGAT
CTGGCTCGACGGAGAGACGT**GTG**TTCAAGGATCTCGGCATCAAGGGGCGCGTACTGCTGC
TCACCCTGCTCCCCACCAGCCTGCTGGCGATGGTGCTTGGCGGTTACTTCACCTGGGTCC
AGCTGTCCGACATGCGCGCCCAGTTGATCGAGCGCGGGCAACTGATCGCCGAACAACTGG

CGCCGCTGGCCGCCACCGCGCTGGCGCGAAAGGATACCGCCGTGCTCAACCGCATCGCCA
ACGAGGCGCTGGACCAACCGGACGTGCGCGCGGTGACCTTCCTCGACGCCCGCCAGGAAC
GCCTCGCCCATGCCGGGCCAAGCATGCTCACCGTCGCCCCGGCCGGCGACGCCAGCCATT
TGAGCATGTCCACCGAACTGGACACCACGCACTTCCTGCTACCGGTTCTTGGCCGCCACC
ACAGCCTGTCCGGCGCCACCGAGCCTGACGACGAGCGCGTACTCGGCTGGGTCGAACTGG
AACTGTCGCACCACGGGACTCTGCTGCGCGGATATCGCAGCCTGTTCACCAGCCTCTTGC
TGATCGCCGCCGGCCTCGGCGTCACCGCCCTCCTCGCCCTGCGCATGAGCCGCGCGATCA
ACGCGCCGCTGGAACTGATCAGCCAGGGCGTCGCCCAGCTCAAGGAAGGCCGCATGGAAA
CCCGCCTGCCACCGATGGGCAGCAACGAGCTGGACGAACTGGCCTCTGGCATCAACCGCA
TGGCGGAAACGCTGCAGAGCGCCCAGGAGGAAATGCAGCACAACATCGACCAGGCCACCG
AGGACGTACGGCAGAACCTGGAAACCATCGAGATCCAGAACATCGAGCTGGACCTGGCGC
GCAAGGAGGCCCTGGAGGCGAGCAGGATCAAGTCCGAGTTCCTCGCCAACATGAGCCACG
AGATCCGCACCCCGCTCAACGGCATCCTCGGTTTCACCAACCTGCTGCAGAAGAGCGAGC
TCAGCCCGCGCCAGCAGGACTACCTCACGACCATCCAGAAATCGGCGGAAAGCCTGCTGG
GGATCATCAACGAGATCCTCGATTTCTCGAAGATCGAGGCCGGCAAGCTGGTTCTGGAAA
ACCTCCCTTTCAATCTCCGCGACCTGATCCAGGACGCCCTGACCATGCTGGCTCCGGCCG
CCCACGAGAAGCAACTGGAACTGGTCAGCCTGGTCTACCGGGATACCCCGATCCAATTGC
AGGGCGACCCGCAGCGGCTGAAGCAGATCCTCACCAACCTGGTCGGCAACGCCATCAAGT
TCACCCAGGGCGGCACCGTCGCCGTACGCGCCATGCTCGAGGACGAAAGCGACGACCGCG
CGCAGCTGCGGATCAGCGTCCAGGACACCGGTATCGGCCTCTCCGAGGAAGACCAGCAAG
CCTTGTTCAAGGCCTTCAGCCAGGCCGACAACTCACTGTCGCGGCAAGCCGGTGGCACCG
GCCTGGGCCTGGTGATCTCCAAGCGCCTGATTGAGCAGATGGGCGGCGAGATCGGCGTCG
ACAGTACGCCTGGGGAAGGCGCCGAGTTCTGGATCAGCCTGAGTCTGCCGAAAAGTCGCG
ACGACAACGAGGAGCCGGGCGCCTCCTGGGCCGCGGGCCAACGCGTGGCGCTGCTCGAAC
CGCAGGAACTGACGCGCCGCTCGCTGCACCACCAGCTCACCGACTTCGGCCTGGAAGTGA
GCGAATTCGCCGACCTCGACAGCCTCCAGGAAAGCCTGCGCAACCCGCCGCCCGGCCAGT
TGCCGATCAGCCTGGCGGTGCTCGGCGTCTCGGCCGCGATCCATCCGCCGGAAGAGCTGA
GCCAGTCGTTCTGGGAATTCGAACGGCTCGGCTGCAAGACCCTGGTGCTCTGCCCGACCA
CCGAGCAGGCGCAATACCACGCGACCCTGCCCGACGAACAGGTCGAGGCCAAGCCCGCCT
GCACCCGCAAGCTGCAACGCAAGCTGCAGGAGTTGCTTCAAGTCCGCCCGACGCGCAGCG
ACAAGCCCCACGCCATGGTTTCCGGACGGCCGCCACGGCTGCTATGCGTCGACGACAACC
CGGCCAACCTGCTGCTGGTGCAGACCCTGCTCAGCGACCTCGGCGCCCAGGTCACCGCGG
TGGACAGCGGCTACGCGGCCCTCGAGGTAGTGCAGCGCGAGCGCTTCGACCTGGTCTTCA
TGGACGTGCAGATGCCCGGCATGGACGGCCGCCAGGCCACCGAGGCGATCCGCCGCTGGG
AGGCCGAGCGGGAAGTCAGCCCGGTGCCGGTGATCGCGCTCACCGCACATGCGCTTTCCA
ACGAGAAGCGCGCATTGCTGCAGGCCGGCATGGACGACTACCTGACCAAGCCGATCGACG
AGCAGCAATTGGCCCAGGTAGTGCTGAAGTGGACCGGACTGAGCCTGGGCCAGTCGCTGG
CCAGCATGAGCCGTGCGCCGCAGCTCGGCCAGTTGAGCGTGCTCGACCCCGAGGAAGGGC
TGCGCCTGGCCGCCGGCAAGGCCGACCTCGCCGCCGACATGCTGGCGATGCTGCTGGCCT
CGCTGGCGGCGGACCGCCAGGCGATTCGCCAGGCCCGCGACAACGACGACCGCACCGCTT
TGCTCGAGAGGGTCCACCGGCTGCATGGCGCCACCCGCTACTGTGGCGTGCCGCAGTTGC
GCGCGGCCTGCCAGACCAGCGAAACCCTGCTCAAGCAGAACGATCCGGCGGCGGCCGCGG
CCCTGGACGAGCTGGACAAGGCCATCGAGGCCCTGGCCGACACTGCCTCGGCCACCACCC
ACCTGTCCTCCACCAGCCTCGACTCCAGCGAACTC**TGA**CCATGCGCATCCTGTTCTTCAG
CAGCCAGGCCTACGACAGCGAGAGCTTCCAGGCCAGCAACCACCGGCACGGCTTCGAACT
GCACTTCCAGCAGGCCCACCTGCAGGCGGACACGGCGGTCCTCGCCCAGGGCTTCGAAGT
AGTCTGCGCCTTCGTCAACGACGACCTCTCGCGGCCGGTGCTGGAACGCCTGGCGGCCGG
CGGCACGCGCCTGGTCGCCCTGCGCTCGGCCGGCTACAACCACGTCGA

> oprD (P32722)
CAAACGCATTCGCCACAGACAACTCGATGGCAACCAACCCTTGAAGCAGACGGATTACAA
TCAGGTTTCAAAGCATAATTCGTTTGCTTTCAAACAGAATAGCCTCGCTCTCGAAGAGAC
CAACTGGAATACATAGGCGAAGCCATTTTCCAATTTGTGCACGGAGTTTGCTTATACCTC
TTTCATCACAGTAAGAGGGGCCGTACGGAACATGACATTTTTATTACAAGGCCCCGCCAA
TCGGGAAAAGCGACTTGAGAAGCGACCTCAACAAGAGTGACCAACCCCGCGACATACGTC
ATTTTTTCAACTGCGCACCTACGCAGATGCGACATGCGTCATGCAATTTTGCGACAGCAC
GGTAAAGAATCCGTCGCTTCGGAACCTCAACTATCGCCAAGAAACACTGCGTGCTATAAG
TTAGCGCCGACAAGAAGAACTAGCCGTCACTGCGGCACTGTGATGGCAGAGATAATTTCA
AAACCAAAGGAGCAATCACA**ATG**AAAGTGATGAAGTGGAGCGCCATTGCACTGGCGGTTT
CCGCAGGTAGCACTCAGTTCGCCGTGGCCGACGCATTCGTCAGCGATCAGGCCGAAGCGA
AGGGGTTCATCGAAGACAGCAGCCTCGACCTGCTGCTCCGCAACTACTATTTCAACCGTG

ACGGCAAGAGCGGCAGCGGGGACCGCGTCGACTGGACCCAAGGCTTCCTCACCACCTATG
AATCCGGCTTCACCCAAGGCACTGTGGGCTTCGGCGTCGATGCCTTCGGCTACCTGGGCC
TGAAGCTCGACGGCACCTCCGACAAGACCGGCACCGGCAACCTGCCGGTGATGAACGACG
GCAAGCCGCGCGATGACTACAGCCGCGCCGGCGGCGCCGTGAAGGTGCGCATCTCCAAGA
CCATGCTGAAGTGGGGCGAGATGCAACCGACCGCCCCGGTCTTCGCCGCTGGCGGCAGCC
GCCTGTTCCCGCAGACCGCGACCGGCTTCCAGCTGCAGAGCAGCGAATTCGAAGGGCTCG
ACCTCGAGGCAGGCCACTTCACCGAGGGCAAGGAGCCGACCACCGTCAAATCGCGTGGCG
AACTCTATGCCACCTACGCAGGCGAGACCGCCAAGAGCGCCGATTTCATTGGGGGCCGCT
ACGCAATCACCGATAACCTCAGCGCCTCCCTGTACGGCGCCGAACTCGAAGACATCTATC
GCCAGTATTACCTGAACAGCAACTACACCATCCCACTGGCATCCGACCAATCGCTGGGCT
TCGATTTCAACATCTACCGCACAAACGATGAAGGCAAGGCCAAGGCCGGCGACATCAGCA
ACACCACTTGGTCCCTGGCGGCAGCCTACACTCTGGATGCGCACACTTTCACCTTGGCCT
ACCAGAAGGTCCATGGCGATCAGCCGTTTGATTATATCGGCTTCGGCCGCAACGGCTCTG
GCGCAGGTGGCGACTCGATTTTCCTCGCCAACTCTGTCCAGTACTCCGACTTCAACGGCC
CTGGCGAGAAATCCTGGCAGGCTCGCTACGACCTGAACCTAGCCTCCTATGGCGTTCCCG
GCCTGACTTTCATGGTCCGCTATATCAATGGCAAGGACATCGATGGCACCAAGATGTCTG
ACAACAACGTCGGCTATAAGAACTACGGCTACGGCGAGGATGGCAAGCACCACGAAACCA
ACCTCGAAGCCAAGTACGTGGTCCAGTCCGGTCCGGCCAAGGACCTGTCGTTCCGCATCC
GCCAGGCCTGGCACCGTGCCAACGCCGACCAGGGCGAAGGCGACCAGAACGAGTTCCGCC
TGATCGTCGACTATCCGCTGTCGATCCTG**TAA**TCGACCGACAGGCAACGAAAAAACCCGG
CATCGCCGGGTTTTTTCTTCTTGGCGGCAACGCGCCTATAAAGGAAGGGCGTAGGTACCG
GTGACATGGGCCACCAGGTCTTCCTCATTACTCTGCGAAAACACCCCGACCTCGCAGACG
ACCTGGCGCCGCCCCAGCTTGAGGATGCTCGCCTCGGCCAACAGGTCCTCGGGCCTGGGC
TTGCTGAGGAAGTTGATGTTCAGGTTCGAGGTCACGGCCATC

> murE (Q59650)
ACCGCGATCCAGTTGGCGCATGCCTATGCGGCCCTGGCCAACGACGGCAAGAGCGTGCCG
CTGAGCATGACCCGAGTCGACCGCGTGCCGGATGGTGTGCAGGTGATCTCGCCTGAAGTG
GCTTCCACCGTGCAGGGCATGCTGCAACAAGTGGTCGAGGCCCAGGGCGGGGTGTTCCGC
GCCCAGGTGCCGGGTTACCACGCCGCCGGCAAGAGCGGGACCGCGCGCAAGGTCTCGGTC
GGCACCAAGGGCTACCGGGAAAACGCCTATCGCTCGCTGTTCGCCGGTTTCGCCCCGGCC
ACCGATCCGCGCATCGCGATGGTCGTGGTGATCGACGAGCCGAGCAAGGCGGGCTACTTC
GGCGGCCTGGTGTCGGCGCCGGTGTTCAGTAAGGTCATGGCTGGCGCGCTGCGCCTGATG
AACGTGCCGCCGGATAACCTGCCGACGGCCACCGAACAGCAGCAGGTCAATGCTGCGCCC
GCAAAAGGAGGGCGTGGCTG**ATG**CCTATGAGCCTGAACCAACTGTTTCCCCAGGCCGAGC
GCGATCTGCTGATCCGCGAGCTGACCCTGGATAGCCGCGGCGTTCGTCCGGGCGACCTGT
TCCTGGCGGTGCCGGGCGGGCGCCAGGATGGTCGTGCGCACATCGCCGATGCCCTGGCCA
AGGGCGCGGCTGCCGTGGCTTACGAGGCGGAAGGCGCCGGAGAGTTGCCGCCCAGCGATG
CGCCGCTGATCGCGGTGAAGGGGCTGGCCGCGCAACTGTCGGCGGTCGCCGGGCGTTTCT
ACGGCGAGCCGAGCCGCGGGCTGGACCTGATCGGCGTCACCGGCACCAACGGCAAGACCA
GCGTCAGCCAACTGGTGGCCCAGGCCCTGGATCTGCTCGGCGAGCGCTGCGGCATCGTCG
GCACCCTCGGCACCGGTTTCTACGGCGCCCTGGAGAGCGGCCGGCACACCACGCCGGACC
CGCTCGCGGTGCAGGCCACGCTGGCCACGCTGAAGCAGGCCGGCGCCCGCGCGGTAGCGA
TGGAAGTGTCTTCCCACGGCCTCGACCAGGGCCGCGTGGCGGCGCTCGGTTTCGATATCG
CGGTGTTCACCAATCTGTCCCGCGACCACCTCGACTATCACGGTTCGATGGAAGCCTATG
CCGCCGCCAAGGCCAAGCTGTTCGCCTGGCCGGGCCTGCGCTGCCGGGTGATCAACCTGG
ACGACGATTTCGGCCGTCGACTGGCCGGCGAGGAGCAGGACTCGGAGCTGATCACCTACA
GCCTCACCGACAGCTCGGCGTTCCTCTATTGCCGCGAAGCGCGCTTCGGCGACGCCGGCA
TCGAGGCGGCGCTGGTCACTCCGCACGGCGAGGGCCTGCTGCGCAGCCCGTTGCTCGGCC
GCTTCAACCTGAGCAACCTGCTGGCGGCGGTCGGTGCGTTGCTTGGCCTGGGTTATCCCC
TGGGCGATATCCTCCGCACTTTGCCGCAACTGCAGGGGCCGGTCGGCCGCATGCAGCGCC
TGGGAGGCGGCGACAAGCCGCTGGTGGTGGTGGACTACGCGCATACTCCCGACGCCCTGG
AAAAAGTCCTGGAGGCCCTGCGTCCGCACGCGGCCGCGCGCCTGCTGTGCCTGTTCGGCT
GCGGTGGCGATCGCGATGCCGGCAAGCGTCCGCTGATGGCTGCGATCGCCGAACGCCTGG
CGGATGAGGTGCTGGTCACCGACGACAACCCGCGCACCGAGGCCAGTGCGGCGATCATCG
CCGATATCCGCAAAGGCTTCGCTGCCGCTGACAAGGTTACCTTCCTGCCGTCGCGCGGTG
AGGCGATCGCCCATCTGATCGCTTCCGCTGCGGTGGATGACGTGGTGCTCCTGGCCGGCA
AGGGTCACGAGGATTATCAGGAGATCGACGGCGTACGCCATCCGTTCTCCGACATCGAGC
AGGCCGAGCGCGCCCTGGCCGCCTGGGAGGTGCCGCATGCT**TGA**GCCTCTTCGCCTCAGC
CAGTTGACGGTCGCGCTGGACGCCCGCCTGATCGGCGAGGACGCCGTCTTTTCGGCGGTT
TCCACCGACAGTCGCGCCATCGGGCCCGGCCAACTGTTCATTGCCCTGAGTGGCCGCGT

TTCGACGGCCACGACTATCTCGCCGAGGTTGCCGCCAAGGGCGCGGTGGCTGCGCTGGTG
GAGCGCGAAGTCGCCGACGCGCCCCTGCCGCAATTGCTGGTGCGCGATACCCGT

> ftsB (Q9HXZ6)
CATCTCCATCGAGGACGGCATGGACGAGTCCGACTGGGCCGGCTGGAAAGGCCTGACCGA
CAAGATCGGCGCCAAGGTCCAACTGGTCGGCGACGACCTGTTCGTGACCAACACCAAGAT
CCTCAAGGAAGGCATCGAGAAGGGCATCGGCAATTCGATCCTGATCAAGTTCAACCAGAT
CGGTTCGCTCACCGAGACCCTGGAGGCCATCCAGATGGCCAAGGCCGCCGGCTATACCGC
GGTGATCTCGCACCGCTCCGGCGAAACCGAGGACTCGACCATCGCCGACCTGGCCGTGGG
TACCGCCGCCGGTCAGATCAAGACCGGTTCGCTGTGCCGCTCCGACCGCGTGTCCAAGTA
CAACCAGTTGCTGCGCATCGAAGAGCAACTGGGCGCCAAGGCGCCGTACCGTGGTCGCGC
GGAATTCCGCGGCTGAGCCTAGTCTGGTACGGTGAAGGGGTGGCAAGAGCCACCCCTTTT
CTTCTCAAGGAGTTCGAGGC**TTG**AGGTTACGTAGCCCCTACTGGCTGTTCGTGGTGCTGA
TCCTGGCGCTGGCGGGCCTGCAATATCGCCTGTGGGTCGGCGATGGCAGCCTGGCGCAGG
TGCGCGACCTGCAGAAGCAGATCGCCGACCAGCATGGCGAGAACGAGCGCCTGCTGGAGC
GCAACCGGATTCTCGAAGCCGAAGTCGCCGAGCTGAAGAAAGGCACCGAGACCGTCGAGG
AGCGTGCGCGGCACGAGCTCGGCATGGTCAAGGACGGCGAAACCCTCTACCAGCTCGCCA
AG**TGA**GCTGACGACCCTTCTCCCGATGACGACTTCCGACCTTCCCGCCTTCTGGACGGTG
ATCCCCGCCGCCGGCGTGGGTTCGCGCATGCGCGCCGACCGCCCGAAGCAATATCTCGAT
CTTGCCGGTCGCACCGTGATCGAGCGTACCCTCGATTGTTTCCTCGAACACCCGATGCTC
AGGGGCCTGGTGGTCTGCCTGGCCGAGGACGATCCCTACTGGCCCGGGCTGGACTGCGCC
GCCAGCCGGCATGTG

> oprF (P13794)
AGGTGATGCTGAAAGTTTTGTACGGGTTGAAGAACTTTGAGGGCAAGTCGAAGTTCAAAA
CGTGGCTATATAGCATCACGTACAACGAGTGCATCACGCAGTACCGCAAGGAGCGCCGCA
AGCGCCGATTGATGGATGCGCTCAGCCTCGACCCGCTGGAGGAGGCTTCCGAGGAGAAGT
CGCCGAAGGTTGAGGAGCGGGGCGGATTGGATCGTTGGCTCGTCCATGTCAATCCGATAG
ATCGGGAGATTCTGGTTCTTCGCTTCGTCGCGGAACTGGAGTTTCAGGAGATAGCGGATA
TCATGCACATGGGTCTGAGTGCGACGAAAATGCGCTACAAGCGGGCACTGGACCGCCTGC
GCGAAAAGTTTTCAGATGCGACCGAAACATAGTTGGGTAAATATTGTCTCTCTATGCGGG
AAGTTCTGATAAACTTGCCACCCAAGTTGTGCGGCTGATTGTTGGACAACTAACTGACCA
TCAAGATGGGGATTTAACGG**ATG**AAACTGAAGAACACCTTAGGCGTTGTCATCGGCTCGC
TGGTTGCCGCTTCGGCAATGAACGCCTTTGCCCAGGGCCAGAACTCGGTAGAGATCGAAG
CCTTCGGCAAGCGCTACTTCACCGACAGCGTTCGCAACATGAAGAACGCGGACCTGTACG
GCGGCTCGATCGGTTACTTCCTGACCGACGACGTCGAGCTGGCGCTGTCCTACGGTGAGT
ACCATGACGTTCGTGGCACCTACGAAACCGGCAACAAGAAGGTCCACGGCAACCTGACCT
CCCTGGACGCCATCTACCACTTCGGTACCCCGGGCGTAGGTCTGCGTCCGTACGTGTCGG
CTGGTCTGGCTCACCAGAACATCACCAACATCAACAGCGACAGCCAAGGCCGTCAGCAGA
TGACCATGGCCAACATCGGCGCTGGTCTGAAGTACTACTTCACCGAGAACTTCTTCGCCA
AGGCCAGCCTCGACGGCCAGTACGGTCTGGAGAAGCGTGACAACGGTCACCAGGGCGAGT
GGATGGCTGGCCTGGGCGTCGGCTTCAACTTCGGTGGTTCGAAAGCCGCTCCGGCTCCGG
AACCGGTTGCCGACGTTTGCTCCGACTCCGACAACGACGGCGTTTGCGACAACGTCGACA
AGTGCCCGGATACCCCGGCCAACGTCACCGTTGACGCCAACGGCTGCCCGGCTGTCGCCG
AAGTCGTACGCGTACAGCTGGACGTGAAGTTCGACTTCGACAAGTCCAAGGTCAAAGAGA
ACAGCTACGCTGACATCAAGAACCTGGCTGACTTCATGAAGCAGTACCCGTCCACTTCCA
CCACCGTTGAAGGTCACACCGACTCCGTCGGCACCGACGCTTACAACCAGAAGCTGTCCG
AGCGTCGTGCCAACGCCGTTCGTGACGTACTGGTCAACGAGTACGGTGTAGAAGGTGGTC
GCGTGAACGCTGTTGGTTACGGCGAGTCCCGCCCGGTTGCCGACAACGCCACCGCTGAAG
GCCGCGCTATCAACCGTCGCGTTGAAGCCGAAGTAGAAGCTGAAGCCAAG**TAA**TCGGCTG
AGCCTCTAAGGAAAAACCCGGCCCAGGCCGGGTTTTTCTTTGCCTGGAAAAAGACCGCTC
GTCAGGCGCTCAGGGAAATCGGTTGCGACACGATGTCGCGGGTTACTTCGCCGATCACCA
GGATCGCCGGGCTTTTCAAGGCGAAACGTCCGGCGTCCCGCAGTAGCTCGCCGAGGTTGC
TGCGGCATTCGCGCTGGTTGCCCAGGGTGGCGTTCTCGATCATCGCCAGCGGCGTATCCT
CGG

> MexT (A0A069QAM9)
GGGTATGGGCGGGGAAACTGGCCACGCGGGAACCCAGCGGCAGGTCGCCGACGCCGGCGC
CGACCGCCAGCACCTCGCCGGCCAGTTCGAAGCCGAGACCGGACGGCAGCGCAGCCTGCT
CCGGGGCCAGGTTCTGACGCCAGAGCACATCCTTCCAGCTCACGCCGATCGCCTGGACAC

GCACCAGGACTTCCCCTGCGGCCGGCGCCGGGGTCGGCAGCTCTTCGCATTTGAGGACCT
CTGGCGGGCCAAACTGATGAAAACGGATCACTCGGGACATCGCAAACCTCTGCAGTGCAT
CACGGGGTGAATAACCTCATGGGTTGTGACTGTATCCGCCCATGCCTGACAAAACCACCC
GTCGTTATTGATAATGGCTATGCCTGTCAGTGATCCTATGCCCCTCCGGCACCTCGCCAG
GCCCCGCCCCGTCTCGCACGCAAGGCTTGACGGCGAGCCCCCGCGGTTGCAGCCTCTAGC
CCCTGGAAACGAGGAACGCC**ATG**AACCGAAACGACCTGCGCCGCGTCGATCTGAACCTGC
TGATCGTGTTCGAGACTCTGATGCACGAACGCAGCGTGACCCGCGCCGCAGAGAAACTGT
TCCTCGGCCAGCCGGCCATCAGCGCCGCGCTGTCGCGCCTGCGCACGCTGTTCGACGACC
CGCTGTTCGTCCGTACCGGACGCAGCATGGAGCCCACCGCGCGAGCCCAGGAAATCTTCG
CCCACCTGTCGCCGGCGCTGGACTCCATCTCCACCGCCATGAGCCGCGCCAGCGAGTTCG
ATCCGGCGACCAGCACCGCGGTGTTCCGCATCGGCCTTTCCGACGACGTCGAGTTCGGCC
TGTTGCCGCCCCTGCTCCGCCGCCTGCGCGCGGAGGCGCCGGGGATCGTCCTCGTCGTGC
GCCGCGCCAACTATCTATTGATGCCGAACCTGCTGGCCTCGGGGGAGATCTCGGTGGGCG
TCAGCTACACCGACGAACTGCCGGCCAACGCCAAGCGCAAGACCGTGCGCCGCAGCAAGC
CGAAGATCCTCCGCGCCGACTCCGCGCCCGGCCAGCTGACCCTCGACGACTACTGCGCGC
GACCGCACGCGCTGGTGTCCTTCGCCGGCGACCTCAGCGGCTTCGTCGACGAGGAACTGG
AGAAATTCGGCCGCAAGCGCAAGGTGGTCCTGGCGGTGCCGCAGTTCAACGGCCTCGGCA
CCCTCCTCGCCGGCACCGACATCATCGCCACCGTGCCCGACTACGCCGCCCAGGCGCTGA
TCGCCGCCGGCGGCCTACGCGCCGAGGACCCGCCGTTCGAGACCCGCGCCTTCGAACTGT
CGATGGCCTGGCGCGGCGCCCAGGACAACGACCCGGCGGAACGCTGGCTGCGCTCGCGGA
TCAGCATGTTCATCGGCGATCCGGACAGTCTC**TGA**GCCCTCCGGCAACTACCCGCACGAG
GCGTCGCAACGGGGAAATCGATCGCGCACCGCGAGCCTGTGGCTTATTCCATCGAAAGCA
CTGTCCATAACCATGGACTGTTACAGAAAACGAAAAACCATGTATCACTGTTCGTGATAA
TCAAAATCTCATCGTTCGATTAGTTCCCTGCCGGAGCAGCCCGCAGACTTCGCCAATCCC
GAAAAACCGACTGGCGGAGTCAAGCATGGTACAGTCATCCCACTT

> mexE (Q9I0Y9)
TTCGGCCGCAAGCGCAAGGTGGTCCTGGCGGTGCCGCAGTTCAACGGCCTCGGCACCCTC
CTGGCCGGCACCGACATCATCGCCACCGTGCCCGACTACGCCGCCCAGGCGCTGATCGCC
GCCGGCGGCCTACGCGCCGAGGACCCACCGTTCGAGACCCGGGCCTTCGAACTGTCGATG
GCTTGGCGCGGCGCCCAGGACAACGATCCGGCCGAACGCTGGCTGCGCTCGCGGATCAGC
ATGTTCATCGGCGATCCGGACAGTCTCTGAGCCCTCCGGCAGCTACCCGCACGAGGCGTC
GCAACGGGAAAATCGATCGCGCGCCGCGGGTGTGCGGCTTATTCCATCGAAAGCACTGTC
CATAACCATCGACTGTTACAGAAAACGAAAAACCATGTATCACTGTTCGTGATAATCAAA
ATCTCGTCGTTCGATTAGTTCCCTGCCGGAGCAGCCCGCAGACTTCGCCAATCCCGAAAA
ACCGACTGGCGGAGTCAAGC**ATG**GAACAGTCATCCCACTTCTCCTGGCGCTACCCCCTCG
CACTCGCGGCCGTACTGGTCCTGAGCGCCTGCGGCAAGGCCCCGGAAACCACCCAAGGCA
TGGCGGCGCCCAAGGTCAGCGTCGCCGAAGTCATCGAACAACCGCTGAACGAGTGGGACG
AATTCACCGGCCGCCTGGAGGCCCCGGAGTCGGTGGAGCTGCGCCCGCGGGTGTCGGGCT
ACATCGACCGCGTGGCCTTCCATGAAGGCGCACTGGTGAAGAAAGGCGACCTGCTGTTCC
AGATCGACCCGCGCCCGTTCGAGGCCGAGGTCAAGCGCCTCGAAGCCCAGCTGCAACAGG
CCCGCGCGGCCCAGGCGCGGAGCGTCAACGAAGCCCAGCGCGGCGAACGCCTGCGCGCCA
GCAACGCGATCTCCGCGGAACTCGCCGACGCCCGCACCACCGCCGCCCAGGAAGCCAAGG
CGGCGGTCGCCGCGACCCAGGCGCAACTGGACGCGGCGCGCCTGAACCTGAGCTTCACCC
GGATCACCGCGCCGATCGACGGTCGCGTCAGCCGCGCCGAGGTCACCGCCGGCAACCTGG
TCAACTCCGGGGAGACCCTGCTCACCACCCTGGTCAGCACCGACAAGGTCTACGCCTACT
TCGACGCCGACGAGCGCGTGTTCCTCAAGTACGTCGAGCTGGCCCGCCAGGCCGGTCGCG
ACACGCGCAGCGAGAGCCCGGTGTACCTCGGCCTGAGCAGCGAGGACGGCAACCCGCACC
TGGGCCGGCTGGACTTCCTCGACAACCAGGTCAACCCGCGTACCGGCACCATCCGCGGCC
GCGCCGTGTTCGACAACGCCAAGGGCGAGTTCACCCCGGGCCTCTACGTGCGCCTGAAGC
TGGTCGGCAGCAAGACCTACGCCGCCACCCTGATCAAGGACGAAGCGGTCGGCACCGACC
TGGGCAAGAAGTTCGTGCTGGTCCTGGATGGCGACAACAAGACCGTCTACCGCACCGTCG
AGATGGGACCGAAGCTGGAGGGCCTGCGCATCGTCCGCAGCGGCCTGAGCAAGGGCGACC
GGATCGTCGTGAATGGCCTGCAGCGGGTCCGCCCGGGCATGCAGGTGGATCCGCAGAAGG
TCGAGATGGCCAGCGCCGACACCCTGGCCACCCTCGCGCGCCTGCGGCAGTCGGTCGGCG
ACAGCGAACCACCGAAGGTGGCGGCGTCCAAGGACAACGCCACTCGCAACGAGCCGCGCG
GC**TGA**GCGCGGCGATAGGAAGAACCGATGAATTTCTCCCAATTCTTCATCCAGCGGCCGA
TCTTCGCCGCGGTGCTGTCGCTGCTGATCCTGATTGGCGGCGCCATCTCCCTGTTCCAGC
TACCCATCAGCGAATACCCGGAAGTGGTGCCGCCGACCGTCGTGGTCCGCGCCAACTTCC
CCGGCGCCAACCCGAAAGTCATCGGCGAGACCGTCGCCTCTCCCCTTGAACAGGCGATCA
CCGGGGTGGAGAACA

> mexF (Q9I0Y8)
GGCTGGACTTCCTCGACAACCAGGTCAACCCGCGTACCGGCACCATCCGCGGCCGCGCCG
TGTTCGACAACGCCAAGGGCGAGTTCACCCCGGGCCTCTACGTGCGCCTGAAGCTGGTCG
GCAGCAAGACCTACGCCGCCACCCTGATCAAGGACGAAGCGGTCGGCACCGACCTGGGCA
AGAAGTTCGTGCTGGTCCTGGATGGCGACAACAAGACCGTCTACCGCACCGTCGAGATGG
GACCGAAGCTGGAGGGCCTGCGCATCGTCCGCAGCGGCCTGAGCAAGGGCGACCGGATCG
TCGTGAATGGCCTGCAGCGGGTCCGCCCGGGCATGCAGGTGGATCCGCAGAAGGTCGAGA
TGGCCAGCGCCGACACCCTGGCCACCCTCGCGCGCCTGCGGCAGTCGGTCGGCGACAGCG
AACCACCGAAGGTGGCGGCGTCCAAGGACAACGCCACTCGCAACGAGCCGCGCGGCTGAG
CGCGGCGATAGGAAGAACCG**ATG**AATTTCTCCCAATTCTTCATCCAGCGGCCGATCTTCG
CCGCGGTGCTGTCGCTGCTGATCCTGATTGGCGGCGCCATCTCCCTGTTCCAGCTACCCA
TCAGCGAATACCCGGAAGTGGTGCCGCCGACCGTCGTGGTCCGCGCCAACTTCCCCGGCG
CCAACCCGAAAGTCATCGGCGAGACCGTCGCCTCTCCCCTTGAACAGGCGATCACCGGGG
TGGAGAACATGCTCTACATGTCCTCCCAGTCGACCTCCGACGGCAAGCTGACCCTGACCA
TCACCTTCGCCCTCGGCACCGACCTGGACAACGCCCAGGTGCAGGTGCAGAACCGCGTCA
CCCGGACCGAGCCGAAGCTCCCGGAAGAAGTGACCCGGCTCGGCATCACCGTCGACAAGG
CCTCGCCCGACCTGACCATGGTCGTGCACCTGACCTCGCCGGATAACCGCTACGACATGC
TCTACCTGTCGAACTACGCGGTGCTCAACGTGAAGGACGAACTGGCCCGCCTCGACGGCG
TCGGCGACGTCCAGTTGTTCGGCCTCGGCGACTATTCGCTGCGCGTCTGGCTGGACCCGA
ACAAGGTCGCCTCGCGCAACCTCACCGCCACCGACGTGGTCAACGCCATCCGCGAGCAGA
ACCGCCAGGTCGCCGCCGGCACCCTGGGCGCGCCGCCGGCGCCGAGCGATACCAGCTTCC
AGTTGTCGATCAACACCCAGGGTCGCCTGGTCACCGAGGAAGAGTTCGAGAACATCATCA
TCCGCGCCGGCGCCAACGGCGAGATCACCCGTCTGCGCGACATCGCCCGGGTCGAGCTGG
GCTCCAACCAGTACGCCCTGCGTTCGCTGCTGAACAACAAGCCGGCGGTGGCGATCCCGA
TCTTCCAGCGTCCCGGCTCGAACGCCATCGAGATCTCCAACCTGGTGCGGGAGAAGATGG
CCGAGCTGAAGCACAGCTTCCCGCAAGGCATGGACTACTCCATCGTCTACGACCCGACCA
TCTTCGTCCGCGGCTCCATCGAGGCGGTGGTGCACACCCTGTTCGAAGCCCTGGTGCTGG
TGGTGCTGGTGGTGATCCTGTTCCTGCAGACCTGGCGCGCCTCGATCATCCCGCTGGCCG
CGGTGCCGGTGTCGCTGATCGGCACCTTCGCGGTGATGCACATGCTCGGCTTCTCGCTCA
ACGCGCTGTCGCTGTTCGGCCTGGTGCTGGCCATCGGCATCGTGGTGGACGACGCCATCG
TGGTGGTGGAGAACGTCGAGCGCAACATCGGCCTCGGCCTCAAGCCGGTGGAAGCCACCA
AGCGTGCCATGCGCGAGGTGACCGGGCCGATCATCGCCACGGCGCTGGTGCTCTGCGCGG
TGTTCATCCCGACCGCGTTCATCTCCGGCCTCACCGGGCAGTTCTACCGCCAGTTCGCCC
TGACCATCGCGATCTCCACGGTGATCTCGGCGTTCAACTCGCTGACCCTGTCGCCAGCGC
TGGCGGCGGTCCTGCTCAAGGGCCACCACGAGCCGAAGGACCGCTTCTCGGTGTTCCTCG
ACAAGCTCCTCGGCAGTTGGCTGTTCCGTCCGTTCAACCGTTTCTTCGACCGCGCCAGCC
ATGGCTACGTCGGCACGGTGAACCGGGTCCTGCGCGGCAGCTCGATCGCCCTGCTGGTCT
ACGGCGGACTGATGGTGCTGACCTACTTCGGCTTCTCCAGCACGCCGACCGGTTTCGTCC
CGCAGCAGGACAAGCAGTACCTGGTGGCCTTCGCCCAGTTGCCCGACGCGGCCAGCCTGG
ACCGTACCGAGGCGGTGATCAAGCAGATGTCCGAGATCGCCCTGGCGCAGCCCGGCGTGG
CGGACTCGGTGGCCTTCCCCGGCCTGTCGATCAACGGCTTCACCAACAGCCCGAACAGCG
GCATCGTGTTCACCCCGCTGAAGCCGTTCGACGAGCGCAAGGACCCGAGCCAGTCGGCCG
GGGCCATCGCCGCCGCGCTGAACGCCAAGTACGCCGACATTCAGGACGCCTACATCGCGA
TCTTCCCGCCGCCGCCGGTACAGGGGCTGGGGACCATCGGCGGCTTCCGCCTGCAGATCG
AGGACCGTGGCAACCAGGGCTACGAGGAGCTGTTCAAGCAGACCCAGAACATCATCACCA
AGGCCCGTGCGCTGCCTGAGCTGGAACCCAGCTCGGTGTTCTCCAGCTACCAGGTCAACG
TGCCGCAGATCGACGCCGACATCGACCGCGAGAAGGCCAAGACCCACGGCGTGGCGATCA
GCGACATCTTCGACACCCTGCAGGTCTACCTCGGCTCGCTGTACGCGAACGACTTCAACC
GCTTCGGCCGTACCTATCAGGTCAACGTCCAGGCCGAGCAGCAGTTCCGCCTCGAACCCG
AGCAGATCGGCCAGCTGAAGGTGCGCAACAACCTCGGCGAGATGGTCCCGCTGGCGTCCT
TCATCAAGGTCAGCGACACCTCCGGTCCGGACCGTGTGATGCACTACAACGGCTTCATCA
CCGCCGAACTCAACGGCGCCCCGGCCGCGGGCTACAGCTCCGGCCAGGCGCAGGCGGCGA
TCGAGAAGCTGCTGAAGGAGGAACTGCCCAACGGCATGACCTACGAGTGGACCGAGCTGA
CCTACCAGCAGATCCTCGCCGGCAATACCGCGCTGTTCGTCTTCCCGCTCTGCGTGCTGC
TGGCCTTCCTCGTGCTGGCCGCCCAGTACGAGAGCTGGAGCCTACCGCTGGCGGTGATCC
TGATCGTGCCGATGACCCTGCTGTCGGCGATCACCGGGGTGATCCTGGCCGGCAGCGACA
ACAACATCTTTACCCAGATCGGCCTGATCGTTCTGGTGGGGCTGGCGTGCAAGAACGCGA
TCCTGATCGTCGAGTTCGCCAAGGACAAGCAGGAGGAAGGCATGGACCGCGTCGCCGCGG
TGCTGGAAGCCTGCCGCCTGCGCCTGCGGCCGATCCTGATGACGTCCATCGCCTTCATCA
TGGGTGTGGTGCCGCTGGTGATCTCCACCGGCGCCGGCGCCGAGATGCGCCATGCGATGG

GCGTGGCGGTGTTCTCCGGGATGATCGGGGTGACCTTCTTCGGCCTGCTGCTGACGCCGG
TGTTCTACGTCCTCATCCGCCGCTTCGTGGAGAACCGCGAAGCGCGCCGCGCCGCCAACG
ACAAAGGCCTGCCAGAGGTGCATGCA**TGA**TTCACGCGCAGTCGATCCGGAGCGGGCTCGC
GTCCGCCCTGGGTCTGTTCAGTCTGCTGGCGCTCAGCGCCTGCACGGTGGGTCCGGACTA
CCGGACCCCCGACACCGCGGCGGCGAAGATCGACGCCACGGCGAGCAAGCCCTACGACCG
CAGCCGCTTCGAAAGCCTGTGGTGGAAACAGTTCGACGATCCGACCCTGAACCAGTTGGT
CGAACAGTCGCTGAGCGGCAACCGCGACCTGCGCGTGGC

> oprN (Q9I0Y7)
TTCCTCGTGCTGGCCGCCCAGTACGAGAGCTGGAGCCTACCGCTGGCGGTGATCCTGATC
GTGCCGATGACCCTGCTGTCGGCGATCACCGGGGTGATCCTGGCCGGCAGCGACAACAAC
ATCTTTACCCAGATCGGCCTGATCGTTCTGGTGGGGCTGGCGTGCAAGAACGCGATCCTG
ATCGTCGAGTTCGCCAAGGACAAGCAGGAGGAAGGCATGGACCGCGTCGCCGCGGTGCTG
GAAGCCTGCCGCCTGCGCCTGCGGCCGATCCTGATGACGTCCATCGCCTTCATCATGGGT
GTGGTGCCGCTGGTGATCTCCACCGGCGCCGGCGCCGAGATGCGCCATGCGATGGGCGTG
GCGGTGTTCTCCGGGATGATCGGGGTGACCTTCTTCGGCCTGCTGCTGACGCCGGTGTTC
TACGTCCTCATCCGCCGCTTCGTGGAGAACCGCGAAGCGCGCCGCGCCGCCAACGACAAA
GGCCTGCCAGAGGTGCATGC**ATG**ATTCACGCGCAGTCGATCCGGAGCGGGCTCGCGTCCG
CCCTGGGTCTGTTCAGTCTGCTGGCGCTCAGCGCCTGCACGGTGGGTCCGGACTACCGGA
CCCCCGACACCGCGGCGGCGAAGATCGACGCCACGGCGAGCAAGCCCTACGACCGCAGCC
GCTTCGAAAGCCTGTGGTGGAAACAGTTCGACGATCCGACCCTGAACCAGTTGGTCGAAC
AGTCGCTGAGCGGCAACCGCGACCTGCGCGTGGCCTTCGCCCGCCTGCGCGCCGCCCGCG
CCCTGCGCGACGACGTGGCCAACGATCGCTTCCCGGTGGTCACCAGCCGCGCCAGCGCCG
ACATCGGCAAGGGCCAGCAACCGGGAGTGACCGAGGACCGGGTCAACAGCGAGCGCTACG
ACCTTGGCCTGGATAGCGCCTGGGAGCTTGACCTGTTCGGGCGCATCCGCCGTCAGCTGG
AGTCCAGCGACGCCCTCAGCGAAGCGGCCGAGGCCGACCTGCAGCAACTGCAGGTCAGCC
TGATCGCCGAGCTGGTGGACGCCTACGGCCAACTGCGCGGCGCGCAACTGCGCGAGAAGA
TTGCCCTGAGCAACCTGGAGAACCAGAAGGAGTCGCGCCAGCTCACCGAGCAACTGCGCG
ACGCCGGGGTCGGTGCCGAACTCGACGTACTGCGCGCCGATGCGCGCCTGGCGGCCACCG
CCGCCAGCGTGCCGCAACTGCAGGCGGAAGCCGAGCGCGCCAGGCACCGTATCGCCACCC
TCCTCGGCCAACGGCCGGAAGAGTTGACAGTGGACCTTTCGCCGCGCGACCTGCCGGCGA
TCACCAAGGCCCTGCCGATCGGCGATCCCGGCGAACTGCTGCGCCGCCGGCCGGACATCC
GCGCCGCCGAACGGCGCCTGGCCGCCAGCACCGCCGACGTCGGCGTGGCCACCGCCGACC
TGTTCCCGCGGGTCAGCCTCAGCGGCTTCCTCGGCTTCACCGCCGGGCGGGGCTCGCAGA
TCGGCTCAAGCGCCGCCCGCGCCTGGAGCGTCGGCCCGAGCATCAGTTGGGCCGCCTTCG
ACCTCGGCAGCGTGCGTGCCCGCCTGCGCGGCGCCAAGGCCGACGCCGACGCCGCGCTGG
CCAGCTACGAACAGCAGGTGCTGCTGGCCCTGGAAGAATCGGCGAATGCCTTCAGCGACT
ATGGCAAGCGCCAGGAGCGCCTGGTCTCGCTGGTCCGCCAGTCGGAAGCCAGCCGCGCCG
CCGCGCAACAGGCGGCGATCCGCTACCGCGAAGGCACCACCGATTTCCTGGTGCTGCTGG
ACGCCGAACGCGAGCAACTCTCCGCCGAAGATGCCCAGGCCCAGGCCGAGGTCGAGCTGT
ACCGCGGCATCGTGGCGATCTACCGCTCCCTCGGCGGTGGCTGGCAACCCAGCGCC**TGA**A
CCGGCTATCCCCCGGCCGATGGAAATCGGCCACTCCAGCCCCGCGATTGCAGCTCCTGGT
CGCGGGGCTTTTTTATGCGCAGCGCGGGGGCTCGGTCGAGCCCCACGTCGTTTCTCGGCG
ATGGCGTAGAGGCTCGGCGGCCATTGCCGCGGGCTATTCCCGCCGCTCCGGCTGCGCGGA
GTGGATATACAGGGTGTAGGTATCCGCCGGCCGGGGCGCGCCCGCCAGGTCGAGGGCATG
ACCGATGGC

> czcC (Q9I0W0)
ATAAAGTATGGATTCATTTGCAAGTAAATGTTTAAGTGCATTGCTATAAAGTTACATTTC
GGGCGTTGCCTGGATAGAATCCTGGAAATGTAATTTCATATCGTTGCGCTGCTTCGATGA
TAATTATCGAATGACCGGGTAGTTCTCCCGGGAGACCGCCTGAGAACTTTGGAAATGTCC
GCGGCGCAAGCACCGCTGGGCGTGAGGGGCAATGCCTTTTGTAAGCGCATGTTGCTTTTG
TCCCTATGTTTCATCCAACTATTGCCAGTCTCTTCGAAGGTTGCCGAAGTGTAACCGCTA
ATTGTCCGTGGAGAATATTCATTACCGACTTGTAATTCTTCCGTCACCTTGCCGATAAGC
CCCGCTACCTAACATCTGCCGCATCCCGGAAGTGCAACTTCGAATATCGCCGGCGAGATA
TCGCCGATGAACGGTTGCGTGGTTCCGGGCCGGTAATGGATCGCCGTCGCCGACGACGAT
CAGCAGACGAGGAGCGGAAC**ATG**CCGATACTTCGGCCACTGGCCAGCGCGGGGAAACGCG
CCTGCTGGCTACTGATGGGACTCTGCCTGGGCCTGCCGGCCCTGGCGAACGAGGCGCCGG
TGAGTTTCAACGGCACTTCGATCAGCCTCGAGCAGGCGTTGGAGCGGGCCCTGCGGAGCA
ACCCCGAGCTGGCGGCGGTGGGGCGCGAGACCGAGATCGCCAGCGGCGCGCGGCAGCAGG
CCGGGCTGATCCCCAACCCGGATCTGTCCTGGAGCGTCGAGGACACCCGCCAAGGCAATC

GCCAGACCAGCGTCAGCATCGCCCAGCCGCTGGAGCTGGGCGGCAAGCGCGGTGCGCGGG
TGGAGGTGGCGAAACGCGGCAGCGAGATCGCCTGGACCCAACTGGAAGTCCGTCGCGCCG
AACTGCGCGCCCAGGTCCGCGGCGCCTACTACGCGGCGCTGACCGCGCAGGAGCGGGTGC
GCCTGGCGAAGACCTCCCTGGACCTGGCCAGGCGCGCGCTGCAGGCCGCCGACCGACGGG
TCAAGGCCGGCAGCATCTCCTCGGTGGAACGGGTTCGCGCCCAGGTCCTGGCGGACAACG
CCCAGCTCGACCTGAGCCAGGCGGAGCTGGAGCAGCAGCGCACCTATGTGCAGCTGTCGA
GCACCTGGGACGAGCCGCAGCCAGGCTTCGCCAGGGTCGGCGGCGCGCTCGACGCGGTGC
CGGCGAGCATCACCCGTGGCGCCTTGCTGCGCCATCTCGACGAGTCGCCGACCCTGCGCC
TGGCGGCCCAGGAGGTAGCGCGCGGCGAGGCCCAGGTCGACCTGGAGAAGCGCCAGCGCA
TCCCCAACCTGACGGTCAGCATCGGCAGCAAGTACGACCAGACCGCCCGCGACGGACGTG
GCGAACGGGTCAACCTGATCGGCCTGTCGATGCCGCTGCCGCTGTTCGACCGCAACCAGG
GCAACATCTACGCCGCCCAGAGCCGCGCCGACCAGGCCCGCGACCTGCAGCGCGCGACCC
TGCTGCGCCTGCGCAGCGAAGCGGTACAGGCCTACGACCAGTTGCGCACCTCGGAACAGG
AGCTGGCGCTGGTCCGTCGCGACCTGCTGCCCGGCGCACAGAGCGCGCTGGACTCGATGA
CCCGCGGCTTCGAGATGGGCAAGTTCAACTTCCTCGACGTGCTCGATGCCCAGCGCACCC
TGGTCGGCGTCCGTGCCCAGTACGTGCGCGCGCTGGACGCCGCGGCACAGGCGCGGGTGA
GCATGGAGCGCCTGCTCGGCGAAGACATCGGCCACCTCGGCCAG**TGA**GATTTCCGGGGCG
GGCCGCCGGCGCGCCCCCCGAACGGTTCGGGAGAACAGAATGGGCAAGAAGACGATTATC
GCGGTGACCGCACTGGCACTGCTCGGCCTGGGCGGCGGTGCCTACCTGGAGCTGGGCGGC
AAGGCGCCGGCCGGCGCCGGCGAAGCGGCCGCCGAGGGCGAGGGCAGCGGACCGCGCGGC
GGCATGTTGTTGCGCCAGGGCGACCTGACCCTGGAACTGCTGGTGCCGGAGGACGGC

> czcB (Q9I0W1)
CATCGGCAGCAAGTACGACCAGACCGCCCGCGACGGACGTGGCGAACGGGTCAACCTGAT
CGGCCTGTCGATGCCGCTGCCGCTGTTCGACCGCAACCAGGGCAACATCTACGCCGCCCA
GAGCCGCGCCGACCAGGCCCGCGACCTGCAGCGCGCGACCCTGCTGCGCCTGCGCAGCGA
AGCGGTACAGGCCTACGACCAGTTGCGCACCTCGGAACAGGAGCTGGCGCTGGTCCGTCG
CGACCTGCTGCCCGGCGCACAGAGCGCGCTGGACTCGATGACCCGCGGCTTCGAGATGGG
CAAGTTCAACTTCCTCGACGTGCTCGATGCCCAGCGCACCCTGGTCGGCGTCCGTGCCCA
GTACGTGCGCGCGCTGGACGCCGCGGCACAGGCGCGGGTGAGCATGGAGCGCCTGCTCGG
CGAAGACATCGGCCACCTCGGCCAGTGAGATTTCCGGGGCGGGCCGCCGGCGCGCCCCCC
GAACGGTTCGGGAGAACAGA**ATG**GGCAAGAAGACGATTATCGCGGTGACCGCACTGGCAC
TGCTCGGCCTGGGCGGCGGTGCCTACCTGGAGCTGGGCGGCAAGGCGCCGGCCGGCGCCG
GCGAAGCGGCCGCCGAGGGCGAGGGCAGCGGACCGCGCGGCGGCATGTTGTTGCGCCAGG
GCGACCTGACCCTGGAACTGCTGGTGCCGGAGGACGGCAGCCCGCTCAAGGCCTGGGTCG
AGCGCGGCGGCAAGGCGCTGGCGCCGCAGGAGGTGGTCCTCGGCGCGGACGTCGAGCGTG
CCACCGGCGAGGTCGAGGACCTGCTGTTCAAGGCCAGCGGCGATGGCCTGGTGGCCAATG
CCGGGATCGCCGAGCCGCATGCCTTCACCGCGCGCCTCAAGCTGATGGCCGGCAAGCAGG
GCTACGACTTCGCCTTCACCCGCGAGGAGGGCAAGCTGGAGCTGGATGCCGAGCAGATCG
AGGCCGCCGGCATCACCCTCGATACGGCGCGGACGATCAGCCTGGCCCAGGTGGTCTCGC
TGCCCGGCGAGATCCGCTTCAACGAGGACCGCACCGCGCATATCGTCCCGCGCCTGCCGG
GGATCGTCGACAGCGTGCCGGCCAATCTTGGCCAGGCGGTGAAGCAGGGCGAGCTGCTGG
CGGTGATCAGCAGCCCGCAGTTGTCCGACCAGCGCAGCGAATTCGCCGCCGCCCAGCGCC
GCCTGAGCCTGGCGCAGAGCACCTACAAGCGCGAGCAGCAGCTGTGGAAGGAGGGCATTT
CCGCCGAGCAGGAATTCCTGCTCGCCCGCCAGGGTTTGCAGGAAGCCGAGATCGCGCTGA
ACAACGCGCGGGCGAAGATCGCCGCGCTCGGCGGCAATCCCAGCCTGCAGGGCGGCAACC
GCTACGAGCTGCGCGCGCCGTTCGCCGGGGTGCTGGTGGAAAAGCACCTGACCCAGGGCG
AGCCGGTGGACGGCACCGCCAACGTCTTCACCCTGTCCGACCTGTCCTCGGTCTGGGCCA
CCTTCAACGTGCCCGCGCAACTGCTCGGCCAGGTCCGCGTCGGCAGCAAGGTCAAGGTGC
TCGCCCAGGCGCTGGATAGCGAGGTGGAGGGCACCGTGTCCTACATCGGCGACCTGCTCG
GCGAGCAGACCCGCGCGGCCACCGCCCGGGTCACCCTGAGCAACCCCGAGAGCACCTGGC
GCCCGGGCCTGTTCGTTTCCGTGCAGGTGGCCGAGGCGACCCGCAAGGAAGTCCTGACGG
TCGCCGACGGCGCGGTGCAGAACGTCGACGGCGAGGACGTGGTGTTCGTCCGCGTGGCCG
ATGGCTTCGTGGTCCAGCCGGTCAAGCTGGGCATCAGCGACGGCCAGCGCGTCGAGGTCC
TCGAGGGCCTGCGCGCCGGCAGCCAGGTCGCCGCCAGCGGCAGCTTCATCCTCAAATCCG
AACTGGGCAAGGGCTCGGCGGAACACGGTCAC**TGA**GTCCCGCTCCAGGAGTCTTCGCATG
TTCGAACGCATCATCCAATTCGCCATCGAGCAACGCTGGCTGGTCCTGCTGGCGGTGCTG
GGGATGGCCGGGGTCGGCATCGGCAGCTACCAGAAGCTGTCCATCGACGCGGTCCCGGAC
ATCACCAACGTCCAGGTGCAGATCAATACCGCCGCCCCCGGCTACTCGCCGCTGGAAGTG
GAGCAGCGGATCACCTACCCGGTGGAGACCGTCATGGCCGGCCTG

> czcA (Q9I0W2)

CGACCTGTCCTCGGTCTGGGCCACCTTCAACGTGCCCGCGCAACTGCTCGGCCAGGTCCG
CGTCGGCAGCAAGGTCAAGGTGCTCGCCCAGGCGCTGGATAGCGAGGTGGAGGGCACCGT
GTCCTACATCGGCGACCTGCTCGGCGAGCAGACCCGCGCGGCCACCGCCCGGGTCACCCT
GAGCAACCCCGAGAGCACCTGGCGCCCGGGCCTGTTCGTTTCCGTGCAGGTGGCCGAGGC
GACCCGCAAGGAAGTCCTGACGGTCGCCGACGGCGCGGTGCAGAACGTCGACGGCGAGGA
CGTGGTGTTCGTCCGCGTGGCCGATGGCTTCGTGGTCCAGCCGGTCAAGCTGGGCATCAG
CGACGGCCAGCGCGTCGAGGTCCTCGAGGGCCTGCGCGCCGGCAGCCAGGTCGCCGCCAG
CGGCAGCTTCATCCTCAAATCCGAACTGGGCAAGGGCTCGGCGGAACACGGTCACTGAGT
CCCGCTCCAGGAGTCTTCGC**ATG**TTCGAACGCATCATCCAATTCGCCATCGAGCAACGCT
GGCTGGTCCTGCTGGCGGTGCTGGGGATGGCCGGGGTCGGCATCGGCAGCTACCAGAAGC
TGTCCATCGACGCGGTCCCGGACATCACCAACGTCCAGGTGCAGATCAATACCGCCGCCC
CCGGCTACTCGCCGCTGGAAGTGGAGCAGCGGATCACCTACCCGGTGGAGACCGTCATGG
CCGGCCTGCCGGGTTTGCAGGAAACCCGTTCGCTGTCGCGGCCGGGGATTTCCCAGGTGA
CGGTGATCTTCGAGGAGGGCACCGACATCTACTTCGCCCGCCAGCAGGTCAACGAACGCC
TGAGCACGGCGCGCGAGCAACTGCCGGAAGACATCTCGCCGACCCTCGGGCCGATCTCCA
CCGGTCTCGGCGAGATCTACCTGTGGACGGTGGAGGCCGAGGAGGGCGCGACCAAGGAGG
ACGGCAGCGCCTACACGCCCACCGACCTGCGCACCATCCAGGACTGGATCATCCGTCCGC
AGTTGCGCAACGTGAAGGGCGTGGCCGAGATCAACACCATCGGCGGCTACGCCAAGCAAT
TCCTCATCGCCCCGGATCCGAAGAAGCTCGCGGCCTACAAGCTGACCCTCGGCGACCTGC
AGAACGCCGTGCTGCGCAACAACGAGAACGTCGGCGCCGGCTACATCGAGCGGCGCGGCG
AGCAGTTGCTGATCCGCGCGCCGGGCCAGGTCAAGGACATGGACGATATCCGCGGGATAA
TCGTTTCCAACGTCGACGGCGTGCCGATCCGTATCCGCGACGTCGCCGAGGTCGGCCTGG
GCAAGGAGCTGCGCACCGGCGCGGCCACCGAGAACGGCCGCGAAGTGGTGCTCGGCACGG
TGTTCATGCTGATCGGCGAGAACAGCCGGGAAGTGGCCCAGGCGGTCGGCCAGCGCCTGG
AGGAGATCAACCGGACCCTGCCCAAGGGGGTCAAGGCGATCACCGTCTACGACCGCACCA
CCCTGGTGGACAAGGCCGTGGCCACGGTGAAGAAGAACCTGGTGGAAGGCGCGGCGCTGG
TGATCGCGGTGCTCTTCCTGTTCCTCGGCAACATCCGCGCGGCGTTGATCACCGCCACCA
TCATCCCGCTATCGATGCTGTTCACCTTCACCGGGATGGTCGGCAACAGGGTCAGCGCCA
ACCTGATGAGCCTCGGCGCGCTGGACTTCGGCATCATCGTCGACGGCGCCGTGGTGATCG
TCGAGAACGCCATCCGCCGCCTGGCCCACGCCCAGGCCCACCACGGCCGCCAGCTGACCC
GCGCGGAACGTTTCCACGAGGTCTTCGCCGCGTCCAGGGAGGCGCGCCGGGCGCTGGTCT
TCGGCCAGATCATCATCATGGTGGTGTACCTGCCGATCTTCGCCCTCACCGGGGTCGAGG
GGAAGATGTTCCACCCGATGGCGTTCACCGTGGTCACCGCGCTGCTCGGCGCGATGATCC
TCTCGGTGACCTTCGTCCCGGCGGCGATCGCGCTGTTCATCACCGGCAAGGTCAAGGAGG
AGGAGAACTTCGTCATGCGCCGCGCGCGTCTGGCCTACGAACCGGCGCTGCGCTGGGTGC
TCGGGCACCGCGCGCTGGTGGTCGGCGGCGCCCTCGGCGCGATCCTGCTCACCGGGCTGG
TGGCCTCGCGGATGGGCAGCGAGTTCATTCCCAGCCTCAGCGAGGGCGACTTCGCCATGC
AGGGCCTGCGGGTGCCGGGCACCAGCCTGACCCAGTCGGTCGAGATGCAGCAGACCCTGG
AGAGGAAGCTGATGGGCAAGTTCCCGGAGATCGAGCGGGTCTTCGCCCGCACCGGGACCG
CCGAGATCGCCTCCGACCTGATGCCGCCGAACGCCTCCGACAGCTACGTGATGCTCAAGC
CGCAGAGCCAGTGGCCGGACCCGAAGAAGTCGCGGGAGGCGCTGCTGGAAGAATTGCAGG
CCGCGGCCCTTGAGGTGCCGGGCAGCGTCTACGAGTTCTCCCAGCCGATCCAGTTGCGCT
TCAACGAACTGATCTCCGGGGTGCGCAGCGACGTCGCGGTGAAGGTCTTCGGCGACGACA
TGCAGGTGCTCAACGACACCGCGGAGAAGATCTCCAAGGTGCTGCAAGGCATCGACGGCG
CCTCCGAGGTGAAGGTCGAGCAGACCACCGGCCTGCCGGTGCTGACGGTGGACATCGACC
GCGACAAGGCGGCACGCTTCGGCCTCAATGTCGGCGACATCCAGGACACCGTGGCCACCG
CCCTCGGCGGACGCAACGCCGGTACCCTGTTCGAGGGCGACCGCCGCTTCGACATCGTCA
TCCGCCTGCCGGAAACCCTGCGCGCCGACCTGCCGGCACTGTCCAACCTGCTGATCCCGC
TGCCGCCGAACAACCTGGCGCGGATCGACTTCATCCCGCTGTCGGACGTGGCGCGCCTCG
ACCTCTCGCCGGGACCGAACCAGATCAGCCGGGAGAACGGCAAGCGGCGCATCGTGGTCA
GCGCCAACGTGCGCGGCCGCGACATCGGCTCGTTCGTGCTGGAAGCGCAACAGAAGCTGC
AGGACGGGGTGAAGATCCCGGCCGGCTACTGGACCACCTGGGGCGGCCAGTTCGAACAGT
TGCAGTCCGCCGCCAAGCGCTTGCAGGTAGTGGTGCCGGTGGCGCTGCTGCTGGTGTTCA
CCTTGCTCTTCGCCATGTTCAACAACGTCAAGGACGGCCTGCTGGTGTTCACCGGCATCC
CCTTCGCCCTCACCGGCGGGGTGCTGGCCCTGTGGCTGCGCGGGATACCGCTGTCGATCT
CGGCGGCGGTGGGTTTCATCGCCCTGTCCGGGGTGGCGGTGCTCAACGGCCTGGTGATGA
TCTCCTTCATCCGCAACCTGTTGCAGGAAGGACGCAGCCTCGACCAGGCGGTGTGGGAGG
GCGCCATCACCCGCCTGCGTCCGGTGCTGATGACCGCCCTGGTGGCGTCCCTCGGTTTCG
TGCCGATGGCCCTGGCCACCGGCACCGGCGCCGAGGTGCAGCGGCCGCTGGCGACGGTGG
TGATCGGCGGCATCCTGTCCTCGACCATGCTGACCCTGCTGGTGCTGCCGGTGCTCTATC

GCTGGACCCACGGGCGCGACGCCGGACCGCGCGAGGAGACGCCGGCGGCCGGC**TGA**AGCG
GGCGCGCAAGCGGGCCGGCAGGGGAAACCCTGGCGGCCCTTTTTCGTTGGCCGTTGGACG
GATTCCGTTCGATTACCGCACGCGATTGCCGGCCCGGATTGTCGCCGGCGGAGGGCTTGC
GTAGTGTGGGTGGACGTTCCGCGCCCGGCGCTTGGTGCCGCGCGGCGATAACAACAACGA
TAGCAGCCTGAGTGTCCTGCCCATGAATGCCTCCTTGCTCAGCGAGCGCAGCCGGGTGTT
CGAACG

> czcR (A0A072ZL37)
CCAGGACTTCTCGATCATGGCGCTGATCGGCATCGTGCTGCTGATCGGTATCGTCAAGAA
GAACGGCATCCTCATGGTCGACTTCGCCATCGTCGCCCAGCGCGAGCAGGGCATGAGCGC
GGAGCAGGCGATCTACCAGGCCTGCCTGACCCGTTTCCGGCCGATCATGATGACCACCCT
GGCCGCGCTGCTGGGCGCGATACCCCTGATGATCGGCTTCGGCACCGGTTCCGAGCTGCG
CCAGCCTCTGGGCATCGCGGTGGTCGGCGGGCTGCTGGTGAGCCAGGTGCTGACCCTGTT
CAGCACGCCGGTGGTATACCTGGCCCTGGAGCGGCTGTTCCACCGGCGCGGGGCGACGAC
CTCGGACGGCGGAACCGCTGGGGCGACGGCGACATGAGGCGTAACGGACACGGCTTTCCT
GGACGGCGGCGCGTGGCTAGGATGCAGGCAGGCATGCGTCCCGCCCGGGATGCGAAGGCG
AACAGAGGATCGGGCGATAG**ATG**CGGGTACTGATTGTCGAGGACGAGGCGAAGACGGCGG
ACTACCTGAACCGTGGCCTCAGTGAACAGGGGTTCACCGTGGACCTGGCGGACAACGGCA
TCGACGGTCGCCACCTGGCGCTCCATGGCGAGTACGACGTGATCGTGCTCGACGTGATGC
TGCCGGGCGTCGACGGCTACGGCGTTCTGCGGGCGTTGCGCGAGCGGCGGCAGACCCCGG
TGATCATGCTCACCGCGCGCGAGCGCGTGGAAGACCGGGTGCGCGGGCTGCGCGAGGGCG
CTGACGACTACCTGATCAAGCCGTTCTCCTTCCTCGAACTGGTTGCCCGCCTGCAGGCCC
TGACCCGGCGCGGCGGCAACCACGAAAGCCATTCGCAGATGCGCATCGCCGACCTGTCCA
TCGACCTGCTCAGCCGCAAGGTCTTCCGCGGCAACACTCGCCTGGAGCTGACTGCCAAGG
AGTACGCGCTGCTCTGCGTGCTGGCCCAGCGCAGCGGCGAGATCCTATCGAAGACGGCGA
TCGCCGAACTGGTCTGGGACATCAACTTCGATACCGATACCAATGTCGTGGAGGTGGCGA
TCAAGCGCCTGCGCGCCAAGCTCGACGGTCCGTTCGAGAACAAGCTGCTGCATACCATCC
GGGGCATGGGCTACGTCCTGGAGAACCGTGCGCTGGCGGAGTCGGGC**TGA**GGTGGGCATG
TCCTTGTCGACCCGCCTGGCGGGAATGTTCGCCGGCGCCGCGGTGGTGATCTTCGCCCTC
ATCGGCACCATGCTCTATTGCATCCTCGACCGCCAGACCGAGCGCCTGCAGCAGGGCGAG
GTGGACGTGCGCTTCAACATGGTCTCGCGGATGCTCGACCATCCCGACCTGGCCGAGCGC
TGGCCGCACCTGCAGGGCAAGCTGGACAACCTGAGCCAGGAGTACGAGCTGATCCGTTTC

> czcS (M4NAR4)
GACGGCTGGGACCTGCTCCGCCGCCTGCGCGAACGCAGCAGCGCGCGGGTGATGATGCTC
ACCGGGCATGGCAGGCTGACCGACAAGGTGCGCGGCCTCGATCTCGGCGCCGACGACTTC
ATGGTCAAGCCGTTCCAGTTCCCCGAACTGCTGGCGCGGGTCCGCTCGCTGCTGCGCCGC
CACGACCAGGCACCGATGCAGGACGTCCTGCGGGTCGCCGACCTGGAGCTGGACGCCAGC
CGCCACCGGGCCTTCCGCGGCCGGGTGCGGATCAACCTGACGACCAAGGAGTTCGCCCTG
CTGCACCTGCTGATGCGGCGCAACGGCGACGTCATCACCCGGACGCAGATCATCTCGCTG
ATCTGGGACATGAACTTCGACAACGACTCCAACGTGGTGGAAGTCGCCATCTGCCGCCTG
CGGGCGAAGATCGACGACGGCTTCGACCTCAAGCTGATCCATACCATTCGCGGCGTCGGC
TACGTCCTGGAAGCGCGCCG**ATG**ACGCCACCAGGCCGACTGTCGCTGCGCCTGGCCCTGC
TGGTCGGCGCCCTCGGCGCGCTGCTGGCCCTGGCGCTCGGCGCGATGGCGCACTGGAACC
TCGGCCGCGAGCTGGAAGCCCGCGAGCGGGAAAACCTGCAATTGAAGCTGGAGCAGATCC
GCCACAGCCTGGAAGACGATCTCGACCTGCGCAGCGACCCGGCCGTACAGGCGCATGCCC
TGCAGGACCAACTGGTGGCCCACAGCGGCCTGCACCTGAGCATTCTCGACAGCCGCAGCG
GCCAGCCGCTGATGAGCTTCGGCGACCAGGCCGCGGCCAGCGTCGCGGCCAACCGCGCGC
TTCTCGCCCGCTTGCAGGCGGACGCCCGCCAGCCGGTGTTCCAGAGCTGGAGCACCGGCA
ACGACCAGCGCCTGCTGAGCATCGGCGCTTCCATGCGCATGAAGAACGGCACCCCGGTAC
AGGTGTTGCTGAGCAGCGAGCGCAACGCCGACGAGCGCCTGCTCGACGGTTTCCTCCGCG
CCACCCTGCTGGCCCTGCCCTTCCTCCTGCCATTGATCGCCCTGGCCGCCTGGTGGGTGG
TGCGTGCCGGCCTCGAACCGTTGAACCGCTTCCGCCGGGTGGCCGCCCAGGTCAGCCCGC
AGGATCTCTCCTACCGCATCCCCGAGCAGAACCTGCCGCGCGAGCTGGACAGCCTGGCCC
GCAGCTTCAACCACATGCTCGGCCGCCTCGAGGATGGCGTACGCCAGCTCTCGCAGTTCT
CCGACGACCTGGCCCACGAACTGCGCGCGCCGATCTGCAACCTGCTGGTGCGCAACCAGG
TGCTGCTCAGCCAGCATCGCGACAGCGCGGCATACCGCGAAGCCCTGGAGTCCAATGCCG
AGGAACTGGAGCGGCTGTCGCGGATCGTCACCGACATGCTGTTCCTGGTGCAGGTGGACA
ACCCGGCGATCCAGGCCCAGTTCGGCTGCGTCGCCCTGCACGAGCAGGCGGCCAAGGTCA
TCGACCTGTACGAGATGGTCGCCGAGGACAAGGGCGTCGAACTGCGCCTGTCCGGCAGCG
GCTTCGCCCACGGCGACAACCTGATGATCCAGCGCGCCATTTCCAACCTGGTGTCCAACG

CCGTGCGCCACACCCCGCAAGGCGGGCGCATCGACGTGCGGATCGGGGAGCGCGCGGGGC
ACACCGAGGTGAGGGTCAGCAACGACGGCCCGGGCATCCCGCCGGAGTACCTGCCGCACC
TGTTCGAGCGCTTCTACCGCCGCGCCGGACGCCAGACTGGAGCCCAGGCCGGCACCGGCC
TGGGACTGGCCATCGTGCAATCGATCATGGCCTACCACGGCGGCCGCGCCGAAGCGGAAA
GCGTACCGCAGCAGAAGACCCACCTGCGCCTGCTGTTCCCTTCGACGGGCGCTGCC**TGA**C
GCCGGTTTCAGCAACCGGCGCGGCGCCGCCACAGCCGGTACTCGCCGCAGAAACCGGCGA
GGAACAGCGCCAGGCCGAGCGCCAGCAGGGTCCAGGGCGTCCGCAGCGGGGCGCGCTGGA
GCGGCGGATCGCCGTGCAGCAGGACGAAGCATTGCAGCAGCGCCCAGAACATCGCGGCGA
CGCCGCCAAGCATGGCCAGGCGGCACAACCAGCCGAGCCAGTAGCCTCGGCGCGAGGGCA
TTTCCGGAA

> gacA (Q51373)
CATCAGGAAGCAATCCTGGATCGTCGGCCGCCTTGGTCAGTGGGAATCGCCCTGGAGCGT
CTGGCTGAGGAATACCCTCGTTCGCCTGGTGCCGAATGCCAGTCGCAGGCGCCTCCACCA
GCGTCTTTTCACCGGTGTCGGTGAGATGGCCGCACAGTAGCCGCCGGTGCCAGTGGCGTC
TTGGTCGCCTGTTTCGACTTCGGCGATGGTCGCTATGGAGAAGTCCCGGGCAACCAGGGT
GCTTGCGCTTTACCAGGCCCGGCGTCGCTGGGCTCTCCACAGGTTCGCCGGTTTTTGCTG
CGATCCGGTCTGCCGCTCGCCGGGGGCTGGCGGTATACTTAGCAGCGCCCGTTCGATGCC
GTCGAATGATGTTTCCATGACGCTTGATTTATGACGCCTTTGCGCCAAGCTAGGTCATGG
GATGGCTTTCTGTTAAGGTTGCCGAAATCTCCTGGGGGCCCGTGTGGGCGGTTGTCTGCT
AATGCGCGACGAGGTGCAGC**GTG**ATTAAGGTGCTGGTGGTCGACGACCACGATCTGGTAC
GCACCGGTATTACCCGCATGCTGGCCGACATCGAAGGCTTGCAAGTGGTCGGCCAGGCCG
ACTGCGGTGAAGACTGTCTGAAACTGGCCCGCGAACTGAAGCCGGATGTCGTCCTGATGG
ACGTGAAGATGCCCGGTATCGGCGGCCTGGAGGCGACCCGCAAGCTGCTGCGCAGCCAGC
CCGACATCAAGGTCGTGGTAGTCACCGTCTGCGAAGAGGATCCGTTCCCCACCCGCCTCA
TGCAGGCCGGCGCCGCCGGCTACATGACCAAGGGCGCGGGGCTGGAGGAAATGGTCCAGG
CGATTCGCCAGGTCTTCGCCGGCCAGCGCTATATCAGCCCGCAGATCGCCCAGCAACTGG
CGCTGAAGTCCTTCCAGCCGCAGCAGCACGATTCCCCCTTCGATTCGCTGTCCGAGCGCG
AGATCCAGATCGCCCTGATGATCGCCAACTGCCACAAGGTGCAGAGCATCTCCGACAAGC
TGTGCCTGTCGCCGAAGACCGTGAATACCTATCGCTACCGCATCTTCGAGAAGCTCTCGA
TCACCAGCGACGTGGAACTGGCGCTGCTCGCCGTCCGCCACGGCATGGTCGATGCCGCCA
GC**TAG**ATGAGCGCCGTTTTCGACGCGAGCGCCTTCCTCGCTACCTGTAGCAATCGTCCGG
GCGTCTACCGCATGTTCGATGCGGAGGCCAAGCTTCTCTACGTGGGCAAGGCGAAGAGCC
TGAAGAAGCGCCTGGCCAGCTATTTCCGCAAGTCCGGCCTGGCGCCGAAGACCGCTGCGC
TGGTGGCGCGCATCGCCCAGGTGGAAACCACCATCACCGCCAATGAAACCGAGGCGTTGC
TGCTGGAGCAGACGC

> ftsK (Q9I0M3)
TTGCAACTCGGCGGTGTGGATGTGGTCGTAGACGATCTCGGTGTCGAAACGCTCGGCGTG
CTGCTGCATGCGGGTCATCAGCGCCGGGCCGGTAAGGCCTTCGACGTCGCCCGGCCAGTT
GTCGACTTCGGTGGTGGTGGTCAGCTGGCCGCCTGGCTGGATGCCGGTGATCACCACTGG
CTTGAGATTGGCGCGTGCGGCGTACACGGCGGCGGTATAGCCGGCCGGACCGGAGCCCAG
GATGATGAGGCGCGAATGCTTGACTTCACTCATAAAAAGACTCCATAAGCCTTTGTCACA
TAAGAAAATGCGTGCTCCAATGGAGCCGCCTGAAGAGTTCTGCCGGCTATGCTACACCTA
TCCGCAAAAGGCCGGCAAAACCCCGGGGCCGGGATCAGCCACGCACGGATTGCCGCTGAT
TTCGGGTGTGATTTTTCAGAGTTTCCCTAAAGCCGTACAATAGGCGCGTTTTGACCAAGA
CAGCTTCTAAAGCACCAGCC**GTG**CGCAGGAAAAATAGCGATTTGAAGGATTCGACCACCG
CGAGCCATGCCGCCGCATGGCGTCAGCAACTGCATTCCCGCCTGAAGGAAGGGGTCCTGA
TCGCGCTGGGCGCGCTCTGCCTCTACTTGTGGATGGCTCTGCTCACCTATGACTCGGCCG
ATCCGAGCTGGAGTCACTCGAGCCAGGTTGACCAGGTGCAGAATGCCGCCGGTCGGCTCG
GTGCGGTATCCGCCGATATCCTGTTCATGACGCTCGGCTACTTCGCCTACCTCTTTCCGC
TGTTGCTGGGCATCAAGACCTGGCAGGTATTCCGCCGCCGCAACCTGCCGTGGGAATGGA
ACACCTGGCTGTTTTCCTGGCGCCTGGTCGGGCTGATCTTCCTGATCCTGGCCGGCTCGG
CACTGGCCTACATCCATTTCCACGCCAGCGGCCACATGCCGGCCAGCGCCTCGGCCGGCG
GCGCCATCGGCCAGAGCCTGGGCCGCGTCGCGGTGGATGCGCTGAACGTGCAGGGCAGCA
CGCTGGTGTTCTTCGCCCTGTTCCTCTTCGGCCTGACGGTGTTCGCCGACCTCTCCTGGT
TCAAGGTGATGGATGTCACCGGCAAGATCACCCTGGACTTCTTCGAACTGATCCAGAACG
CCTTCAATCGCTGGATGGGCGCGCGCGCCGAGCGCAAGCAACTGGTCGCGCAACTGCGCG
AGGTCGACGAGCGCGTCGCCGAGGTGGTGGCGCCGAGCGTGCCGGACCGCCGCGAACAGT
CCAAGGCCAAGGAGCGCCTGCTGGAACGCGAAGAGGCCCTGGCCAAGCACATGAGCGAGC
GGGAGAAGCGCCCGCCGCCGAAGATCGATCCGCCACCGTCGCCGAAGGCCCCGGAGCCGA

GCAAGCGCGTGCTGAAAGAGAAGCAGGCGCCGCTGTTCGTCGATACGGCGGTGGAGGGCA
CCCTGCCGCCGCTGTCGCTGCTCGACCCGGCGGAAGTGAAGCAGAAGAGCTACTCGCCCG
AGTCGCTGGAGGCGATGTCGCGCCTGCTGGAGATCAAGCTGAAGGAGTTCGGCGTCGAGG
TCAGCGTGGACTCGGTGCATCCGGGCCCCGTGATCACCCGTTTCGAGATCCAGCCCGCGG
CGGGGGTCAAGGTCAGCCGCATTTCCAACCTGGCCAAGGACCTGGCGCGCTCGCTGGCGG
TGATCAGCGTGCGGGTGGTGGAAGTGATTCCCGGCAAGACCACTGTCGGCATCGAGATTC
CCAACGAAGACCGGCAGATGGTGCGCTTCTCCGAAGTGCTGTCGTCGCCGGAGTACGACG
AGCACAAGTCCACCGTGCCGCTGGCCCTGGGCCACGACATCGGCGGTCGGCCGATCATCA
CCGACCTGGCGAAGATGCCGCACCTGCTGGTGGCCGGTACCACCGGCTCCGGTAAGTCGG
TGGGGGTCAACGCCATGCTCCTGTCGATCCTGTTCAAGTCCACGCCGAGTGAGGCGCGAC
TGATCATGATCGACCCGAAGATGCTCGAACTGTCGATCTACGAAGGGATCCCGCACCTGC
TCTGCCCGGTGGTCACCGACATGAAGGAAGCCGCCAACGCCCTGCGCTGGAGCGTGGCGG
AGATGGAGCGGCGCTACCGCCTGATGGCGGCCATGGGCGTGCGCAACCTGGCTGGCTTCA
ACCGCAAGGTGAAGGACGCCGAGGAGGCCGGCACGCCGCTGACCGACCCGCTGTTCCGTC
GCGAAAGCCCGGACGACGAGCCGCCGCAGCTGAGCACCCTGCCGACCATCGTGGTGGTGG
TCGACGAATTCGCCGACATGATGATGATCGTCGGCAAGAAGGTCGAGGAGCTTATCGCCC
GTATCGCGCAGAAGGCGCGGGCGGCCGGTATCCACCTGATCCTGGCGACCCAGCGGCCGT
CGGTGGACGTGATCACCGGCCTGATCAAGGCCAACATTCCGACCCGGATCGCCTTCCAGG
TCTCCAGCAAGATCGACTCGCGCACCATCCTCGACCAGGGGGGCGCCGAACAACTGCTCG
GCCACGGCGACATGCTCTACCTGCCGCCGGGCACAGGCCTGCCGATTCGCGTACACGGCG
CCTTCGTTTCCGACGACGAGGTGCACCGGGTGGTCGAGGCCTGGAAGCTGCGCGGCGCGC
CCGACTACATCGAGGACATCCTCGCTGGCGTCGACGAAGGCGGCGGCGGTGGCGGTTCCT
TCGATGGCGGCGACGGCTCCGGCGAAGGCAGCGAGGACGACCCGCTCTACGACGAGGCGG
TACGTTTCGTCACCGAGAGTCGCCGGGCATCGATCTCCGCCGTGCAACGCAAGCTGAAGA
TCGGCTACAACCGCGCCGCGCGGATGATCGAGGCGATGGAGATGGCCGGCGTGGTCACCC
CGATGAATACCAACGGCTCCCGCGAAGTCATCGCGCCGGCGCCGGTTCGTGAT**TGA**ACCC
GACAGGTTTTAGAGGATTCCGATGCGACTGATCCGCACGTTGTTCGTTGCCGCCCTGGCC
ATGGGCGCTTCGCTGGCCCATGCCGACGACAGCGCCGCCGTCCAGCGCCTGACCGGCCTG
CTGAACAAGGCCCAGACCCTGACCGCGCGTTTTTCCCAGCTGACCCTGGACGGTAGCGGC
ACGCGCCTGCAGGAAACCGCCGGCCAACTGAGCCTGAAGCGGCCGGGACTGTTCCGCTGG
CATACC

> sagS (S6AG12)
GCCTCCACCACGCGGGGCAGGTCGGCCAGGTGGTCGCGTTCGATCTCGATCAGGCGCAGG
CCCGCGCCGGCGTGTTCGGCCAGCAGCGCGCGCACCAGGGAAGACTTGCCGGTGCCACGG
GCGCCCCAGAGCAGCGCGTGGTTGGCCGGCAGGCCATCGACGAACTGACGGGTATTGGCG
GCAAGCTGCTCGCGCTGCCTGTCGACGCCGATCAGGTCGGCCAGGGACAGGTCCAGGCTC
ACCTCCAGCGGTTGCAGGTAGCCGCTGCGGCCGTCGCGATGCCAACGCGCGGCCAGGCTC
TTCTGCCAGTCGATCTCCGGACGGATCGCCGGCAACAGGGGTTCGAGGCGCGCCAGCACC
GCATCGGCGCGCTCGAGAATTTCATACAGACGGGAATCCACGTTCAACTCCTTGACTCAG
CCGCAGTCACCAGGCGCTGCCGGCAGACCGGATAGGCTATGCTTGGCAAGCCCTCGGAAC
AAGGCCAAATCCTACGCCCC**ATG**GATATTCCGCTGACAAAACGGCTGTCGTTCAAGCAGG
CCAGCCTCACGGTGCTGGTGGCGTTCATCCTCGGCACCATCCTCAGCGTCATCCAGGTCG
CCAGCGACTACTTCAACGAAAACGCCTCGATCGACCGGGAGATCAAGGCGCTGCTGGAAA
TCTCCCACAGCCCCGCGGCACGCATCGCCTACAACATCGACGCCGAGCTGGCCCAGGAGC
TGGTGCTCGGCCTGTTGCGCTCGCCGGCGGTGATCCGCGCCGAGATCATCGACAACAATG
GCGCGGTGCTGTCCAGTGTCAGCCGCCCGCGCACCGAGAGCGACTTCCGCATCTTCAGCG
ACTACCTGTTCGGCGAACGCCGCGAGTTCGAGGACCCGCTCTACATCGTCCACGCGCCCA
ACGAAACCCTCGGCGTACTGCGCCTGGAGGTGGACACCTTCGCCTTCGGCAACCACTTCC
TGCGCCGCGCCATGTTCACCCTGCTCAGCGGCTTCGTGCGCAGCCTGCTGCTGTCGCTGA
TCCTCCTGGTGCTCTTCTACAGCATGCTGACCAAGCCGCTGGTGGAGCTGATCCGCGCCC
TCAGCGAACGCGACCCGCGCGCCGCGCCGGAAGCCAAGCTGCCCTGCCCGCCCGGCCACG
AACAGGACGAGATCGGCGTACTGGTGGAGGTCATCAATCGGCAACTGGCCAGCCTGTCCG
CGGAAATGCAGCAGCGCCGCGAAGCCGACCAGCGCCTCAACCAATACCTGGCGGAGCTGG
AAAACATCGTGTCGGCGCGCACCGCCGAGCTGAAGGCCGCCAACAGCCGCCTCAGCCGCT
ACAACCAGGAACTGGAAGCCGCGCGCGGCACCGCCCTGGAAATGGCCAAGGCTCGCGCCG
CCTTCCTCGCCAACATGAGCCACGAGATCCGCACCCCGCTCAATGGCCTGCTGGGCATGC
TCGACCTCGCCATCGACGGCCCGCTGAACAACGAGCAGCGCCGCCAGCTGCAGATCGCCC
ACAACTCCGGCAGCGTGCTGGTGGAGTTGCTCAACGACATCCTCGACCTGTCCAAGTTCG
AGGCCGGCCAGCTGGAGCTGGAGCAGATCCCCTTCGACCTCGGCACCCTGCTGGAAGACA
CCGCCAACCTGCTCTCGCAGAACGCCGGGGCGGAGGTGGAGCTGACCTGCCTGATCGACC

CGCAGCTGCCGGCGCTGCTGGTGGGCGACCCGACGCGGGTGCGCCAGGTGGCCAGCAACC
TCTTGTCCAACGCCCTCAAGTTCACCCGCTCCGGGCGCGTCGACCTGCGGGCCGCACCGA
GCCCCGGCGGCGTGCTGATCAGCGTGCGCGATACCGGTATCGGCATCTCCGCGCAGGCAT
TGCCCAAGCTGTTCCAGCCCTTCTCCCAGGGCAACGCGGGCATCACCCGGCAGTTCGGCG
GCACCGGCCTCGGCCTGGCGCTGACCCGGCACCTCTGCGAAGCCATGCAGGGCCAGCTGG
ACGTCAAATCCCAGGAAGGCATGGGCAGCGTGTTCAGTGCCGAACTGCCGCTGGTGGAAC
ACACCTCGGCCGAGCGGCGAGCGCCGCTACGGGGCAAGGTGATCGCCCTGTGCGACGCCA
AGAGCGGCCTGGCCGAGATGCTCGGCACCTGGCTGCCGTTATGGGGGCTGGAGCTGCAGC
GCCATGACGGCGACTTCGACCTCAGCGGCGTGCAGGCCGACCTGCTGATCAGCGACGACC
CCGAGCGCCTGGAGCCTTTGCGGCGCACCGGCCAACTGCCGATCCTGCTGGTGACCGGTT
ACGACAGCTTCCTGCCGCCACCCCGTGCGGCCACCCTGACACCGCTGCACCAACTGGCTC
GCCCGGTATCCCGCGCGGCGCTCTACCAGGCCCTGCAACGGGTGCTGCTGCAACTCCCGG
AGGAGGCGCCGGCGCCCGAGACGGCAATGGCCGAACCCCAGCACCGCCATGCGCGGGTAC
TGCTGGTGGAGGACAACGCGGTGAACCAGCTGGTGGCCAAGGGCATGCTCGCCAAGCTCG
GCTGCGAGGTGCTGCTGGCGGGACATGGCGCCGAGGCCCTGAGCCGCCTGGAACAGAGCA
CCGTGGACCTGGTGCTGATGGACTGCAACATGCCGGTCATGGATGGCTACGAGGCGACCC
GGCGCATCCGCCAGAGCGGGCGCTGGCCGGACCTGCCGATCATCGCCCTGACCGCCAACG
CCCTGCCGGACGAACGCGAGCGCTGCCGCGCCGCCGGCATGGACGACTACCTGGCCAAGC
CCTTCCGTCGCGAAGAGCTGGTGGCGCTGCTCGACACCTGGTTGCCCGCTACTCAGGCAG
TCCCA**TGA**GCTGGCCCAACAGCTCGCCCAGGCGCGCGCAGTTCGGCCATCTCGTTGAG
ATCCAGGCCGCTGGCGCAGATCAGCTCATTGCGCAGGGGTCCCACCTTGCCGCGCAGGGC
GGCGCCCTCCTCGCTCAGCGCCAGATGCACTTCGCGCTCGTCGTGGGCGGCGCGCCGGCG
CCGCACCAGCCCGAGTTGCTCCAGGCGCTTGAGCAGCGGCGTGAGGGTTCCCGAGTCCAG
CAACAGGCGCTCGCCCAG

> ladS (Q9HX42)
ATCTCGGCCACCGTGTCGACCATCTCCAGCGAACCGAGCATACCCAGCTTGACCGCCGCG
ACGGGCAGGTCGGCGAGTACGGCGTTGGCCTGGGCCAGCACCCACTCGCGGTCGAGGACG
CGGAAGTCGCTGACGTTGACGGTGTCCTGCACGGTCAGCGCGGTGACCGCCGGGGCGGCG
TGGCAGCCCTGGGCGAGCAGGGCTTCGATGTCGGCTTGCAGTCCGGCGCCACCACTGGGG
TCGTGACCGGACAGGCAGAGCACTACGGGGCGGGAAGTCGGTGTTTTCATGGCGCGTGAG
CTTACCACTAAAAGCGCTTCGTGGCGGCTGCGCGGACGACCTTTCGGGCACCGCGCCCGG
GCCCTGCGTGACACGTCCCGGGCCTGACGTGGCGTGGCGTGTGCTAAAGTTCGGCGAATA
TTCATACAACGCTCGCCAAGGGCGGACCATGACAAGGGAGGTGGGGCTCCCGGTCCGTTC
GAGGAGCACTGCGGGGCATC**ATG**CGGCACTGGCTGATTCTCTTTCTTCTTGCGTTGCCGT
GCCTGGCGGGTGCGGTGAGCTTCAACGAGCAGGTCGAGCGCCTGCCGCTGGGGCAGTCCA
TCGACGTGTTCGAGGACGTGCGCGGTAGCGCCGATATCAACGACATCACCTCGCGGGCCA
TCGACAGCAGCTTCCGCCGCCATGACAAGGACGTGCTCAATGCCGGCTATTCGCGCTCGG
TGTTCTGGTTGCGCCTGGACCTGGACTACCGGCCGGTCGCCTCCAGCGATCCGCGCACCT
GGCTGCTGGAGCTGGCCTATCCGCCGCTGGACAAGCTCGACCTCTACCTGCCCGATGGCC
AGGGCGGCTACCGCCTCGCCCAGCGCACCGGCGACACATTGCCGTTCGCCAGTCGGCCGA
TCCGGCAGAACAACTACCTGTTCGAACTCGGCCTCGAGCCGAACAAGCCGCAGCGTGTCT
ACCTGCGGCTGGAAAGCCAGGGGTCGATCCAGGCGCCGCTGACCCTGTGGTCGCCCAAGG
CCTACCTGGAGGAGCAGCCCGAACGCATCTATGTGCTCGGCATCATCTATGGCGTGCTGC
TGGTGATGCTGATCTACAACCTGTTCATCTTCCTCAGCGTCCGCGATACCAGCTATCTCT
ACTACATCCTCTATATCGCTTCGTTCGGGCTCTACCAGGTCTCGGTGAACGGCGCCGGCA
TCGAGTACTTCTGGCCCGACAGCCCCTGGTGGGCGAACGCCGCCACGCCATTCCTGATCG
GTTCGGCGGCGCTGTTCGGCTGCCAGTTCGCGCGCAGCTTCCTGCATACCCGCGACCACA
GCGTGTGGGTCGACCGCGGCCTGCTGGCGCTGATGGCGGTCGGCGCGCTGGTCATGCTGA
TGGCGCTGACCATGAGCTACGCCGTGGCCCTGCGCCTGGCCACCTACCTGGCGCTGGCCT
TCACCGGCCTGATCTTCGCCGCCGGCATCCTCGCCTGGCTGCGCGGCATGCGCGTGGCGC
GCTATTTCATCATCGCCTGGACCGCCTTCCTTCTCGGCGGCATCGTCAACACCCTGATGG
TCCTCGGCTACCTGCCGAACATGTTCCTCACCATGTATGCCAGCCAGATCGGTTCGGCGC
TGGAGGTGGGCTTGCTGTCGCTGGCACTGGCCGACCGGATCAACGCAATGAAGGAGGAGC
GCGCGCGAATCCTCCAGGAGTCCAGCCGCAAGCTGGAAGCGCTGAACCAGGAACTGGCCA
ACAGCAACCGCCTGAAGGACGAGTTCCTCGCCACCGTCACCCACGAACTGCGCACCCCGA
TGAGCGGGGTGATCGGTTCGCTGGAGCTGATGCAGACGGTGCCGATGGACGTCGAACTCG
CCGAGTACCAGCGCACCGCCGCCGGTTCGGCGCGGGACATGATGCGGATGGTCAACGACA
TCCTCGCCCTCATCGAACTGCAGGCCGGCAAGCTCTACCCGCGCCGCGAGCCGTTCAGCC
TGCGCGGCCTGTTCGACAGCCTGCGCGCGCAGTACGCGCCGCGCGTCGAGGAGAAGGGCC

TGCGCTTCGCCCTGCAACTGGACGACAGCCTGCCGGACACCCTCGAAGGTGATGCCGGCA
AGCTGGCCCAGGCGCTGGGCTACCTGGTCGACAACGCGATCAAGTTCACCGCCAGGGGCA
GCGTGACCCTGCGCGTCGCCGCCGGGCGTACCCATGACGGGGTGGCGCTGCGGGTGGAAG
TGATCGACACCGGCATCGGTTTCGACATGGCCGCCGGCAGCGACCTCTACCAGCGTTTCG
TCCAGGCCGACAGCTCGTTGACCCGTGGCTACGGAGGCCTCGGCATCGGCCTGGCGCTGT
GCCGCAAGCTGGTGGAACTGCTTGGCGGCGAGCTGACCCACGAGTCCCGGCCGGGGCAGG
GCAGCCGCTTCCTCCTGCGCCTGCAACTGACCCAGCCGGCCCAGGGCCTGGCGCCGCCGC
CGCGGCGCGCCGGCGGGCAGGCGGTGCGGCGTCCCGAGGAGTGCACGGTGCTGGTGGTGG
AGGACAACGCCATCAACCAGTTGGTGACTCGCGGCATGCTGCTCAAGCTCGGCTACCGGG
TGCGCACCGCCGACAACGGCAGCGAGGCCCTGGAGTTGCTGGCGCGCGAGCGGCCGGACG
GCGTACTGCTCGACTGCCAGATGCCGGTGATGGACGGCTTCGCCACCTGTCGGGCGATCC
GCGCGCTGCCCGGCTGCGCGGAACTGCCGGTGCTGGCGTTGACCGCCCACAGCCACAGCG
GCGATCGCGAGCGCTGCCTGGCGGCGGGCATGAGCGACTACATGGCCAAGCCGGTCAAGT
TCGAAGAGTTGCAGACCCTGCTGCACGATTGGCTGCTGTGCCAGCCGATCGTCACCAAGT
CCGCC**TGA**AGCCGTTCCGCGGCTGGCTGAAATGCCGGCCTGCGCGACCTGTATGTCTCTT
TAGGCAAAGCCTGAAGCGTGATTCACTGGGAAAAAGTGAATCCGGATTCAGTCATGGTCT
ATCGTGTCACCGAACGCCGCCTGCAACGCGATTCCGCGCTGCGCGAGCGCATCCTCCAGC
TAGGCCTGCGGCGTGTCGTGGAGGGCGGTTTCGCCGCGCTGACCATGCAGGCCCTGGCCG
ACGACGCGGGGATTGCCA

> ampR (P24734)
GCTGGCTGGCGCGGTCGTCGAGGCGCATCTTGTCCTGGGTCAGGGCATAGCCGGCGAGGG
TGGCGGTGAAGGTCTTGCTCACCGAGCCGATCTCGAACAGGGTCTCCGGCGTCACCCGGC
GGCCGTCCTCTTTCGAGGCCAGCCCATAGCTGAAGTAATGCGGTTCTCCTTTCAGGCTGA
TGGCTACGGCCAGGCCCGGAATGTCATTGGCCTTCATCACCGGTTGTACGGCGGCGTCGA
CCAGTGCCTTCAGGCGATCCGCCGGGGCCTCGCCGGCAATGGCCGGGGTGGTGGCGAACA
GCAGTGTGGAAGCGGCGATGCCGCACAGGCAGGGGAATCTGGTATCGCGCATGAGGATTG
GCGTCCTTTGTCGTTGGCTGCATGAGAAACCGCCCCGGGAGCGCTCCTTGATCGGCGGGC
GGGGAAAATCTAGGCTTGCGCAGGATTTGCGGCAAACGACGAATACTCGCGGCAGCCATT
AGAAAAATTTGGAGCAGAGA**TTG**GTTCGACCCCATTTGCCGCTGAACGCCCTGCGCGCCT
TCGAAGCTTCGGCCCGGCACCTGAGCTTCACCCGCGCGGCCATCGAGCTGTGCGTGACCC
AGGCGGCGGTCAGCCACCAGGTGAAGAGCCTCGAGGAGCGTCTCGGCGTGGCCCTGTTCA
AGCGTCTGCCGCGCGGCCTCATGCTGACCCACGAGGGCGAGAGCCTGCTGCCGGTGCTGT
GTGACTCCTTCGACCGCATCGCCGGCCTGCTGGAACGTTTCGAGGGTGGCCACTACCGGG
ACGTGCTCACCGTCGGCGCGGTCGGAACCTTCACGGTCGGTTGGCTGCTGCCGCGGCTGG
AGGACTTCCAGGCGCGCCATCCCTTCATCGATCTGCGCCTGTCCACCCACAACAACCGCG
TCGACATCGCCGCCGAGGGGCTCGACTACGCGATCCGCTTCGGCGGCGGCGCCTGGCACG
GCACCGAGGCGCTGGCGTTGTTCGAGGCGCCGCTGACGGTGCTCTGCTGCCCGGAGGTCG
CCGCCCAGTTGCACAGTCCCGCCGACCTGCTGCAGCACACCCTGCTGCGCTCCTACCGCG
CCGACGAGTGGCCGCTGTGGTTCCAGGCGGCCGGACTGCCGGCGCACGCGCCACTGACCC
GGAGCATCGTCTTCGACACCTCGCTGGCCATGCTCGAGGCGGCCCGCCAGGGTGTCGGCG
TGGCCCTGGCGCCGGCGGCGATGTTTGCCCGGCAACTGGCCAGCGAGAGCATCCGGCGTC
CGTTCGCCACCGAAGTGAGTACCGGCAGCTACTGGCTGACGCGCTTGCAGTCGCGGGGGG
AGACCAGCGCGATGCTGGCGTTCCGGGGGTGGTTGCTGGAGATGGCTGCCGTTGAGGCGC
GGGGGAGA**TAA**CGGTTATGCAGGCGATTCAAGTGTCGATGAAGAAGTAATCACCTTCCTC
GCGCCTTTTTTTATACGCTCGGGCCCTCCCAAGGTTGATACGGTTTGAGGCGTTGGGGTT
GTGATTGGAATTGCTGACGGCGTGGCGGATATGGACCGGCTCGTCGGCGTTGCTGATCGC
TCCGCTAAGCGTCACTCCCAGGCTGGCGGCATAGGCCCGGCGTGCCGCAACGATCTCGCT
CTCGAGCATGCCTGCTTTCCC

> ampC (P24735)
CCTGGAAGTCCTCCAGCCGCGGCAGCAGCCAACCGACCGTGAAGGTTCCGACCGCGCCGA
CGGTGAGCACGTCCCGGTAGTGGCCACCCTCGAAACGTTCCAGCAGGCCGGCGATGCGGT
CGAAGGAGTCACACAGCACCGGCAGCAGGCTCTCGCCCTCGTGGGTCAGCATGAGGCCGC
GCGGCAGACGCTTGAACAGGGCCACGCCGAGACGCTCCTCGAGGCTCTTCACCTGGTGGC
TGACCGCCGCCTGGGTCACGCACAGCTCGATGGCCGCGCGGGTGAAGCTCAGGTGCCGGG
CCGAAGCTTCGAAGGCGCGCAGGGCGTTCAGCGGCAAATGGGGTCGAACCAATCTCTGCT
CCAAATTTTTCTAATGGCTGCCGCGAGTATTCGTCGTTTGCCGCAAATCCTGCGCAAGCC
TAGATTTTCCCCGCCCGCCGATCAAGGAGCGCTCCCGGGGCGGTTTCTCATGCAGCCAAC
GACAAAGGACGCCAATCCTC**ATG**CGCGATACCAGATTCCCCTGCCTGTGCGGCATCGCCG
CTTCCACACTGCTGTTCGCCACCACCCCGGCCATTGCCGGCGAGGCCCCGGCGGATCGCC

TGAAGGCACTGGTCGACGCCGCCGTACAACCGGTGATGAAGGCCAATGACATTCCGGGCC
TGGCCGTAGCCATCAGCCTGAAAGGAGAACCGCATTACTTCAGCTATGGGCTGGCCTCGA
AAGAGGACGGCCGCCGGGTGACGCCGGAGACCCTGTTCGAGATCGGCTCGGTGAGCAAGA
CCTTCACCGCCACCCTCGCCGGCTATGCCCTGACCCAGGACAAGATGCGCCTCGACGACC
GCGCCAGCCAGCACTGGCCGGCACTGCAGGGCAGCCGCTTCGACGGCATCAGCCTGCTCG
ACCTCGCGACCTATACCGCCGGCGGCTTGCCGCTGCAGTTCCCCGACTCGGTGCAGAAGG
ACCAGGCACAGATCCGCGACTACTACCGCCAGTGGCAGCCGACCTACGCGCCGGGCAGCC
AGCGCCTCTATTCCAACCCGAGCATCGGCCTGTTCGGCTATCTCGCCGCGCGCAGCCTGG
GCCAGCCGTTCGAACGGCTCATGGAGCAGCAAGTGTTCCCGGCACTGGGCCTCGAACAGA
CCCACCTCGACGTGCCCGAGGCGGCGCTGGCGCAGTACGCCCAGGGCTATGGCAAGGACG
ACCGCCCGCTACGGGTCGGTCCCGGCCCGCTGGATGCCGAAGGCTACGGGGTGAAGACCA
GCGCGGCCGACCTGCTGCGCTTCGTCGATGCCAACCTGCATCCGGAGCGCCTGGACAGGC
CCTGGGCGCAGGCGCTCGATGCCACCCATCGCGGTTACTACAAGGTCGGCGACATGACCC
AGGGCCTGGGCTGGGAAGCCTACGACTGGCCGATCTCCCTGAAGCGCCTGCAGGCCGGCA
ACTCGACGCCGATGGCGCTGCAACCGCACAGGATCGCCAGGCTGCCCGCGCCACAGGCGC
TGGAGGGCCAGCGCCTGCTGAACAAGACCGGTTCCACCAACGGCTTCGGCGCCTACGTGG
CGTTCGTCCCGGGCCGCGACCTGGGCCTGGTGATCCTGGCCAACCGCAACTATCCCAATG
CCGAGCGGGTGAAGATCGCCTACGCCATCCTCAGCGGCCTGGAGCAGCAGGGCAAGGTGC
CGCTGAAGCGC**TGA**AGCGCGCTCGCGAGGGCGACGGAGCGTAGCGGCGCGGGACGCCGGT
CCTGGCTATGATGGTGCCATGAGCGCTTCCCCGCCCCTCCGCCAGCCCTGCCCGCCCGGC
GCCTGCGTCTGCGAACGCGAGCGCCTGGAGGCGCCCGGCGCGGACCGTCGCATCCTCCTC
CTGACCCGCCAGGAAGAGCAGCGCCTGGCCGCTCGCCTGGAAGCCCTGCGCAGCCTGGAA
GACCTGGAACACCTGCTGCGGCGC

> mexC (G3XD25)

GTGCGCCTTCGCCGTGCGGGTCGAGGAAGTCCGGGCGGTACTGGAATACCAGGAATACCA
GCAGCTCGCGGTGGGTGAGGTGTTCCTTGATCAGGCGCTGCAACGCCTCCAGAGGCTCGG
CATGCTCCAGGTCGCAGGCCTGGATGATCTGGTTCAGTACGGTCTCTCCGTGGTCCTCGA
GCATCTGCACCAGGTTGTCCCGCGTGCCGCAGAAGCGGTGCAGGGTGGCCTTGCTTACGC
CGGCCGCCTCGGCCAGTTCCTTCAGCGTCGCTCGCGGGCGGTCGACGATAGCGACTGCCA
GCGCCTTGATGAGTCGCTCGTCATGGGAAATCAGGGTCATCGATGGGTCCCGGTTGGTTT
TTCTGATTGTGCGCATTGTGCAGAAAACTGGCCTCGCGTCCAAAGGAATTAGCTGTCAAA
AGATCATTTGAGACAATATTGACTCAATTAGCTCGAAAAAGTACCATTCGATCCGTTTTC
AATCAACGGTCGGGTGTGTC**ATG**GCTGATTTGCGTGCAATAGGAAGGATCGGGGCGTTGG
CTATGGCCATCGCGTTGGCGGGTTGTGGGCCGGCGGAAGAGCGACAGGAGGCCGCCGAAA
TGGTGTTGCCGGTGGAGGTCCTGACGGTGCAGGCCGAGCCCCTGGCGCTGAGTTCGGAAC
TGCCTGGGCGGATCGAACCGGTGCGGGTCGCCGAGGTGCGCGCGCGGGTGGCCGGCATCG
TCGTGCGGAAGCGCTTCGAGGAGGGCGCCGACGTCAAGGCTGGCGACCTGCTGTTCCAGA
TCGATCCGGCACCGCTGAAGGCTGCGGTGTCGCGCGCCGAGGGTGAGCTGGCGCGGAACC
GCGCGGTGCTGTTCGAGGCGCAGGCGCGGGTGCGTCGCTACGAGCCGCTGGTGAAGATCC
AGGCGGTCAGCCAGCAGGACTTCGATACCGCCACCGCCGACCTGCGCAGCGCCGAGGCGG
CGACCCGCTCGGCCCAGGCCGACCTGGAGACCGCGCGCCTGAACCTCGGCTACGCCTCGG
TCACTGCGCCGATCTCCGGGCGCATCGGCCGCGCGCTGGTGACCGAGGGCGCGCTGGTCG
GGCAGGGCGAGGCGACGCTGATGGCGCGCATCCAGCAGCTCGATCCGATCTATGCGGATT
TCACCCAGACCGCGGCCGAGGCCCTGCGCCTGCGCGACGCCCTGAAGAAAGGCACCTTGG
CCGCCGGCGACAGCCAGGCGCTGACCCTGCGCGTCGAAGGGACGCCCTACGAGCGCCAGG
GCGCGTTGCAGTTCGCCGACGTGGCGGTGGATCGCGGCACCGGCCAGATCGCCCTGCGCG
GCAAGTTCGCCAACCCCGACGGGGTCCTGCTGCCGGGCATGTACGTGCGCGTACGTACGC
CCCAGGGCATCGACAACCAGGCGATCCTGGTGCCGCAACGGGCCGTGCACCGCTCCAGCG
ACGGCAGCGCCCAGGTGATGGTGGTGGGCGCCGACGAGCGCGCCGAGTCGCGCAGCGTCG
GTACCGGCGTCATGCAGGGTTCGCGCTGGCAGATCACCGAGGGCCTGGAGCCGGGTGACC
GGGTCATAGTCGGCGGCCTGGCTGCGGTGCAGCCGGGGGTGAAGATCGTGCCGAAGCCGG
ATGGTGCCCAGGCGCAAGCCCAGTCACCTGCGCCGCAACAG**TAA**GGGCGGGTTCGCCCCG
AGGATTTTCCGATGTCCGAATTCTTCATCAAGCGGCCGAACTTCGCCTGGGTGGTGGCCC
TGTTCATCTCCCTGGCCGGCCTGCTGGTCATTTCCAAATTGCCGGTAGCGCAGTACCCCA
ATGTCGCGCCGCCACAGATCACCATCACCGCCACCTATCCCGGCGCCTCGGCGAAGGTGC
TGGTGGACTCCGTCACCAGTGTGCTCGAGGAGTCGCTGAACGGCGCCAAGGGCC

> mexD (Q9HVI9)
GCACCTTGGCCGCCGGCGACAGCCAGGCGCTGACCCTGCGCGTCGAAGGGACGCCCTACG
AGCGCCAGGGCGCGTTGCAGTTCGCCGACGTGGCGGTGGATCGCGGCACCGGCCAGATCG
CCCTGCGCGGCAAGTTCGCCAACCCCGACGGGGTCCTGCTGCCGGGCATGTACGTGCGCG
TACGTACGCCCCAGGGCATCGACAACCAGGCGATCCTGGTGCCGCAACGGGCCGTGCACC
GCTCCAGCGACGGCAGCGCCCAGGTGATGGTGGTGGGCGCCGACGAGCGCGCCGAGTCGC
GCAGCGTCGGTACCGGCGTCATGCAGGGTTCGCGCTGGCAGATCACCGAGGGCCTGGAGC
CGGGTGACCGGGTCATAGTCGGCGGCCTGGCTGCGGTGCAGCCGGGGGTGAAGATCGTGC
CGAAGCCGGATGGTGCCCAGGCGCAAGCCCAGTCACCTGCGCCGCAACAGTAAGGGCGGG
TTCGCCCCGAGGATTTTCCG**ATG**TCCGAATTCTTCATCAAGCGGCCGAACTTCGCCTGGG
TGGTGGCCCTGTTCATCTCCCTGGCCGGCCTGCTGGTCATTTCCAAATTGCCGGTAGCGC
AGTACCCCAATGTCGCGCCGCCACAGATCACCATCACCGCCACCTATCCCGGCGCCTCGG
CGAAGGTGCTGGTGGACTCCGTCACCAGTGTGCTCGAGGAGTCGCTGAACGGCGCCAAGG
GCCTGCTCTACTTCGAGTCGACCAACAACTCCAACGGCACCGCCGAGATCGTCGTCACCT
TCGAGCCGGGCACCGATCCGGACCTGGCCCAGGTGGACGTGCAGAACCGCCTGAAGAAAG
CCGAGGCGCGCATGCCGCAGGCGGTGCTGACCCAGGGCCTGCAGGTCGAGCAGACCAGCG
CCGGTTTCCTGCTGATCTATGCGCTCAGCTACAAGGAAGGCGCTCAGCGCAGCGACACCA
CCGCCCTCGGCGACTACGCCGCGCGCAATATCAACAACGAGCTGCGGCGCCTGCCGGGCG
TCGGCAAGCTGCAATTCTTCTCTTCCGAGGCGGCCATGCGGGTCTGGATCGATCCGCAGA
AGCTGGTGGGCTTCGGCCTCTCCATCGACGACGTGAGCAATGCCATCCGCGGGCAGAACG
TGCAGGTGCCGGCCGGCGCCTTCGGCAGCGCACCGGGCAGTTCCGCGCAGGAGCTGACGG
CGACCCTGGCGGTGAAGGGCACCCTGGACGATCCGCAGGAGTTCGGCCAGGTAGTGCTGC
GCGCCAACGAGGACGGCTCGCTGGTCCGGCTCGCCGATGTCGCGCGCCTGGAACTCGGCA
AGGAGAGCTACAACATTTCCTCGCGACTGAACGGCACGCCCACCGTGGGCGGGGCTATCC
AGCTGTCGCCCGGGGCCAACGCGATCCAGACCGCTACCCTGGTGAAACAGCGTCTCGCCG
AACTGTCGGCGTTCTTCCCCGAGGACATGCAGTACAGCGTGCCCTACGACACCTCGCGCT
TCGTCGACGTGGCCATCGAGAAGGTGATCCACACCCTGATCGAAGCGATGGTCCTGGTGT
TCCTGGTGATGTTCCTGTTCCTGCAGAACGTCCGCTACACCCTGATCCCGTCCATCGTGG
TGCCGGTGTGCCTGCTGGGTACGCTGATGGTGATGTACCTGCTGGGGTTCTCGGTGAACA
TGATGACCATGTTCGGCATGGTCCTGGCGATCGGCATCCTGGTGGACGACGCCATCGTGG
TGGTGGAGAACGTCGAGCGGATCATGGCGGAGGAGGGGATTTCCCCGGCCGAGGCCACGG
TCAAGGCGATGAAGCAGGTATCCGGCGCCATCGTCGGCATCACCCTGGTGCTCTCGGCGG
TGTTCCTGCCGCTGGCTTTCATGGCCGGTTCGGTGGGGGTGATCTACCAGCAGTTCTCGG
TGTCGCTGGCGGTCTCGATCCTGTTCTCCGGCTTCCTCGCCCTGACCTTCACCCCGGCGC
TGTGCGCCACGCTGCTCAAGCCCATTCCCGAAGGGCACCACGAGAAGCGCGGCTTCTTCG
GCGCCTTCAACCGTGGCTTCGCCCGCGTCACCGAGCGCTATTCGCTGCTCAACTCGAAGC
TGGTGGCGCGCGCCGGACGCTTCATGCTGGTGTACGCCGGCCTGGTGGCCATGCTCGGCT
ACTTCTACCTGCGCCTGCCGGAAGCCTTCGTGCCGGCGGAAGACCTCGGCTACATGGTGG
TCGACGTGCAACTGCCGCCTGGCGCTTCGCGCGTGCGCACCGATGCCACCGGCGAGGAGC
TCGAGCGCTTCCTCAAGTCCCGCGAGGCGGTGGCTTCGGTGTTCCTGATCTCGGGCTTCA
GCTTCTCCGGCCAGGGCGACAATGCCGCGCTGGCCTTCCCAACCTTCAAGGACTGGTCCG
AGCGAGGCGCCGAGCAGTCGGCCGCCGCCGAGATCGCCGCGCTGAACGAGCATTTCGCGC
TGCCCGACGATGGCACGGTCATGGCCGTGTCGCCGCCACCGATCAACGGTCTGGGTAACT
CCGGCGGCTTCGCATTGCGCCTGATGGACCGTAGCGGGGTCGGCCGCGAAGCGCTGCTGC
AGGCTCGCGATACTCTTCTTGGCGAGATCCAGACCAACCCGAAATTCCTTTACGCGATGA
TGGAAGGACTGGCCGAAGCGCCGCAACTGCGCCTGTTGATCGACCGGGAGAAGGCCCGTG
CCCTGGGGGTGAGCTTCGAGACCATCAGCGGCACGCTGTCCGCTGCCTTCGGCTCGGAGG
TGATCAACGACTTCACCAATGCGGGGCGCCAACAGCGGGTGGTGATCCAGGCCGAACAGG
GCAACCGGATGACCCCGGAAAGCGTGCTCGAGCTATACGTGCCTAACGCTGCTGGCAACC
TGGTACCGCTCAGCGCCTTCGTCAGCGTGAAATGGGAAGAGGGACCGGTGCAATTGGTGC
GCTATAACGGCTACCCGTCGATCCGCATCGTCGGTGACGCCGCGCCCGGCTTCAGTACCG
GCGAAGCCATGGCGGAAATGGAGCGCCTGGCCTCGCAGCTGCCGGCCGGCATCGGCTACG
AGTGGACCGGCCTGTCCTATCAGGAGAAGGTCTCCGCCGGGCAGGCCACCAGCCTGTTCG
CCCTCGCCATCCTGGTGGTGTTCCTGTTGCTGGTGGCGCTCTACGAGAGCTGGTCGATCC
CGCTGTCGGTGATGCTGATCGTGCCGATCGGCGCCATCGGCGCGGTGCTCGCGGTGATGG
TCAGCGGTATGTCCAACGACGTGTATTTCAAGGTCGGCCTGATCACCATCATCGGTCTTT
CGGCGAAGAACGCGATCCTCATCGTCGAGTTCGCCAAGGAACTCTGGGAGCAGGGGCATA
GCCTGCGCGACGCCGCCATCGAGGCCGCGCGCCTGCGCTTCCGGCCGATCATCATGACTT
CCATGGCGTTCATCCTCGGCGTGATACCCCTGGCCCTGGCCAGCGGTGCCGGCGCGGCGA
GCCAGCGTGCCATCGGCACCGGAGTGATCGGCGGGATGCTCAGCGCCACCTTCCTCGGCG
TGCTGTTCGTACCTATCTGTTTCGTCTGGCTGCTGTCGCTGCTGCGCAGCAAGCCGGCAC

CCATCGAACAGGCCGCTTCGGCCGGGGAG**TGA**ACGAGATGCGCAAACCTGCTTTCGGCGT
ATCGGCGCTGCTGATCGCCCTGACCCTCGGCGCCTGCTCCATGGCGCCGACCTACGAACG
TCCCGCCGCGCCGGTGGCCGACAGCTGGAGCGGCGCCGCCGCCCAGCGCCAGGGCGCGGC
GATCGACACGCTGGATTGGAAGAGTTTCATCGTCGATGCCGAACTACGCCGCCTGGTGGA
CATGGCCCTGGATAACAACCGCTCGCTGCGCCAGACCCTCCT

> oprJ (Q51397)
GTGTTCCTGTTGCTGGTGGCGCTCTACGAGAGCTGGTCGATCCCGCTGTCGGTGATGCTG
ATCGTGCCGATCGGCGCCATCGGCGCGGTGCTCGCGGTGATGGTCAGCGGTATGTCCAAC
GACGTGTATTTCAAGGTCGGCCTGATCACCATCATCGGTCTTTCGGCGAAGAACGCGATC
CTCATCGTCGAGTTCGCCAAGGAACTCTGGGAGCAGGGGCATAGCCTGCGCGACGCCGCC
ATCGAGGCCGCGCGCCTGCGCTTCCGGCCGATCATCATGACTTCCATGGCGTTCATCCTC
GGCGTGATACCCCTGGCCCTGGCCAGCGGTGCCGGCGCGGCGAGCCAGCGTGCCATCGGC
ACCGGAGTGATCGGCGGGATGCTCAGCGCCACCTTCCTCGGCGTGCTGTTCGTACCTATC
TGTTTCGTCTGGCTGCTGTCGCTGCTGCGCAGCAAGCCGGCACCCATCGAACAGGCCGCT
TCGGCCGGGGAGTGAACGAG**ATG**CGCAAACCTGCTTTCGGCGTATCGGCGCTGCTGATCG
CCCTGACCCTCGGCGCCTGCTCCATGGCGCCGACCTACGAACGTCCCGCCGCGCCGGTGG
CCGACAGCTGGAGCGGCGCCGCCGCCCAGCGCCAGGGCGCGGCGATCGACACGCTGGATT
GGAAGAGTTTCATCGTCGATGCCGAACTACGCCGCCTGGTGGACATGGCCCTGGATAACA
ACCGCTCGCTGCGCCAGACCCTCCTGGATATCGAGGCGGCCCGCGCGCAGTACCGAATCC
AGCGCGCCGACCGGGTTCCGGGCCTGAATGCCGCTGCCACCGGCAACCGCCAGCGGCAGC
CGGCCGACCTGTCCGCCGGCAATCGCTCGGAAGTGGCCAGCAGCTACCAGGTCGGGCTGG
CCCTGCCGGAGTACGAACTGGACCTCTTCGGTCGGGTCAAGAGCCTGACCGACGCAGCCC
TGCAACAGTACCTGGCCAGCGAGGAGGCAGCGCGCGCGGCACGGATCGCCCTGGTCGCCG
AGGTCAGCCAGGCCTACCTGAGCTACGACGGCGCCCTGCGGCGCCTGGCGCTGACCCGTC
AGACCCTGGTCAGCCGCGAGTATTCCTTCGCCCTGATCGACCAGCGCCGCGCGGCCGGCG
CCGCCACCGCGCTGGACTACCAGGAAGCCCTTGGCCTGGTGGAGCAGGCGCGCGCCGAGC
AGGAGCGCAACCTGCGGCAGAAACAGCAGGCATTCAACGCGCTGGTGTTGCTGCTGGGTA
GCGACGATGCCGCGCAGGCGATTCCGCGGAGTCCGGGGCAGCGGCCGAAGCTGCTGCAGG
ACATCGCTCCCGGCACGCCGTCCGAGCTGATCGAGCGACGTCCGGACATCCTTGCCGCCG
AACATCGTTTGCGGGCGCGCAACGCGGATATCGGCGCGGCGCGCGCGGCGTTCTTCCCGC
GCATCAGCCTGACCGGCAGCTTCGGCACCTCCAGCGCGGAAATGTCCGGCCTGTTCGATG
GCGGCTCGCGCTCCTGGAGCTTCCTGCCGACGTTGACGCTGCCGATCTTCGATGGCGGGC
GCAACCGTGCCAACCTGAGCCTGGCCGAGGCGCGCAAGGATTCGGCGGTGGCCGCCTACG
AGGGGACCATCCAGACCGCTTTCCGCGAGGTCGCCGACGCCCTGGCGGCCAGCGATACCC
TGCGTCGCGAAGAGAAGGCCCTGCGCGCCCTGGCGAACAGCAGCAACGAAGCCCTGAAGC
TGGCCAAGGCACGCTACGAGAGTGGCGTCGACAACCACCTGCGCTACCTCGATGCGCAGC
GCAGCAGCTTCCTCAACGAGATCGCCTTCATCGACGGCAGCACCCAGCGGCAGATCGCCC
TGGTCGACCTGTTCCGCGCGCTCGGCGGAGGCTGGGACGAGGGACGGAGCCTGGTGGTAC
ATCGAGGCGGCAGGAGT**TGA**GCGGGTAAACGTGTGGGAAACGGTGCTAGCGGGTGGCAAC
TGCCGCCCGCTCAAGGGCTGGCAGGTTGTGCGGGGTTCGACGCGCCACGCAGGAACAGCT
CCTCCAGCGTGGCGGACAACCCGGCGCTGGCCGCGCGACCGCGGCGCTCGGCATCGACCA
TGCCGTAGATCAGGACGATGAAGAGTTCGCTGAAGGTCGCCGCGCTGATATCGATACGGA
ACACACGCTTCTGCTGGCCGCGCAGGAAGA

> nfxB (P32265)
GGTTCCGCGCCAGCTCACCCTCGGCGCGCGACACCGCAGCCTTCAGCGGTGCCGGATCGA
TCTGGAACAGCAGGTCGCCAGCCTTGACGTCGGCGCCCTCCTCGAAGCGCTTCCGCACGA
CGATGCCGGCCACCCGCGCGCGCACCTCGGCGACCCGCACCGGTTCGATCCGCCCAGGCA
GTTCCGAACTCAGCGCCAGGGGCTCGGCCTGCACCGTCAGGACCTCCACCGGCAACACCA
TTTCGGCGGCCTCCTGTCGCTCTTCCGCCGGCCCACAACCCGCCAACGCGATGGCCATAG
CCAACGCCCCGATCCTTCCTATTGCACGCAAATCAGCCATGACACACCCGACCGTTGATT
GAAAACGGATCGAATGGTACTTTTTCGAGCTAATTGAGTCAATATTGTCTCAAATGATCT
TTTGACAGCTAATTCCTTTGGACGCGAGGCCAGTTTTCTGCACAATGCGCACAATCAGAA
AAACCAACCGGGACCCATCG**ATG**ACCCTGATTTCCCATGACGAGCGACTCATCAAGGCGC
TGGCAGTCGCTATCGTCGACCGCCCGCGAGCGACGCTGAAGGAACTGGCCGAGGCGGCCG
GCGTAAGCAAGGCCACCCTGCACCGCTTCTGCGGCACGCGGGACAACCTGGTGCAGATGC
TCGAGGACCACGGAGAGACCGTACTGAACCAGATCATCCAGGCCTGCGACCTGGAGCATG
CCGAGCCTCTGGAGGCGTTGCAGCGCCTGATCAAGGAACACCTCACCCACCGCGAGCTGC
TGGTATTCCTGGTATTCCAGTACCGCCCGGACTTCCTCGACCCGCACGGCGAAGGCGCAC
GCTGGCAGTCCTACCTGGAAGCGCTGGACGCCTTCTTCCTGCGCGGACAGCAGAAAGGCG

TGTTTCGCATCGACATCACGGCGGCCGTGTTCACCGAACTGTTCATCACCCTGGTCTACG
GCATGGTCGATGCGGAACGTCGCGGACGGGCGGCCAGCTCCAATTCCGCGCATACCCTGG
AGCAGATGTTCCTCCATGGCGCCTCCAATCCGGCTCGCTCC**TGA**CCCTCGCGCCCGCGGC
GGCGCCCCTCTCGTCGCCGCATGGCACGGACCGATCCGCGTGCCGTCGACTTCGGCCGCC
CGGAGGCCTGCTCGACACTCGGGAAGATCAGTCCTCACCGGCCCTGCGACAAAGTGCCTC
GACAGCCGGAAAGGACCGTGACGCTCGTCGGCGATACTGGCACTTGGCCTTGCTTCCGGG
CATCCTAACTGTCTACCGAATCACACCTCCCCCAGGAATTCCGGTCAGTGTCGA

> morA (Q9HVI8)
CGGACTTCCTCGACCCGCACGGCGAAGGCGCACGCTGGCAGTCCTACCTGGAAGCGCTGG
ACGCCTTCTTCCTGCGCGGACAGCAGAAAGGCGTGTTTCGCATCGACATCACGGCGGCCG
TGTTCACCGAACTGTTCATCACCCTGGTCTACGGCATGGTCGATGCGGAACGTCGCGGAC
GGGCGGCCAGCTCCAATTCCGCGCATACCCTGGAGCAGATGTTCCTCCATGGCGCCTCCA
ATCCGGCTCGCTCCTGACCCTCGCGCCCGCGGCGGCGCCCCTCTCGTCGCCGCATGGCAC
GGACCGATCCGCGTGCCGTCGACTTCGGCCGCCCGGAGGCCTGCTCGACACTCGGGAAGA
TCAGTCCTCACCGGCCCTGCGACAAAGTGCCTCGACAGCCGGAAAGGACCGTGACGCTCG
TCGGCGATACTGGCACTTGGCCTTGCTTCCGGGCATCCTAACTGTCTACCGAATCACACC
TCCCCCAGGAATTCCGGTCA**GTG**TCGACCCCCTCGCTAACCCCGTCGCCGCCTCCCGCCG
GCGACCCACTGACCGTACCCGACCGACCGGCAGCCAAGGCTGGCCTGCTGTTGCTGGTCC
TGTTGATGCTCGGCCTGCTGCTCTGGCAGATGAGCCAGGAGCTGCGCCAGCAGGAACGCT
TCGAACACGAGCGGGCCGCGGCCCAGCTCGATCGTCTCAACGACCGCCTGTCCCTGACCC
TCGAACTCAAGGCGCGCACCGCCCTCGCCCTGCTCCCGGGCGTGCCCCCCAGCGAGCGTG
GCGAGATACAGGGCAGGCTGTTGCCGCGCATCAGCGATGCGCTGCCGCAGGTGCGCCAGT
TGCAATGGGTGGACAGCGCCCCGCAGACGGACAGCCCGTCGCCGGAAACGACGCTGCCCG
AGCAGTTGCGCCAGCACGCCGGCTCCGGTCTCTACCACTATTGCCTCGACCCGCGCGACG
GCGAATCCCTCTACCTGACCCTGCGCGAACCCGGCAGCCGCCGCGACAGTGGCTTCTGGC
TGTTGCACCTGGCCAGCGACAGCCTCGCCCAGTGGAGTCCCGACCTGCCCACCGGCAACC
TGCTCTGGCGCCTGGAAGACCAATACGCCGGGCGGGTGCTCTGGCATACCCCGGGGAGCA
CGGCGCTGCTCGACGACATGCAGACCCTCGGCGTGGAACCGCTGCGCAACAGCGACTGGC
AGCTTCGCGGACTCTACGACAGCACGCGAGTGCGGCTCGGCCTGCTGCCGGGAATCGGCG
GCGAGCTGGCGATCTTCCTCCTGCTGGTCGGCGTCACCGTGTACATGCTGCTGCGCCTGC
ACCGCGAGCAACAGGGCCTGCGCGCCATGACCCTGGCCTCGCAGCGCAGCCTGCGCCAGG
CGGCCACGGCACTGGCGGCGATCGACGAGCGGGTGCTGGTGACCCGCGCCGACGGCCGCC
TCAGCTACCTCAATCCCCAGGCCGAACGCCTCTTCGGCATCAGTTCGGCCCAGGCCCGCC
AGCATCACCTGCTGGGCCTGCTGCCGGACCTCGAGCCAGGCTGGCTGACGGACGCCGGTG
GCGACGGCGAGACCCGTAGCGAGTTGCTGCCGCTGCGGGTACGCGGCGAGCCGCGGACGT
TCACCCTCAGCCGCCATCCGCTGGCCAGCTATCCGGCGCTCGCCGACCAGGCCTGGACCC
TCCGCGAGCAGATCCTCCAGGGCGGGCAGGTCTGGGTGTTGCGCGACGTCACCGAGGAAC
AGCACGCCCTCGGCGTCCTGGAGGAAACCCGGCGGCGCTACCAGGACATCTTCGAAGGGG
TCGGCGTGGCCCTCTGCGTGCTCGACCTCTCCGCCCTGCGCCAGGCTCTTGTGGAGCAAG
GCTTGGACAGCTGCGCCGCACTGCGCGCCTGGCTCGCCGGCGAACCCGGGCGGCAGCAGG
CGCTGCTGGAGCGGATCCGCATCACCGAGATCAACGATGTCAGTCGCCGCCTGCTACAGA
TCGATTCCACCGAACAGGCCTGGCAGCGCTTGCTCGACCACGGCCCGCTACAACCGGACA
GCCTGCGCCTGCAGGTGATCGAGGCGCTCATCGAGCGCCGGCCGGTACTCGAACTTGAAG
CCACGCTGCGCTGCGGCAAGAGCACGCCGCGGCACCTGTGGTTGCAACTGCGCCTGCCGG
AAAACCCCGGCGAATGGCATGCGGTGACCCTCAGCCTGTCCGACGTCACCAGTCGCAAGG
AAGTCGAACTGTCGCTGATCGAGCGCGAGAAGTTCTGGTCGGACGCGGTCCAGGCGGTGC
CCGATACCCTCTACATCCACGACCTGCACGCGCGCCGGGTGATCTTCAGCAACCATCACC
TGGGCCAGCAGTTGGGCTACAACCAGGAAGAACTGGCGCGGATGGGCGAGAACTTCTGGG
AAACCCTGCTGCACCCCGACGACCAGGAATACTACTGGCGCATCCGCAACCTGCAGCACG
TGGTCGGCGACGGCCTGCTGCTCGACAGCCAGTTGCGCTGGCGCCACCGCGACGGCCGCT
GGCACTGGTTCGACATCCGCGAGCAGGCCTTCAGCCGCGACCGCAGCGGTCGCGTGGCGC
GCCTGATCGGCGTGGCCAAGGACATCACCTTCACCGTCGAGGCGAACAACGCGCTGCGCG
AGAACGGCCGACGCTACCGCATGCTGGCGGAAAACATCAGCGACGTGATCTTCTCCACCG
ACGCCGAACTCAACGCCAGCTACGTCAGCCCCTCGGTACAGCACGTGTTCGGCTACAGCC
CGGAATGGGCGCTGCTCAACGGCCTCCATCAGACCGCCACCAACCCGCGCCAGCTCGGCC
GCCTGAACGGCCTGCTGCGCCGCGTGCGGCACGCCATCGGCGACCGCCAGAAGCTCGCCG
AGCTGCGGGAAAACCCTGGCCAGCATCTGTTCGCCCTCGACTGCCTGCGCGCCGACGGAC
GCAAGATTCCCATCGAACTGCGGATCGTCCTGATGTGGGACGAGCACGACCGCTTCGAAG
GCCTGCTCGGCATCGCCCGCGACATCAGCCAGCAGCGCCGCGCCGAACGCGAGCTGCGGA
TGGCGGCGACGGTATTCGAACACTCCACGGCGGCGATCATGGTCACCGACCCGGCCGGCT

ACATCGTCCAGGTCAACGATTCCTTCTCGCGCCTGACCGGCTACAGCCCGGCCGACGTGC
TCGACCAGCAGCCGCGCCTGCTCACCGCCGACCGCCAGGAAGCCAACCAGCTCAAGCACG
TGCTCGCCAGCCTGCAGCACAGCGGCAGCTGGGAAGGCGAGATCCTGCAGAAGCGCAAGA
CCGGCGAGCTCTATCCATCCTGGGTCGGCATCACCGCGGTGCGCGACGAGGAAGGCGACC
TGGTCAGCTTCGTCTGCTTCTTCAGCGACATCAGCGAGCGCAAGGCCAGCGAGCGGCGCA
TCCACCGCCTGGCCTACTACGACGCCCTCACCCACCTGCCCAACCGCACGCTGTTCCAGG
ACCGTCTGCATACCGCCCTGCAGCAGGCCGAGCGCAACGGCCAGTGGGTGGTGCTGATGT
TCCTCGACCTCGACCGCTTCAAGCCGATCAACGACTCCCTCGGCCACGCCGCCGGCGACC
GCATGCTGCAGGAAGTGGCGACCCGCCTGAGCGCCTGCGTCAGCCAGGACGATACCGTGG
CGCGCATGGGCGGCGACGAATTCACCCTGCTGCTGCCTTCCCAGGGCGACCGCGAGATCG
CCCTGAAACGGGCGATCCAGGTCGCCGAGCTGATCCTCGGCCGTCTGGCCCGGCCGTTCA
CCCTGGAAGGCCGTGAATTCTTCGTCACCGCCAGCATCGGCGTGGCCTTGTCGCCCCAGG
ACGGCGCCGAACTGAGCCTGCTGATGAAGAACGCCGACACGGCGATGTACCACGCCAAGG
AGATGGGCAAGAACAACTTCCAGTTCTACCAGGCCGAGATGAACGCTCGCGCCCTGGAGC
GCCTGGAACTGGAAAGCGACCTGCGCCGCGCCCTTGAACTGGGCGAGTTCGTCCTCCACT
ACCAGCCGCAGTTCACCGGCGACGGCCGCCGCCTGACCGGCGCCGAGGCCCTGCTGCGCT
GGCAGCATCCGCGCCGCGGACTGGTTCCGCCGAGCGAGTTCATTCCGGTGCTGGAGGAGA
TCGGCCTGGTGGCCCAGGTCGGCGACTGGCTGCTGGCGGAAGCCTGCAAGCAGTTGCGCA
GCTGGCACAAGGCCAAGGTGCGGGTGCCAAAGGTCTCGGTGAACCTCTCGGCACGGCAGT
TCGCCGATGGCCAGTTGGGCGAGCGGATCGCCGCGATCCTCTACGAGACAGGCATCCCGC
CGGCATGCCTGGAGCTGGAACTGACCGAAAGCATCCTGATGAGTGACGTCGCCGAGGCCA
TGCAGATCCTCTCCGGGCTCAAGCGCCTCGGCCTGGCCATCGCGGTGGACGACTTCGGCA
CCGGCTACTCCTCGCTGAACTACCTCAAGCAGTTCCCCATCGACGTGCTGAAGATCGACC
GCAGCTTCGTCGACGGCCTGCCCCACGGCGAACAGGACGCACAGATCGCCCGGGCGATCA
TTGCCATGGCCCACAGCCTGAACCTGATGGTGATCGCCGAGGGCGTGGAGAGCCAGGCCC
AGCTGGACTTCCTTCGCGAGCACGGCTGCGACGAGGTGCAAGGCTACCTGTTCGGCCGGC
CGATGCCCGCCGAGCAGTTCGGCATGCTCTACGCCAGCGACGTGCTGTTCATGTTCAACG
AGGGC**TGA**GCCGCCGTGCGGCGCGCTTGAGCGCCGCTTGTCCGCGACATGATGGCCTTTC
ATGTGCCAGCACCCGCCGCATGGGTTAGAATGCGCGCCTTTTCCCAGCACCGATCCTTAT
GAGGACCGCCATGTTCAGCCGTGATTTGACCCTCGCCCGCTACGATGCCGAACTCTTTGC
CGCGATGGAGCAGGAAGCCCAGCGCCAGGAAGAGCACATCGAGCTGATCGCCTCCGAGAA
CTACACCAGCCCGGCGGT

> cbrA (G3XCM7)
GGAACTGCTGGGGTTGAGCCAGCCGCGCTACCTGCACGTGCCGCTGATCGTCCAGCCGGA
CGGGCACAAGCTCGGCAAGTCCTACCGCTCCCCACCACTGCCCGCCGAGCAGGCCGCCGC
GCCGCTGACCCGCGCCCTGCGCGCGCTCGGCCAGCGGCCGCCGGCCGAACTGGCCCAGGC
CAGCGCCAGCGAGGCGCTGGCCTGGGGCGTGGCGCACTGGGACGCCACGCGCATCCCGCG
CTGCGCTACCCTTCCCGAGGAACGCCTGTAGCCCGCCCGGCGGCTTGCCGGCACCCACCC
GCAGCCTGTAGCCTGCCGGCCCAAGCATGAGGGAAGCGGCATGTACATCTACCGACTGGT
CCTGCTCCTGGTCGTGGGCATCTATCTCTTTTCCCCCGCCATCATGGACTGGTGGATCGA
CCCCAACGGCGCCTGGTACCGCCCGTACCTGCTGTGGCTGATCCTCATCGTGGTCACTTT
CATCCTGCAGAGCCAGCGCG**ATG**CTGACGAGCTTTAGCCTGACCCAGCTGATCCTGATCA
GCGTCACCTACCTCTCCACCCTCTTCGGCATCGCCTGGATCACCGAGCGCGGCTACGTGC
CGCGCCGGCTGGTACGCCACCCGCTGGTCTACACCCTGTCGCTGGGCGTCTACGCCAGTG
CCTGGGCGTTCTACGGCACGGTAGGCCTGGCCTACCAGTACGGCTACGGGTTCCTCGCCA
TCTACCTGGGGATTTCCGGGGCCTTCCTGCTCGCCCCGGTGCTGCTCTATCCGATCCTGC
GGGTGACACGCGCCTACCAGCTGTCCTCGCTGGCCGACCTCTTCGCCTTCCGTTTCCGCA
GTACCTGGGCCGGCGCCCTGACCACGCTGTTCATGCTGATCGGCGTGCTGCCGATGCTGG
CCCTGCAGATCCAGGCGGTCACCGACTCGATCAGCATTCTCACCCGCGACCCGGCCCAGG
AGCGGGTCGCATTCGTCTTCTGCACCCTGATCACCCTGTTCGCGATCCTCTTCGGCGCTC
GCCACATCGCCACCCGCGAACGCCACGAGGGACTGGTGTTCGCCATCGCCTTCGAGTCGC
TGGTCAAGCTGGTGATGCTCGGCAGTATCGGCCTCTACGCCCTGTACGGCGTCTTCGGCG
GGCCGGAAGGCCTGGAAGTCTGGCTGCTGCAGAACCAGACCGCGCTGACCACCCTGCACA
CGCCGCTGGCCGAAGGTCCATGGCGAACGCTGCTGCTGGTGTTCTTCGCCGCGGCCATCG
TGATGCCGCACATGTTCCACATGATCTTCACCGAGAACCTCAACCCGCGCGCGCTGATTT
CCGCCAGTTGGGGCCTGCCGCTGTTCCTGCTGCTGATGAGCCTGGCGGTACCGCCGATCC
TCTGGGCCGGGCTGCGCCTCGGCGCCTCGACCACACCGGAGTACTACACCATCGGCCTCG
GCCTGGCGGTGGACAGCCCGGCCCTGGCCCTGGCCGCCTTCATCGGCGGCATCTCCGCCG
CCAGCGGGCTGACCATCGTGATGACCCTGGCGCTCTCGGGAATGGTCCTCAACCACCTGG
TGCTGCCGCTCTACCAGCCGCCGGCGCAGGGCAACATCTACCGCTGGCTGAAATGGACCC

GGCGCCTGCTGATCGTCGCCATCATCATGGCCAGCTACGCCTTCTACCTGCTGCTGGGCG
CCGAACAGGACCTCTCCAACCTGGGCATAGTGTCCTTCGTCGCCACCCTGCAATTCCTTC
CCGGCGCCCTCTCGGTGCTGTACTGGCCGACCGCCAACCGCCGTGGCTTCATCTCCGGGC
TGATCGCCGGCATGCTGGTCTGGGCGATCACCATGCTGCTGCCCCTGATGGGGAACGTAC
AGGGCCTGTACCTGCCGCTGTTCGACGTGATCTACGTGCTGGACGATTCGAACTGGCACC
TGGCGGCGCTGTCCTCGCTGGCGGCCAACGTGCTGGTATTCACCCTGGTGTCGCTGTTCA
CCGAGGCCAGCGACGAGGAAAAAGGCGCCGCCGAGGCCTGCGCGGTGGACAACGTACGCC
GTCCGCAACGCCGCGAACTGTTCGCCGGTTCGCCGCAGGAGTTCGCCAGCCAGTTGGCCA
AGCCGCTGGGCGCCAAGACCGCGCAGAAGGAGGTCGAGCAGGCCCTGCGCGATCTCCACC
TGCCCTTCGACGAACGTCGCCCCTATGCCCTGCGTCGCTTGCGCGACCGCATCGAGGCGA
ACCTTTCCGGGCTGATGGGACCCAGTGTCGCCCAGGACATCGTGGAGACCTTCCTTCCCT
ACAAGACCAGCGAAGAAAGCTACGTCACCGAGGACATCCACTTCATCGAGAGCCGCCTGG
AGGACTATCACTCCCGGCTGACTGGCCTGGCTGCCGAACTCGACACCTTGCGCCGCTACC
ACCGGCAGACCCTGCAAGACCTGCCGATGGGCGTCTGCTCCCTGGCCAAGGACCAGGAAG
TGCTGATGTGGAACCGCGCCATCGAGGAACTCACCGGGGTCGGCGCGCAGAAGGTCGTCG
GTTCGCGCCTGTCGGCCTTGCCCGAACCCTGGAAGGGCCTGCTGGAACGTTTCATCGACG
CCCCCGACGAGCACCTGCACAAGCAGCGCCTGGTCTACGACGGCCACACCCGCTGGCTGA
ACCTGCACAAGGCGGCCATCGAGGAACCCCTGGCGCCGGGCAACAGCGGCCTGGTGCTAC
TGGTGGAAGACCTCACCGAAACCCAACTGTTGGAAGACAAGCTGGTCCACTCCGAGCGCC
TGGCTTCCATCGGCCGCCTAGCCGCCGGGGTGGCCCACGAGATCGGCAATCCGATCACCG
GCATCGCCTGCCTGGCGCAGAACCTGCGCGAGGAACGCGAAGGCGACGGCGAGCTGACCG
AAATCAGCGAGCAGATCCTCGACCAGACCAAGCGCGTATCGCGCATCGTCCAGTCGTTGA
TGAGCTTCGCCCACTCCGGCAGCCACCTGCAGGCCCTGGAACCGGTGTGCCTGAGCGAAG
TCGCCCAGGAGGCCATCGGACTGCTGTCGCTGAACCGGCGTAGCGTCGAAGTAGAGTTCT
TCAACCTCTGCGATCCGGCGCACTGGGTCGAGGGCGACTCGCAGCGCCTGGCCCAGGTGC
TGATCAACCTGCTCTCCAATGCTCGCGACGCCAGCCCGCCGGGCGGCGCGATCCGGGTCC
GCAGCGAAGCCTCGGAACATACCGTGGACCTGGTCGTCGAGGACGAGGGCAGCGGCATTC
CGAAGGCGATCATGGACCAGTTGTTCGAACCGTTCTTCACCACCAAGGACCCCGGCAAGG
GGACCGGGCTCGGCCTCGCACTGGTCTATTCGATCGTGGAAGAGCATTATGGACAGATAA
CCATCGAGAGCCCGACGGATCACCAGCGAGAAGGCGGCACCCGCTTCCGCGTGACCCTGC
CGCGGCACCCTGGCCCGACGGCCGAGCTG**TAG**ACGAGCACGTCGAGAGAGCTGAATACAT
GGCACATATTCTGATCGTCGAAGACGAAACCATTATCCGATCTGCATTGCGACGCCTGCT
GGAGCGCAACCAGTACCAGGTCAGCGAAGCCGGCTCGGTGCAGGAAGCCCAGGAGCGCTA
CACCATCCCCTCCTTCGACATGGTGGTCAGCGACCTGCGCCTGCCGGGCGCCCCCGGCAC
CGAACTGATCAAGCTCGCCGAAGGCATTCCGGTGCTGATCAT

> cbrB (G3XCK7)
GAACCGGTGTGCCTGAGCGAAGTCGCCCAGGAGGCCATCGGACTGCTGTCGCTGAACCGG
CGTAGCGTCGAAGTAGAGTTCTTCAACCTCTGCGATCCGGCGCACTGGGTCGAGGGCGAC
TCGCAGCGCCTGGCCCAGGTGCTGATCAACCTGCTCTCCAATGCTCGCGACGCCAGCCCG
CCGGGCGGCGCGATCCGGGTCCGCAGCGAAGCCTCGGAACATACCGTGGACCTGGTCGTC
GAGGACGAGGGCAGCGGCATTCCGAAGGCGATCATGGACCAGTTGTTCGAACCGTTCTTC
ACCACCAAGGACCCCGGCAAGGGGACCGGGCTCGGCCTCGCACTGGTCTATTCGATCGTG
GAAGAGCATTATGGACAGATAACCATCGAGAGCCCGACGGATCACCAGCGAGAAGGCGGC
ACCCGCTTCCGCGTGACCCTGCCGCGGCACCCTGGCCCGACGGCCGAGCTGTAGACGAGC
ACGTCGAGAGAGCTGAATAC**ATG**GCACATATTCTGATCGTCGAAGACGAAACCATTATCC
GATCTGCATTGCGACGCCTGCTGGAGCGCAACCAGTACCAGGTCAGCGAAGCCGGCTCGG
TGCAGGAAGCCCAGGAGCGCTACACCATCCCCTCCTTCGACATGGTGGTCAGCGACCTGC
GCCTGCCGGGCGCCCCCGGCACCGAACTGATCAAGCTCGCCGAAGGCATTCCGGTGCTGA
TCATGACCAGCTACGCCAGCCTGCGTTCGGCGGTGGATTCGATGAAGATGGGCGCGGTGG
ACTACATCGCCAAGCCTTTCGACCACGACGAAATGCTCCAGGCCGTGGCGCGCATCCTCA
AGGACCGCCAGGAGAACCGCAGCGCGGCCCCGGTCTCCGCCAACGGCGGCAAGGCCGGCG
GCGAGCGCGGCGCCAGCCCGGCGGTAGCCGACGGCGAGATAGGCATCATCGGCTCCTGTG
CGCCGATGCAAGAGCTGTACAGCAAGATTCGCAAGGTCGCGCCGACCGACTCCACGGTAC
TGATCCAGGGCGAGTCCGGCACCGGCAAGGAACTGGTCGCCCGCGCCCTGCACAACCTGT
CGAAGCGCGCCAAGGCGCCGCTGATCTCGGTGAACTGTGCGGCGATTCCGGAAACCCTGA
TCGAATCCGAGCTGTTCGGCCATGAAAAAGGCGCCTTCACCGGTGCCAGCGCCGGGCGCG
CCGGGCTGGTCGAAGCGGCCGACGGCGGCACCCTGTTCCTCGACGAGATCGGCGAACTGC
CGCTGGAGGCCCAGGCCCGCCTGCTGCGCGTATTGCAGGAAGGCGAGATCCGCCGGGTCG
GCTCGGTGCAATCGCAGAAGGTCGACGTGCGCCTGATCGCCGCCACCCACCGCGACCTGA
AAACCCTGGCCAAGACCGGGCAATTCCGCGAAGACCTCTATTACCGCCTGCATGTGATCT

CGCTGAAGCTGCCCGCCCTGCGCGAGCGTGGCAACGACGTGATGGAGATCGCCCGCGCCT
TTCTGGCTCGCCAGTGCACCCGGATGGGGCGCGCGCCGCTCAGCTTCGCCCACGACGCCG
AACAGGCGATCCGCCATTACCCATGGCCGGGCAACGTGCGCGAACTGGAAAACGCCATCG
AGCGGGCGGTGATCCTCAGCGAGAGCCAGGAAATCCACGCCGACCTGCTGGGTATCGACA
TCGAGCTGGACGACCTCGAGGACGACTTCGCCGCCGACCTCGGCCTCGGCCCGGCGGGCG
CCGCGGCCAGCAACCACGAGCCCACCGAGGACCTTTCGCTGGAAGACTACTTCCAGCACT
TCGTCCTCGAGCACCAGGACCACATGACCGAGACCGAACTGGCCCGCAAGCTGGGCATCA
GCCGCAAATGCCTGTGGGAACGCCGCCAGCGCCTGGGCATTCCGCGGCGTAAATCAGGGG
CCTCGGCCGACTCG**TAA**CACCCTGCAACCTGTTACCGCGGACGTACCGCGTAACAGAAAC
CGGGTTCATCGGTAACGGGAACCCGGTTTTTTTGTGCCCTCCGCCGACCCACCGAAAACC
TCAACCCATTGATTTTACTGGGTTTCGAAAAACTGGCACGGCATCTGCTTATGTATTGGC
ACAACAACAATAACAAGCAACGACGAAGACAATAAAAACAACACGTAACGACTCCAGCAC
AACAAAAACAAAATCGCGGAGGCGCAG

> retS (Q9HUV7)
CATGGTCCGCCTGGAGTCCTCCCTGGTCCTGCTGGTCGAGGCCGCCCTGGCCAAGGCGCT
GGACAAGGTCGAGGCGACCTGGGACCCGCGTCCGACCGTGGGCGTGGTACTGGCCGCCGG
CGGCTATCCGGGCGACTACGCCAAGGGCGAGGTCATCGAAGGCCTGGCCGAGGCCGCCGC
GCTGGACGGCAAGGTGTTCCACGCCGGCACCGCGCTGAAGGATGGCCAGGTGGTCACCTC
CGGCGGCCGCGTGCTCTGTGCCACCGCCATCGGCGAGAGCGTGTCCGCCGCCCAGCAACA
GGCCTATCGCCTGGCCGAGAAGATCCGCTGGAACGGCTGCTTCTACCGCAAGGACATCGG
CTACCGTGCCATCGCCCGCGAGCGCGGCGAGTCCTGACCGGGCCCGAAGCCGCGGGCGCC
GCACGGCGCTTCCGCGGCCTATGCCGTTGCCACGGCCACTTGGCTATAATCCGGCCATTC
ACCTTCGAAGGGACTTCGCC**GTG**GTACGGCTTCGGATCGCCATAGGACTACTCGTCAGCT
TCCTGCTGCTTCTGCTCGGTCCGATGTCGCCGGCGGTCGCGGACGACGCCGGCGTTTCCA
GCGTTCCCCTGCAAACCACCGCCACTACTCCCAGCGCCAACCAGAACTGGCGCCTGCTGC
GCGACGAGAGCGCGCAACTGCGGATCGCCGACGTCCTCCAGCGCAAGGAGCAGTTCCGCC
CGCTGGCCAAGCGCTCCTTCATCTTCCCCGCCAGCCCCCAGGCGGTCTGGCTGCAGGTCC
AGCTTCCCGCGCAGAAGGTGCCGAGCTGGCTGTGGATCTTCGCCCCACGGGTGCAGTACC
TGGACTACTACCTGGTGCAGGACGGCCAACTGGTGCGGGACCAGCACACCGGCGAGTCGC
GACCGTTCCAGGAGCGCCCGCTGCCCTCGCGCTCCTACCTGTTCTCGCTGCCGGTCGACG
GCAAGCCGATGACCCTGTACGTGCGGATGACCTCCAACCATCCGCTGATGGCCTGGTTCG
ACCAGATCGACGAAGCCGGCCTGGTCGGCCTGGAGAAGCCCGCCTACGCCTTCGGCATGC
TGCTCGGCGGCATGCTCCTGCTGCTGATGTACAACCTGATCCGCTTCGCCTACTCGCGCA
GCGCCAGCAGCCTGTGGCTGGCGGCGGTGCACGCGGCGCTGGCGGTCTGCGCCGCGGCGA
ACCTGGGGCTGGTGGCGTTCTGGCTGCCGGGACTCAAGTTCAACCAGTCGCTGACCGCCG
ACCTCGGCGCCCTCGGCGCCGCGGTGAGCCTGCTGTGGTTCGCCTGCAGCTTCTTCCGCG
GCACTGCGGAAAGCCGCCTGAACCGCCTCCTGCAAGGCGAGGCGCTGTTGATCCTGGCGG
TCGGCGCGATCATCGCCTTCACCCAGCAACTCTGGTTCAGTTGGCTGATCTACCTGCTGG
TGATCCTCAGTTCGCTCAGCGTCCCGCTGATCGCGGCCTGGCACTGGTATCGCGGCTACC
AGCCGGCGCGCCTGATCGTTGCCGGGATGATCGTGTTCAACGCCGGCTTCATGGTGTTCC
TGCCGGTACTGTTCGGCACCAAGCAACTCGATCCGGGCTGGCTGGTGCTCGGCGTGTTCA
GCTTCGCCACCCTCGCCGGGCTGGTCCTGAGCGTCTCGCTCACCGAACGCCAGCGGCTGA
TCCAGCAGCTCAACCTGCAACAGCGCACCAGCGAGGCGGCGCACACCGCGGAATTGCAGA
CCAAGGCCGAGTTCCTGGCCAAGATCAGCCACGAGATCCGCACGCCCATGAACGGCGTGC
TGGGCATGACCGAACTGCTCCTCGGCACCCCGTTGTCGGCGAAGCAGCGCGACTACGTGC
AGACCATCCACAGCGCCGGCAACGAGCTGCTCACGCTGATCAACGAGATACTCGACATCT
CCAAGCTGGAGTCCGGGCAGATCGAGCTGGACGAAGTGCAGTTCGACCTCAACGCGTTGA
TCGAGGACTGCCTGGATATCTTCCGGGTCAAGGCCGAGCAGCAGCGCATCGAGCTGATCA
GCTTCACCCAGCCGCAGGTGCCGCGGGTCATCGGCGGCGATCCGACGCGCCTGCGCCAGG
TGGTCCTGAGCCTGCTGGACAACGCGTTCAAGCAGACCGAGGAAGGCGAGATCCTGCTGG
TGGTGGCCCTCGACGACCAGGGCGAGACGCCACGCCTGCGCATCGCCGTGCAGGACAGCG
GCCACCCGTTCGACGCCAAGGAGCGCGAGGCGCTGCTCACCGCCGAGCTGCACAGCGGCG
ACTTCCTTTCCGCCAGCAAGCTCGGCAGCCACCTCGGCCTGATCATCGCCCGCCAACTGG
TGCGGCTGATGGGCGGGGAGTTCGGCATCCAGTCCGGCAGCAGCCAGGGCACCACCCTGT
CGCTGACCCTGCCGCTCGACCCGCAGCAGCTGGAGAACCCCACCGCCGACCTCGACGGCC
CGCTGCAGGGCGCCCGGCTGCTGGTGGTGGACGACAACGAAACCTGCCGCAAGGTGCTGG
TGCAGCAGTGCAGCGGCTGGGGCCTGAACGTCAGCGCGGTGTCGTCCGGCAAGGAGGCGC
TGGCCCAGTTGCGCACCAAGGCGCACCTGCGCGAGTACTTCGACGTGGTCCTGCTCGACC
AGGACATGCCCGGCATGACCGGCATGCAACTGGCGGCGAAGATCAAGGAAGACCCCAACC
TCAACCACGACATCCTGCTGATCATGCTCACCGGGATCAGCAACGCGCCGAGCAAGATCA

TCGCCCGCAACGCCGGGATCAAGCGCATCCTGGCCAAGCCGGTGGCCGGCTACACGCTGA
AGGCGACCCTCGCCGACGAACTCGCCCAGCGCGGCGTCAGCGGCGTCACCAACTACCTGC
AACCGGCCAAGGAAGCGCAGGCGCCGAGCCTGCCGAGCGACTTCCGGATCCTCGTCGCCG
AGGACAACAGCATCTCCACCAAGGTCATCCGCGGCATGTTGAACAAGCTCAACCTCCAGC
CGGACACCGCCAGCAACGGCCAGGAGGCGCTCAGCGCGATGAAGGCGACCCAGTACGACC
TGGTGCTGATGGACTGCGAGATGCCGGTTCTGGACGGCTTCTCCGCCACCGAGCAGCTCC
GCGCCTGGGAAGCCCACGAACAGCGCCCGCATACCCCGGTGGTGGCGCTCACCGCGCACA
TCCTCAGCGAACACAAGGAACGCGCGCGGCTGGTCGGCATGGACGGGCACATGGCCAAGC
CGGTGGAGCTGTCGCAATTGCGTGAGCTGATCGCCTACTGGGTCGGCGAACGCGATCGCC
GCCGCCAGGGCGACGCCCTGCCCTCC**TGA**GGGCAGCGACGTGCTCCGGCGCTGCACCCGG
CCTCCGCCACGGCGGATAACCGCACGCGGCTATTCGCCCTACGCCCCGGTGGTCCCGGCT
CCCGTAGGGCGCATGACGCCAACGGCGTTATCCGCCGATACCGCCACTTCCGCCACGGCG
GATAACCGCACGCGGTTATTCGCCCTACGCCCCGGTGGTCCCGCCTCCCGTAGGGCGCAT
AACGCCAACGGCGTTATCCGCCGATACCGCCACTTCCGC

> parC(Q9HUK1)
ACGGATCATGCTGGTGCTGGCCTGGATCGCCGGGCTGGCGCTGGCGACGCGCTATTTCGG
CGTCTGGGAAGATCGACAGCGCAACCCGAACCAGGCGCCGCAATCGATCCATGGCGACGG
CTACGTCGAGTTGCGCCTGGCCAGCAGCCGGCAGGGCCATTACCTGCTGAACGGGCAGAT
CAACGGCCAGGGCGTGACCTTCCTGCTCGATACCGGGGCGACCCAGGTGGCGGTGCCGGA
AGCCCTGGCGGCAAGACTCGCGCTGGAGCGCGGCGCGCCGATCACCCTGAGCACGGCCAA
CGGCCGCGCCACGGGCTGGCGCACGCGGCTGGACCAGCTGCAACTCGGCGATATCCGCCT
GTCCGGGGTCGCGGCGCTGATTGCGCCGGGCATGGACGGCGATGAGGTGCTACTCGGCAT
GAGCGCCCTGAAACAACTCGAATTCACCCAGCGCGACGGCACCCTGGTGCTGCGCCAAAA
CACTTCTCCGTGAGGTACGC**ATG**AGCGAATCCCTCGATCTGAGCCTGGAAGGGGTCGAAC
GCCGGTCGTTGGCCGAGTTCACCGAGCAGGCCTATCTGAACTATTCCATGTACGTGATCA
TGGACCGCGCCCTGCCGCATATCGGCGACGGCCTGAAACCGGTGCAGCGACGCATCGTCT
ACGCCATGAGCGAACTGGGGCTGGATGCCGATTCCAAGCACAAGAAGTCGGCGCGCACCG
TCGGCGACGTGCTCGGCAAGTTCCACCCGCACGGCGACTCGGCCTGCTACGAGGCCATGG
TGCTGATGGCGCAGCCGTTCTCCTATCGCTATCCGCTGGTGGACGGCCAGGGCAACTGGG
GGGCTCCGGACGATCCCAAGTCCTTCGCCGCCATGCGTTATACCGAGGCGCGCCTGTCGC
GCTATTCCGAGGTGCTGCTCAGCGAACTGGGCCAGGGTACCGTGGACTGGGTACCGAACT
TCGACGGCACCCTCGACGAGCCGGCCGTGCTGCCGGCCCGCCTGCCCAACCTGCTGCTCA
ACGGCACCACCGGCATCGCGGTGGGCATGGCCACCGACGTGCCGCCGCACAACCTGCGGG
AAGTCGCGTCGGCCTGCGTGCGCCTGCTCGACCAGCCGGGCGCGACGGTCGCCGAATTGT
GCGAACACGTGCCGGGCCCGGACTTCCCCACCGAAGCCGAGATCATCACCCCGCGCGCCG
ACCTGCAGAAGGTCTACGAGACCGGCCGCGGTTCGGTGCGCATGCGCGCGGTGTACCGCG
TCGAGGACGGCGATATCGTCATCCACGCCCTGCCGCACCAGGTGTCCGGTTCCAAGGTGC
TGGAACAGATCGCCGGGCAGATGCAGGCCAAGAAGCTGCCGATGGTGGCCGACCTGCGCG
ACGAGTCGGACCACGAGAACCCGACCCGCATCGTCATCATCCCGCGTTCGAACCGGGTCG
ATGTCGAAGAGCTGATGACCCATCTGTTCGCCACCACCGACCTGGAGACCAGCTACCGGG
TCAACCTGAACATCATCGGCCTCGACGGCAAGCCGCAGGTCAAGGACCTGCGCCAGTTGC
TCTCGGAGTGGCTGCAGTTCCGCATCGGCACCGTGCGTCGACGCCTGCAGTTCCGCCTGG
ACAAGGTCGAGCGCCGCCTGCATCTGCTGGATGGCTTGCTGATCGCCTTCCTCAACCTCG
ACGAGGTGATCCACATCATCCGCACCGAGGACCAGCCCAAGGCGGTGCTGATGGAGCGCT
TCGAACTCAGCGAGGTGCAGGCCGACTACATCCTCGACACCCGCCTGCGCCAGTTGGCAC
GCCTGGAAGAGATGAAGATCCGCGGCGAGCAGGAAGAGTTGCTGAAGGAGCAGAAGCGCC
TGCAGACCCTGCTCGGCAGCGAGGCCAAGCTGAAGAAGCTGGTGCGCGAGGAGCTGATCA
AGGACGCCGAGACCTACGGCGACGACCGCCGTTCGCCGATCGTCGCCCGCGCCGAGGCCC
GCGCGCTGTCGGAAACCGAGCTGATGCCCACCGAACCGGTGACCGTGGTGCTCTCGGAAA
AAGGCTGGGTGCGTTGCGCCAAGGGCCACGACATCGACGCCGCCGGCCTCTCCTACAAGG
CCGGCGACGGCTTCAAGGCCGCCGCGCCGGGACGCTCGAACCAGTATGCGGTGTTCATCG
ACTCCACCGGGCGCAGCTACTCGCTGCCGGCCCACAGCCTGCCGTCCGCGCGAGGCCAGG
GCGAGCCACTCAGCGGCCGGCTGACGCCGCCGCCGGGGGCCAGCTTCGAATGCGTGCTGC
TGCCGGACGACGATGCGCTGTTCGTGATCGCTTCCGACGCCGGCTATGGTTTCGTGGTCA
AGGGCGAGGACCTGCAGGCCAAGAACAAGGCCGGCAAGGCCCTGCTCAGCCTGCCCAACG
GCTCCGCCGTGGTGGCGCCGCGCCCGGTGCGCGATGTGGAGCAGGATTGGCTGGCGGCCG
TGACGACCGAGGGCCGTCTGCTATTGTTCAAGGTCTCCGACCTGCCGCAGCTCGGCAAGG
GCAAGGGCAACAAGATCATCGGCATCCCCGGCGAACGCGTGGCCAGCCGCGAGGAATACC
TCACCGACCTGGCTGTTCTGCCAGCCGGGGCGACGTTGGTCCTGCAGGCCGGAAAGCGTA
CCCTGTCGCTCAAGGGCGACGACCTGGAACACTACAAGGGGGAGCGAGGCCGGCGAGGCA

ACAAGCTGCCGCGCGGTTTCCAGCGCGTCGACAGCCTGCTGGTGGATATTCCGCCACAGG
AT**TGA**AATGAGCCGTTACCGGTCTCCGATCTCTCGCATATCCGCAAGGGAATGGCGAAAG
CGGCCTCTTCCGAGGCCGGCTTTCCGGGGGAATGTCGTCTCCCTGGCGGGTTCCGCCGGG
TTTTCCCCTGACGCAGGTGGTCCGAAACCGGTTTCGGAATACGCTCTGCCTCTGGAGCTA
TGGCGCTGGTTAGGGGATGATACGCCCCTTCGGCCGCCGTTTCCGATGGCGGGTGGCCAG
TAGAATTCAAGTCTT

> pilE (G3XD43)
CCGGTACCGAGCAGAAAGGCCTGGGAGGCTTGACGCTGAGCACCAACGAACAGGGCAATC
CGGAAGTCTGCTCCTCGGGCGAATGCCTGACCGTGAACCCCGGTCCGAACACCCGTGGCC
GCCAGAACTGGCGCCCCATCGAAGGAAAGAACTGATATGAAAGTGCTGCCTATGCTGCTG
GCCCTGGCCGTTCCCGGCCTCTGCTGGGCCGAAGATCCCCAGACCTTCGAAGGCGCTGGC
GTGGTCTTCGAAGTCCAGGTCGAGAAGAACCTGGTGGACATCGACCACCGGCTCTACCGC
CTGCCCAACAGCACCGTCAGGAACGGCATGCCCAGCCTGTTCCAGGTCAAGCCGGGCTCC
GTGGTGTCCTACTCTGGCACCGTCAGCCAGCCGTGGTCCACCATCACCGATATCTACATT
CACAAACAGATGAGCGAACAGGAGCTGGCCGAGATGATCGAGAAAGAACAGCCCCGCCAG
GACGGGGAGGAGCAGCCCCG**ATG**AGGACAAGACAGAAGGGCTTCACGTTGCTGGAAATGG
TGGTGGTAGTGGCGGTGATCGGCATCCTCCTCGGCATCGCCATTCCCAGTTACCAGAACT
ACGTGATCCGCTCCAACCGCACCGAGGGCCAGGCCCTGCTCTCGGACGCGGCCGCGCGCC
AGGAACGCTACTACTCGCAGAACCCCGGGGTCGGCTACACCAAGGACGTGGCCAAGCTGG
GCATGAGTTCGGCCAACTCGCCGAACAACCTGTACAACCTCACCATAGCGACGCCCACCA
GCACCACCTATACCCTGACCGCCACGCCGATCAACTCGCAGACCCGCGACAAGACCTGCG
GCAAGCTGACCCTCAATCAGCTCGGCGAACGCGGCGCAGCCGGCAAGACCGGCAACAACA
GCACCGTCAACGACTGCTGGCGC**TGA**AACGAAAGAGCCCCTCTACGAGAGGGGCTCTTTC
ATTGCGCACGGTTCACAAAGCCTTGACCCGCAGTTCCTTGGGCATCGAGAAGGTAATGTT
CTCCTCCCGCCCCTCCAGTTCCTGCTCCTCCGACGCCCCCCACTCGCGTAGCTGGGCGAT
CACGCCGCGCACCAGCACTTCCGGCGCGGAAGCGCCTGCGGTGATTCCGATGCGACGCAC
ACCGTCGAACCAGCCGCGTTGCATGTCCTCGGCGCC

> recQ (Q9HYQ1)
ATCGCGCTTTCGTCGCGGTGCTCCTAATGGGTAGGGCATCGGAAGTCGACTTCAGGGAGC
GATCGAATGTCCGCGCCGCATCTCGATGATCGTGTTCTGGCTTCGCTGCAGGAGGTCATG
GAGGACGAATATCCGGTCCTGCTGGATACCTTCGTGCTCGACTCCGAGGAGCGCCTGCGC
AGCCTGCATGCCGCCCTCCAGGCCGGCGATGCCCAGGCTTTGCGGCATACCGCACACAGC
TTCAAGGGAGGCAGCAGCAACATGGGCGCGGTACTCCTCGCCGGCTACTGCAAGGAGCTG
GAGGAAAGCGCCAGGCGCGGCGAGCTGCAACGGGCGCCGGCGCTGATCGAACAGATGGAG
CGCGAATTCGCCATCGTCCGCATCCTTTTCAAACAGGAACGTCAGCGCTATCGCTGAGGT
TTTCGTCCTTTCCGGCGACAAGGTACCGGCCCGATCGAGCCTCCCTGGCGTATAATGCGC
CGCTCGATTTGGAGTCCTTC**ATG**CGCGAGCAAGCCCTACGCATCCTCAAAGACGTTTTCG
GTTACGACGCCTTCCGTGGCAACCAGGCACGGATCATCGAGCGGGTAGCCGAGGGTGGCG
ATGCGCTGGTACTGATGCCCACCGGCGGTGGCAAGTCCCTGTGTTTCCAGGTCCCGGCGC
TGCTGCGCGAAGGCCTGACGGTGGTGGTGTCGCCGCTGATCGCGCTGATGGAGGACCAGG
TCGCCACCCTGGACGAACTCGGCGTGCCGGCGGTGGCGCTGAACTCCACCCTCAACCCCG
AGCAGCAGCGGGACATCGCCGAGCGCCTGCAGCGTGGCGAGATCAAGCTGCTCTACCTGG
CACCGGAACGGTTGGTCCAGCCGCGCATGCTGGCCTTCCTGCAACGACTGCCGGTCGGCC
TGTTCGCCATCGACGAAGCGCACTGCGTGTCGCAATGGGGCCATGACTTCCGTCCCGAAT
ACCTGCAGCTCGGCCAGCTTGCCGAGCTGTTCCCGCAGGTGCCGCGGATCGCCCTGACCG
CCACCGCGGACATGCGTACCCGCGAGGAGATGATCCAGCGCCTGCACCTGCAGAACGCCG
AGCAGTTCCTCTCCAGCTTCGACCGGCCGAACATCTTCTACCGCATCGTGCCCAAGGAGC
AGCCGCGCAAGCAGTTGCTCGGCTTCCTCTCCGAGCGGCGCGGCGATGCCGGCATCGTCT
ACTGCCTGTCGCGGAAGAAGGTCGAGGAGGTCGCGGAATTCCTCGGCAACCAGGGCTTCC
CCGCGCTGCCGTATCACGCCGGACTGTCCAACGAACTGCGTGCCCACCACCAGAAGCGCT
TCCTCAACGAGGAAGGCTTGATCATGGTGGCGACCATCGCCTTCGGCATGGGCATCGACA
AGCCCAACGTGCGTTTCGTCGCCCACCTCGACCTGCCCAAGAGCCTCGAGGCCTATTACC
AGGAAACCGGCCGCGCCGGCCGCGATGGCCTGCCGGCCGACGCCTGGATGGCCTACGGCC
TGCAGGACGTCCTGCTGCTGCGGCAGATGATGCAGAGTTCCGAGGGCGACGAGCGGCACA
AGCGCGTCGAGCGGCACAAGCTGGAAGCCATGCTGGCGCTCTGCGAGGAAACCCGTTGCC
GGCGCCAGGCGCTGCTGGCCTATTTCGACGAGGAGATGCCGCAGCCCTGCGGGCATTGCG
ACAACTGCGTGGACGGCGTGGAAACCTGGGATGCCACCGAATCCGCGCGCCAGGCGCTGT
CGGCGATCTACCGCAGCGGCCAGCGCTACGGCGTCGGCCATCTGGTGGATATCCTGCTCG
GCCGCGAGACCGAGAAGATCCGCAGTCTCGGCCACCAGCACCTGGCGGTGTTCGGGATCG

GCAAGGGGCGCGGCGAAGACGAGTGGCGGACCCTGTTCCGCCAGTTGGTCGCGCGCGGCC
TGGCCGACGTCGACCTGGACGGCTTCGGCGGCCTGCGCCTGACCGAGGCCTGTCGTCCGC
TGCTGCGGGGCGAGGTGCGGCTGGAGCTGCGCCGCGACCTCAAACCACAGCGCGCCAAGG
GCTCCTCCAGCGGCGGCGCCAGCGCCGCCAGCCAATTGGTGCGCAGCGAAGAGCGGGAAA
TGTGGGAGGCCCTGCGCGCGCTGCGGCGCAAGCTGGCCGAAGAGCATTCGGTGCCGCCCT
ATGTGATCTTCCCCGACGCGACCCTGCTGGAAATGCTCCGCAGCCAGCCGCGCTCGCTAT
CCGACATGGCCCAGGTCAGCGGGGTCGGCGCGCGCAAGCTGGAGCGCTACGGCCAGGCCT
TCCTCGATGTCCTCACCGACTCGCCGGCGGCGCCCGCCGCGCCGCCGCAGGACCTGCGCC
ACGAATTGGCCAGCCTGGCCTGTGCGGGGATGACCCCGGCGCAGATAGCCCGCCAGCTCA
ATTGCAGCGAGAAGAACGTCTACGCGATGCTCGCCGAGGCCATCGCCGGTCAGCAGGTGA
GCCTGGAGCAGGCGCTGGATCTGCCCGAGGAACTCCTCGGCGAAATCCAGGACGCCTTCC
TCGAGGAAGACGGCGAACTGCCGCCGGTGGCGGCGCTGGAGGAGCGTTTCGGCAAGCGGG
TGCCGAGTGGCGTGCTGCACTGCGTGCGCGCCGCCCTGCAGGTGGAACTGGAGTCG**TGA**T
ATGGCCATTTGCCGGTATTTTCTGACGCCAATCTTGTGACGTCCTTCGCAAGCGTCTACT
GTGACGCCAAGCCCTCCAAGAGAAGAGGTGTGCGTGACACAGAAGGACCGGTTGGACGAC
TCCCGTTCCAATCTCTATGCCGACCAATTGAACCGCGGTTTCCGCGGTCTGCGTTTCCTT
CCCGAACTGGAGCGTGACTACCGGCGCTACATGCTGGAGGACAGCTTCGCGCTGAAACGC
ATCGCCCTC

> pvrR (A0A0H2ZH80)
GAGCCGCAGGCACAGGGCCGCGACTGGCTGCTCGGGCTCAACAACCTGGACGGCCTGCAT
CGTGCTCTGGGCCTGGCCCATGGGCGTCTCGCTGATCCTTCGACGCCGCCGATACGGCTG
GCTCCGTTGCGCAATCTAGGTCTCCGCGTCCTAGTGGTGGAGGATAACGCGATCAACCAG
TTGATCTTGAGGGACCAGATGGAAGCGCTGGGCTGCAGCGTGGAGCTGCTCTTCGATGGT
CGCGAGGCGTTGCTGCACTGCCAGACGGCCTGCTTCGACGTGGTGCTCACCGATATCAAC
ATGCCGAACATGAACGGATACGAGCTAACCGCGGAGCTACGGCGCCAAGGGTTCCGGCAG
CCGATCATCGGCGCGACGGCGAACGCCATGCGTGAGGAGCGCGAGCGCTGCATGTCCGCC
GGGATGAACGATTGCCTGGTCAAACCGGTGGATCTGAATGCCCTTCAGAACTGCTTGATT
AATATTCTCAAGGTGGATCG**ATG**AGCTGGAAATCCTATCGGGTGCTGGTGGTCGAAGATC
AGCCGTTTCAGCGCGAATACCTGCTCAACCTGTTTCGCGAGCGCGGCGTGCAGTACCTGG
TAGGTGCCGGCGACGGCGCGGAGGCGTTGCGCTGCCTGAAGCAGGACAGGTTCGACCTGA
TCCTCAGCGATCTGATGATGCCGGGCATGGATGGTATCCAAATGATCCTGCAACTGCCGT
ATCTCAAGCATCGTCCGAAGCTGGCGCTGATGAGCTCCTCGTCGCAGCGGATGATGCTCA
GTGCCAGCCGGGTCGCCCAGAGTCTCGGCTTGTCGGTAATCGACCTGTTGCCCAAGCCGA
CTCTGCCCAAGGCCATCGGCCAACTTCTGGAACACCTGGAAAGATGCCTCAGGCAGAAGC
TGGAGCCGGAAACCGACGAGACTCCGCATGGGCGCACGGCGTTGCTGGATGCCCTGCATA
ACGAGCAACTGGTGACCTGGTTCCAGGCTAAGAAATCCCTCCACACCGGGCGCATAGTCG
GCGCCGAGGCGTTGATACGCTGGAGCCACCCGCAGCATGGCCTGTTGCTGCCCAGCTGTT
TCATGAGTGATGTCGACGCTACCGGTCTGCACGAGGCGTTGCTCTGGCGCGTGCTCGAAC
AGACCCTGAACGCCCAGGAATCGTGGCGCAGGGCGGGTTACGAGATTCCGGTTTCGGTGA
ATCTGCCGCCGCACCTGCTCGATAACCAGGAACTTCCGGATCGACTCTATGAGTACGTCG
GCGCTCGCGGGGCTTGTACCAGCTCACTATGTTTCGAGTTGACCGAGAGCAGTGTCACAA
CTCTGTCAAGTAACTACTATGCAGGTGCCTGTCGCTTGCGCATGAAAGGGTTCGGATTGG
CCCAGGACGACTTTGGCCAGGGTTACAGCTCGTTCTATAACCTGGTCACGACGCCTTTCA
CGGAGCTGAAGATCGACCGCTCCCTAGTCCAGGGATGCGTAGAGGATAACGGCCTCAATG
CAGCTGTCATCAGTTGTATTGAGTTGGGTCACCGCCTGAATCTCGACGTGGTGGCCGAAG
GCGTGGAGACCTGCGAGGAACTGAATCTTCTTCGTCGTCTTGGCTGCGACCGGGCGCAGG
GTTTCCTGATTTCTAAGGCAGTGTCTGCTCGTGAGTTCGAGCGGCAGTTAAGGGAGGACG
GCCCCAGCCTCCTTGTT**TAA**CGCAGTATCCCCATTATCGCGGAGTCGATCGCAGCCAACC
ACCGTCAGCGCAACAGTGTCGCTGAAGGAGGCCGCATCCCGTGAAGTCTGCTAGTGCCTT
GGAGCACGACAACAAACTTTTGCTCAAATGGACAACCCTCTCGCAGAGCCTGAGCATCGG
CTTGATCTGTGTGGTGGTGCTGACCGTATTGCTGTTCAGCATCTGTTACTGGTCGCTGGG
GAGATTGTTTCAGGAGGAGGAGGACAAAGT

> pvrS
TGGCGGTTCGACCTGGCTCCTGCACGACGACGCTGTGTAGCAGCCCCTGCGACAGCCAAC
TGAGTTCGTACTCAAACATGGCCTGGGCATGCTTCCAGCCCGCGTCAGGCCAGAAGAAAT
ACAGGCATACCCACTCCACGATGATTGCGCCGATCAGCGACCCGAACATCACGCCGATTC
AGGAGCAGGAGTAGGGGGGCAATCGAAAATGCCAAGACTGGTGCCGTGGGTTTTCTGGGG
TAGCCGCGGGAGAATGAACAAAGATGTTCAAACGTTCCTATAGGAAACTGTGTTGTGAAC
TCCATAGAACTCCCCTTTTACACGATGCAGGTTGTTGCTTGGAGTGTGTATCCACTAGTT

CTCGGGGAGGCCGTCACTGTAGATTTTTTGGGGGGGTATCTCGGCATAGGGGGGATTGAA
TAGCGCTGTGCCTGCAGTCCTGAGACCTCATTAGGAATCATCTGATAGAGGGAATTTTTC
CATGCCGTCAGTCTTTCGGC**ATG**AAGTTGAAGAATTTCTTACAGCCTTTTGATAGCGGTT
TCTCCACTCCGAGTGCTGCGCTCAAGCTGCTCCGCATGCTCGGTGGCGCCTTGATGTTGT
GCGTGCTATGCAGCCTGATATTCAGTGTGAGCATGGTTTTAAACCATCAGGTGTCCCTCA
GTCGGCAAGCTATGAATGTGGCTATGTACGAAGCGCAGCTTTATTTCGAGCAGCGCGAGG
CGTTGCTCAATCACTTGAGCGGCAATGTCGTGCCCTTGGCCGCGGGTAGAGCGCTCGTCA
ACGAAGCGCCGAACAATGTGAGCATCCTGCCGTTGAGTGACGGAGGGCGAGGTCTGCTAT
TGACCGCTCGCACGCTCGGTGATCTCCGGGAAAAGCGGCTGGCACTGATGTATCTGGTCG
ATACCGACAAAGGCCCTCTGGTTTACCGGCTTACCGCCGATGGTAGGCCCTCGGCAGCGA
TATCCAGCACGATAACCAAAGAGGTGTACCGAGCCTTGCTGGCGACTCCGTCGGCGCCTG
TTCACTGGGTGACTGACGGTGGTACCCCTCAACGGCTGTACCTTTTTGAATCCTTAGGCG
ATGAGCCGGGCGAGGGGTGGCTAGGCCTGGAGATTCTCGGCGAAGACCTCGATTCGATGT
TGCGCCGGAATGATGCCGGAAACTACATGCTGCTGGATCAGCATGGGCAGGTCGTACTCG
CTACGGACGCAGAGGCGCTGGGGAGCGGTGCGTCGCGGACGCTTTTGCGTGGAGACGGCT
TCGGTTTCATCGGTGCTGGCCCACTGCCGCAGCATATGGTGCTTTTCCAGCACGTGGGGT
CTTCGAGCTGGGATCTGATCTATCACATCGGTATCGGTCGCCTGTTGCTGGCTCTGTGGC
TCCCTCTGTTACTTGCCTCTGCGTTGGCACTCGCAGTCGGCATCCTACTGCATTGGCTGG
TGCGGAGCATCGAGCGACGCTTGATAGAGCCCGCAAAGCGACGCCTTGAAGCATTGAAGG
AGAGCGAAGCCTTTTCCCGTGCAGTTATCCAGGCCGCGCCCGTCGCGCTGTGCGTGCTGC
GTCGTGCCGACGCCGCAGTGGTCCTGGAAAATCCCCAGGCGCGCCAATGGCTGGGTGATA
GCGAGGCGATTGCCCACGACGCGCCGAGATGGATTTCCCAGGCGTTCGCAGGAGGTGTGA
AGTGTTCTGGAGAAGAACTGGAAACCGAGGCAGGGCTACATCTTCATCTCAATTACACGC
CCACCCGCTATAACGGTGAAGACGTATTGTTCTGCGCCTTCAGTGAAATCAGTGCACGCA
AGCGGATGGAGGCGGAACTGGCTCGCGCAAAATCCCTGGCGGATGCTGCCAATGAAGCCA
AGACGCTGTTTCTCGCCACCATGAGCCATGAAATCCGCACACCTCTGTACGGCATGCTTG
GCACGCTTGAGCTGCTTGGGCGTACCGAGCTGAGTCGGCAGCAGGCCGGTTACCTAAAGG
CAATCCAGCATTCCTCGTCGACCCTGCTGCAACTGATCAGCGATGTGCTTGACGTATCCA
AGATAGAGGCCGGCCAACTGGACCTAGAGTGCGTGGAATTCTCCCCGCTGGAATTGACCG
AAGAGGTCGTGCAGTCGTTCACCGGTGCCGCGCAGGCCAAGGGGCTGCAGTTGTATACCT
GCCTCTCTGCGGAGCTGCCGCTGCGCATGCGGGGGGCCGCGGCGTCGATCCGGCAGATTC
TCAACAACCTGCTGAGCAACGCGGTGAAGTTCACCGACAATGGCTATGTCAACGTCCACC
TGAAGGCCAGCGTGGTCGATGCCGAATGTGTGATGCTGACCTGGCAGGTCAACGATACCG
GCATGGGGATCAACGTCGAGGATCAGCCGCGTCTGTTCGAACCGTTCTACCAGATACGCC
GCTCCGAGCATCCGGTCGCAGGCACGGGCCTCGGCTTGTCGATCAGCCAGCGCCTGGCGC
AGCTAATGAATGGCAGTCTGAAACTGGTCAGTGAGCTGGGGTTGGGCAGCAGCTTTAGCC
TCAGGCTTCCGCTTGAGCGGATCGCGATGCAGGCTGAGCCGCAGGACCTAGCCGGGTGCG
CCGTCCAAGTGCTGGCGCCTGTCCGCGACCTAACGGAATGCCTGTGTGGCTGGATCTCCC
GCTGGGGTGGAAGGGCCATGGTCGCGACGCCGAGGTCGCTGGACGAGGCGGACGCGACCT
CGCTGCTGGTCGAAGTGTTACTGCTGGAGGGGGCGCCGATGTTCGAAGCATGGCCAGGAT
GCCGGGTGGAGCTTTCCCCTCAGGGTGATATGGAGCCGCAGGCACAGGGCCGCGACTGGC
TGCTCGGGCTCAACAACCTGGACGGCCTGCATCGTGCTCTGGGCCTGGCCCATGGGCGTC
TCGCTGATCCTTCGACGCCGCCGATACGGCTGGCTCCGTTGCGCAATCTAGGTCTCCGCG
TCCTAGTGGTGGAGGATAACGCGATCAACCAGTTGATCTTGAGGGACCAGATGGAAGCGC
TGGGCTGCAGCGTGGAGCTGCTCTTCGATGGTCGCGAGGCGTTGCTGCACTGCCAGACGG
CCTGCTTCGACGTGGTGCTCACCGATATCAACATGCCGAACATGAACGGATACGAGCTAA
CCGCGGAGCTACGGCGCCAAGGGTTCCGGCAGCCGATCATCGGCGCGACGGCGAACGCCA
TGCGTGAGGAGCGCGAGCGCTGCATGTCCGCCGGGATGAACGATTGCCTGGTCAAACCGG
TGGATCTGAATGCCCTTCAGAACTGCTTGATTAATATTCTCAAGGTGGATCGA**TGA**GCTG
GAAATCCTATCGGGTGCTGGTGGTCGAAGATCAGCCGTTTCAGCGCGAATACCTGCTCAA
CCTGTTTCGCGAGCGCGGCGTGCAGTACCTGGTAGGTGCCGGCGACGGCGCGGAGGCGTT
GCGCTGCCTGAAGCAGGACAGGTTCGACCTGATCCTCAGCGATCTGATGATGCCGGGCAT
GGATGGTATCCAAATGATCCTGCAACTGCCGTATCTCAAGCATCGTCCGAAGCTGGCGCT
GATGAG

> ndvB (Q9I4H4)

CCCGGCAGGCCGGGTTGATGGCCAGGATGCGCGCCGCCATCACCTCGACCTTGGCCTTGC
CCACCGAGCCTTCGATGGCATGCACCTGGCGGTTGGTGTTGGTCACGCAGACATCGTCGA
GGTCGAACAGGGAAATCTCGCCGACGCCGCTGCGGGCCAGCGCTTCCGCCGCCCAGGAAC
CGACCCCGCCGATGCCGACCACCGCGACGTGGCTATCGGCCAGGCGTTGCAGCCCCTCGC

GACCGTACAACCGGCCGATACCGCCGAAGCGCTGTTCATCCACCTGCATCACCAACCACC
TCTGAAACCGAATCGCGGATTGCCGCGCATTATAAAAAGCCGCGGCCGCCGCGTCGCC
TGCGTTCTTACGGCTCGTCGCGCAGGCCGGACTAGACTTGCAACAGACCCGCCGGCCCGG
CGCCTGTACACCCCGGGGGGTGCGGGGTAGCATGCGCCGCCCCGTGTTCAGCGAAACATC
CGGAACCTGGTTTCATCTCT**ATG**TCTTCACGCAAGATCGGGCTCAACCTGGTGGTCATCG
TCGCCCTGGCCGCCCTCTTCACCGGCATCTGGGCCCTGTACAACCGTCCGGTCAGCGTAC
CGGACTGGCCGGAACGCATCTCCGGCTTCTCCTTCTCGCCGTTCCGCCTCAACCAGAACC
CGCAGAGCGGCCGCTACCCCAGCGCCGAACAGATGCGCACCGACCTGGAACTGGTCGCCC
GGCACACCCACAGCATCCGCACCTATTCGGTCCAGGGCGCGCTCGGCGACATCCCGGCGC
TGGCCGAGGCGTTCGGCCTGCGCGTCAGCCTGGGCATCTGGCTCGGCCCGGACCTGGCCA
GCAACGAGGCCGAGATCGCCCGCGCCATCCGCATCGCCAACGAGTCGCCGAGCGTGGTGC
GAGTGATAGTCGGCAACGAGGCGCTGTTCCGCCGCGAGGTGACGGCGGAACAGTTGATCG
CCTACCTCGACCGGGTCCGCGCGGCGGTCAAGGTTCCGGTGACCACCGCCGAACAGTGGC
ACGTCTACCGCGAACACCCGGAACTGGCGCAACACGTCGACCTGATCGCCGCCCACGTCC
TGCCCTACTGGGAGGCCACGCCGGTGGCCGACGCGGTGGACTTCGTGCTCGAACGCGCGC
GCGAACTCAAGGCCGCCTTCCCGAGGAAGCCGCTGCTGCTCGCCGAGGTCGGCTGGCCGA
GCAACGGGCGCATGCGCGGCAGCGCCGAGGCGACACCCGCGGACCAGGCCATCTACCTGC
GGCGCCTGACCAACGCGCTCAACGGCGAAGGCTACAGCTACTTCGTCATCGAAGCCTTCG
ACCAGCCCTGGAAGGTCAGCGCCGAAGGCTCGGTGGGCGCCTACTGGGGCGTCTACAACG
CCGACCGCAAGGCCAAGTTCAACTTCACCGGGCCGGTGGTGCCGATTCCCAAGTGGCGCG
CCCTGGCCATCGCCTCGGCGGTACTCGCGGTACTCGCCTTCACCCTGCTGCTGATCGACA
GTTCCTCGCTGCGCCAGCGCGGGAGGACCTTCCTCGCCGTGGTCTCGTTCGCCTGCGCCT
CGGTGCTGGTGTGGATCGCCTACGACTACAGCCAGCAGTACAGCACCTGGTTCAGCCTGA
CCGTCGGCGCGTTGCTGGGCGTCGGCGCGCTAGGGGTGGTCATCGTGCTGTTCACCGAGG
CCCACGAGCTGGCCGAGGCGGTCTGGACGCGCAAGCGGCGCCGGCCATTCCTGCCGATCA
CCGCCGCGCGGGCCTATCGGCCCAAGGTGTCGATCCACGTGCCCTGCTACAACGAGCCGC
CGGAACTGCTGAAGCAGACCCTCGACGCCCTTGCCCGCCTCGACTACCCGGACTACGAAG
TCCTGGTGATCGACAACAACACCCGCGACCCGGCCGTCTGGCAGCCGGTCGAGGCGCACT
GCGCGCGCCTGGGCGAGCGCTTCCGCTTCTTCCACGTTGCCCCGCTGGAAGGCTTCAAGG
CCGGCGCGCTGAACTTCGCCCTGGGCCACGTGGCGGCGGACGTCGAGGTGGTCGCGGTGA
TCGACGCCGACTACTGCGTCGACCCCGACTGGCTCAGGCACATGGTGCCGCACTTCGGCG
ACCCGCGGATCGCCGTGGTGCAGTCGCCGCAGGACTACCGCGACCAGCACGAGAGCGCCT
TCAAGCGGCTCTGCTACGCCGAGTACAAGGGCTTCTTCCACATCGGCATGGTCACCCGCA
ACGACCGCGACGCGATCATCGAGCACGGCACCATGACCATGATCCGGCGCAGCGTGCTGG
ACGAGCTGAGATGGCCGGAATGGTGCATCACCGAGGACGCCGAGCTGGGCCTGCGGGTGT
TCGAGAAGGGCCTGTCGGCCGCCTACTTCGAGCGCAGCTACGGCAAGGGGGTGATGCCCG
ATACCTTCATCGATTTCAAGAAGCAGCGCTTCCGCTGGGCCTACGGCGCGATCCAGATCA
TGAAGCGGCATACCGACGCCCTGCTGCGCGGCCGCGGTCCCGACGGCAGCCGCCTGACCC
GCGGCCAGCGCTACCACTTCGTGGCCGGCTGGCTGCCGTGGATCGCCGACGGCCTGAACA
TCTTCTTCACCCTCGGCGCGCTGCTCTGGTCGGCGGCGATGATCATCGTGCCCAAGCGCG
TCGACCCGCCGCTGCTGATCTTCGCGATCCTGCCGCTGGCCCTGTTCGTCTTCAAGGTCG
GCAAGATCCTCTTCCTCTACCGGCGCACCGTCGGCGTCGACCTGCGCGACTCGTTCTTCG
CCGCCCTCGCCGGCCTGTCGCTCTCGCACACCATTGCCAAGGCGGTGCTGTACGGCTTCG
TCACCCGCGGCATCCCGTTCTTCCGCACGCCGAAGATGCGCTCCAGCCACGGCCTGCTGG
TGGCCCTGGCGGAGGCCCGCGAGGAAGTCTTCGTGATGCTCCTGCTGTGGGGCGCGGCGG
CCGGCATCGTGGCGGTTCAGGGCGTGCCGAGCCGCGACCTGCTGATCTGGGTCGCCATGC
TCCTGGTGCAATCGCTGCCCTACCTGGCGGCGCTGGTCATGGCCTTGCTCTCGTCGCTGC
CGAAACCGCGCGAGGAACTGGCCGGCGGCGCCGAGCAGATCGGCGGT**TGA**GGTCGGGCGA
GGCGTCGGGACGGCCGGCCCCGCTCGCCTAAACGGCGTCCCTTCGCCCACGTTTATGCTT
TAAGATTCCCCCTTTCCGCTCGACCCGCTCAGGATGCCCTCCCCATGACCGCCTCTTCGC
CGAGTCTCTCGCCGACCCTCGAACTCGCCTGCGAGTTGATCCGCCGCCCCTCCGTCACGC
CGCTCGACGCCGACTGCCAGGCCCTGATGATGCGCCGCCTGGAAGCCGCCGGCTTCGCCC

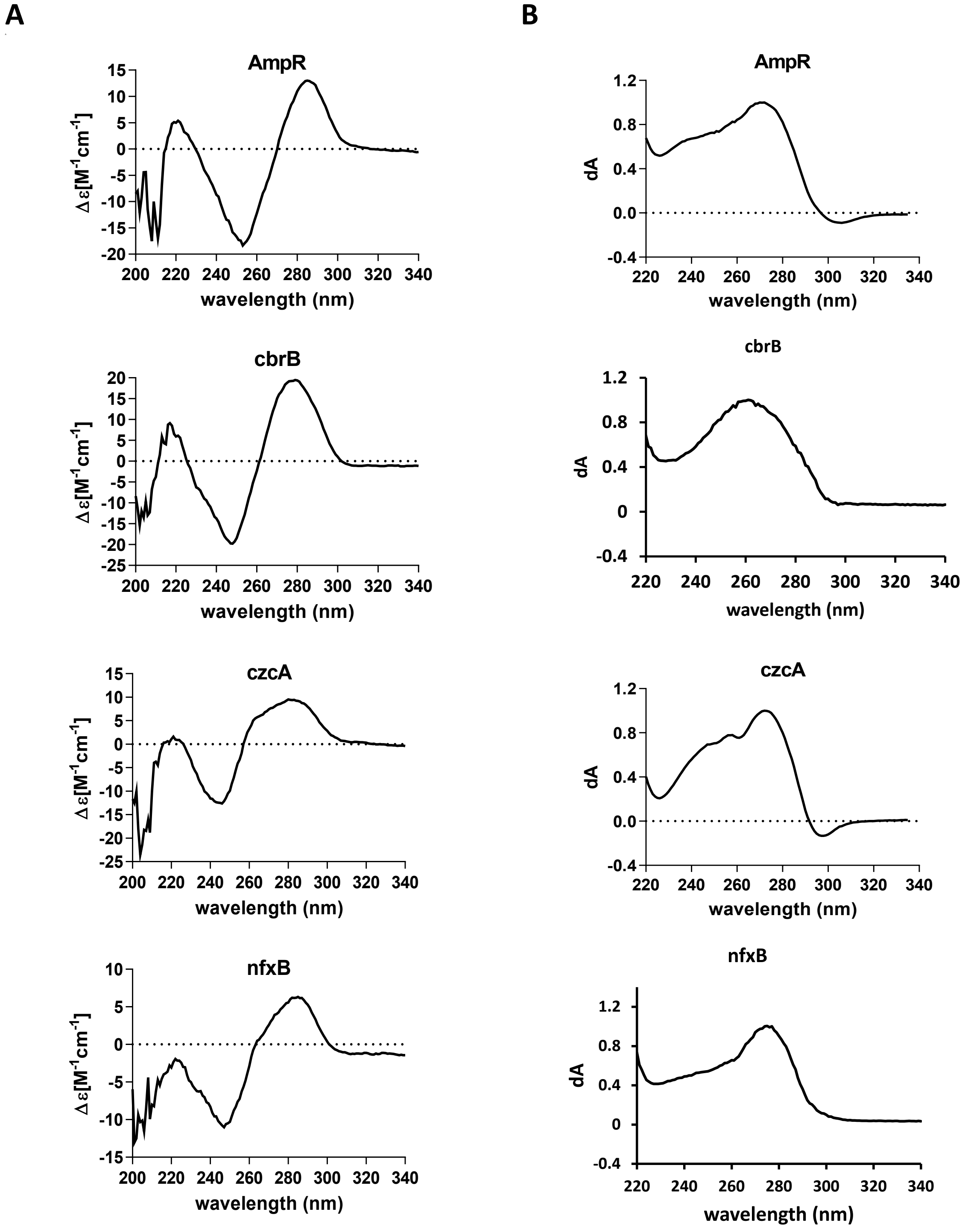
A
AmpR
Δε[M-1cm-1]
wavelength (nm)
cbrB
Δε[M-1cm-1]
wavelength (nm)
czcA
Δε[M-1cm-1]
wavelength (nm)
nfxB
Δε[M-1cm-1]
wavelength (nm)
B
AmpR
dA
wavelength (nm)
cbrB
dA
wavelength (nm)
czcA
dA
wavelength (nm)
nfxB
dA
wavelength (nm)

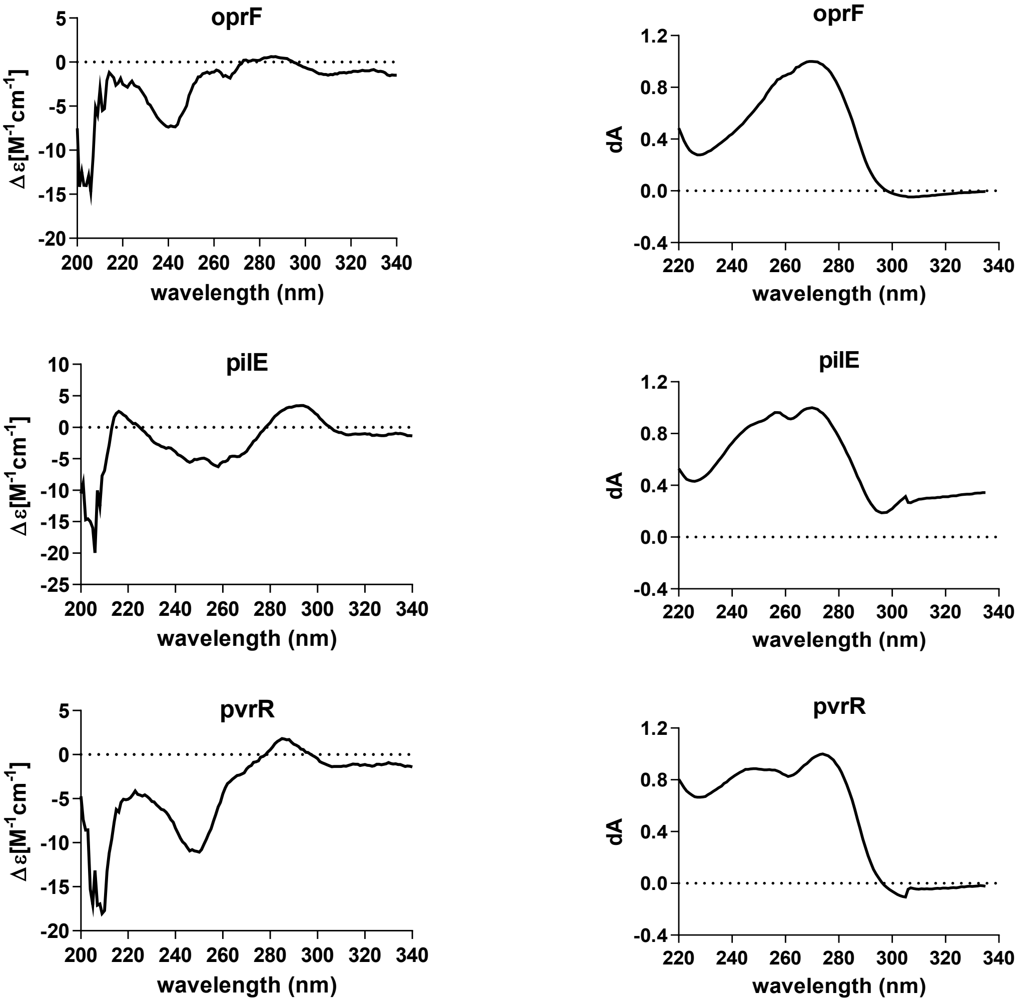

Figure S1. Spectroscopic characterisation, (a) CD spectra (b) thermal difference spectra, of DNA sequences not identified as forming quadruplexes.

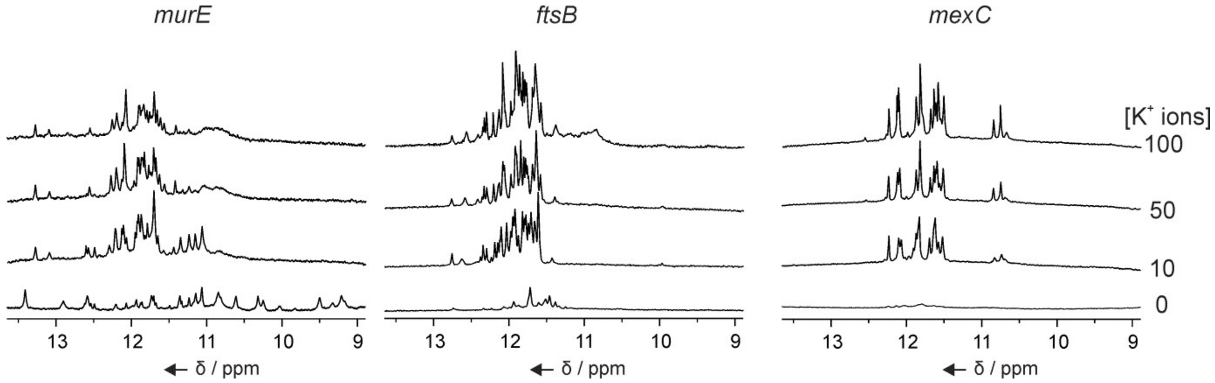

Figure S2. Imino region of ^{1}H NMR spectra of *murE*, *ftsB* and *mexC* oligonucleotides in the absence and presence of 10-100 mM KCl as indicated on the right side of the spectra. NMR spectra were recorded at 0.4 mM DNA concentration per strand, pH 7.4, 25 °C, on a 600 MHz NMR spectrometer.

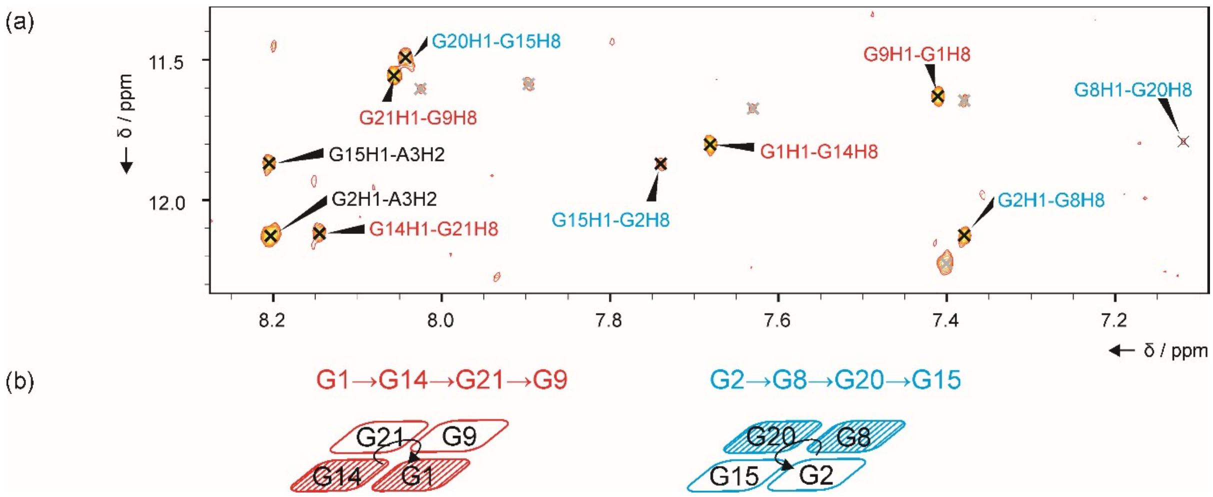

Figure S3. (a) Imino-aromatic region of the NOESY (τ_m 350 ms) spectrum of *mexC*. The H1-H8 cross-peaks within G1-G14-G21-G9 and G2-G8-G20-G15 quartets are designated in red and blue, respectively. Cross-peaks marked with black correspond to interactions between A3 H2 and G2-G8-G20-G15 quartet. Signals assigned to the minor species are marked with grey. NMR spectrum was recorded at 0.4 mM concentration of *mexC* per strand, 100 mM KCl, pH 7.4, 25 °C, on an 800 MHz spectrometer. (b) Representation of the clockwise and anti-clockwise donor-acceptor hydrogen-bonding directionality in G1-G14-G21-G9 and G2-G8-G20-G15 quartets, respectively. *Syn* guanines in G-quartets are striped.

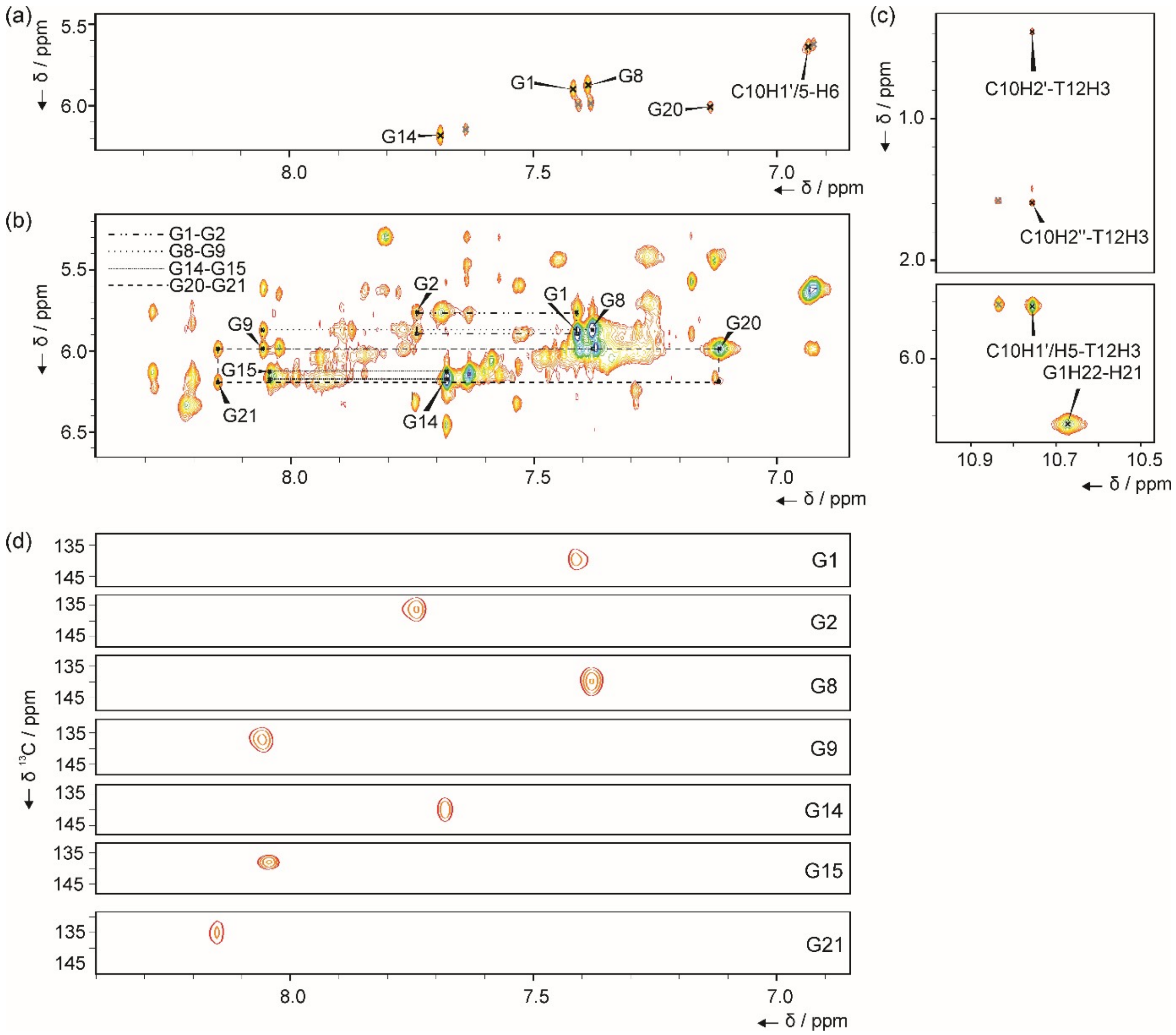

Figure S4. (a) Four distinct and strong cross-peaks in anomeric-aromatic region of NOESY spectrum (τ_m 100 ms) of *mexC* showing that G1, G8, G14 and G20 residues adopt *syn* conformations along glycosidic torsion angles. Signals assigned to the minor species are marked with grey. (b) Anomeric-aromatic region of NOESY spectrum (τ_m 200 ms) of *mexC* with marked sequential NOE cross-peaks between G1-G2, G8-G9, G14-G15 and G20-G21 residues, characteristic for 5'-syn-anti-3' steps. The NOE connectivities are marked with different line styles. (c) NOE cross-peaks between T12 H3 and C10 H1', H2', H2" in NOESY spectrum (τ_m 200 ms) of *mexC*. Signals assigned to the minor species are marked with grey. NMR spectra were recorded at 0.4 mM concentration of *mexC* per strand, 100 mM KCl, pH 7.4, 25 °C on an 800 MHz spectrometer. (d) Aromatic region of 2D ^{1}H-^{13}C HSQC spectra of *mexC*. The HSQC spectra were acquired on partially (10%) residue-specifically ^{15}N- and ^{13}C-labeled oligonucleotides. Assignment of H8 proton resonances of the major G-quadruplex adopted by *mexC* is indicated on the right side of the spectra. NMR spectra were recorded at ~0.5 mM concentration of *mexC* per strand, 100 mM KCl, pH 7.4, at 25 °C, on a 600 MHz spectrometer.

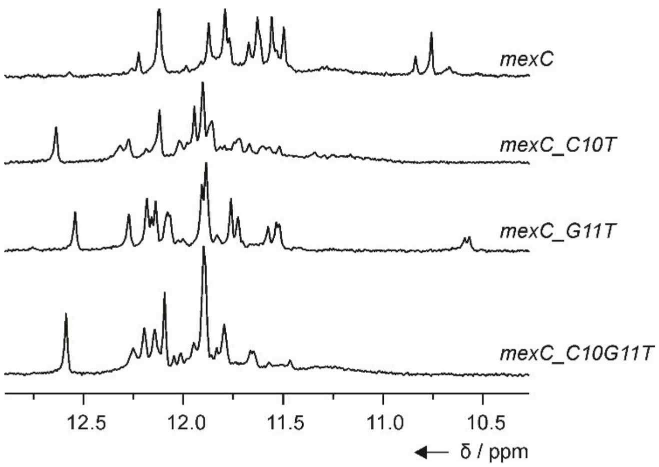

Figure S5. Imino region of the ^{1}H NMR spectra of *mexC* and its analogs, where C10 and/or G11 were replaced with thymine residues in *mexC_C10T*, *mexC_G11T* and *mexC_C10G11T*. NMR spectra were recorded at ~0.4 mM concentration of oligonucleotide per strand, 100 mM KCl, pH 7.4, at 25 °C, on a 600 MHz spectrometer.

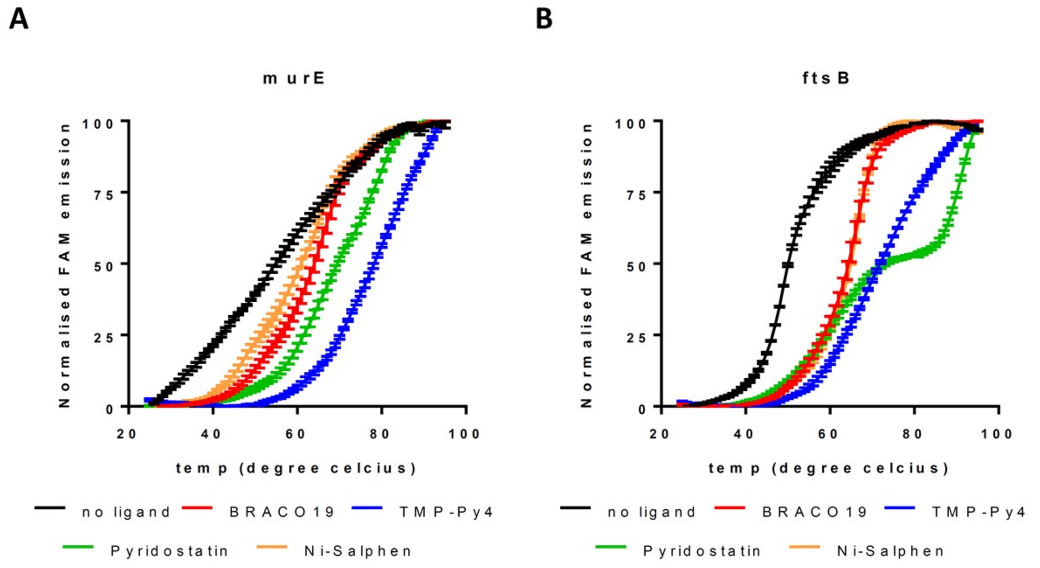

Figure S6. Example of FRET melting curves for (a) *murE* and (b) *ftsB* in the presence of no ligand, BRACO19, TMP-Py4, pyridostatin and Ni-salphen.
